# Supplementary figures and images for: Chromosomal Mcm2-7 distribution and the genome replication program in species from yeast to humans
Source: PLoS Genet. 2021 Sep 2;17(9):e1009714. doi: 10.1371/journal.pgen.1009714 (PMC8443269; doi:10.1371/journal.pgen.1009714)

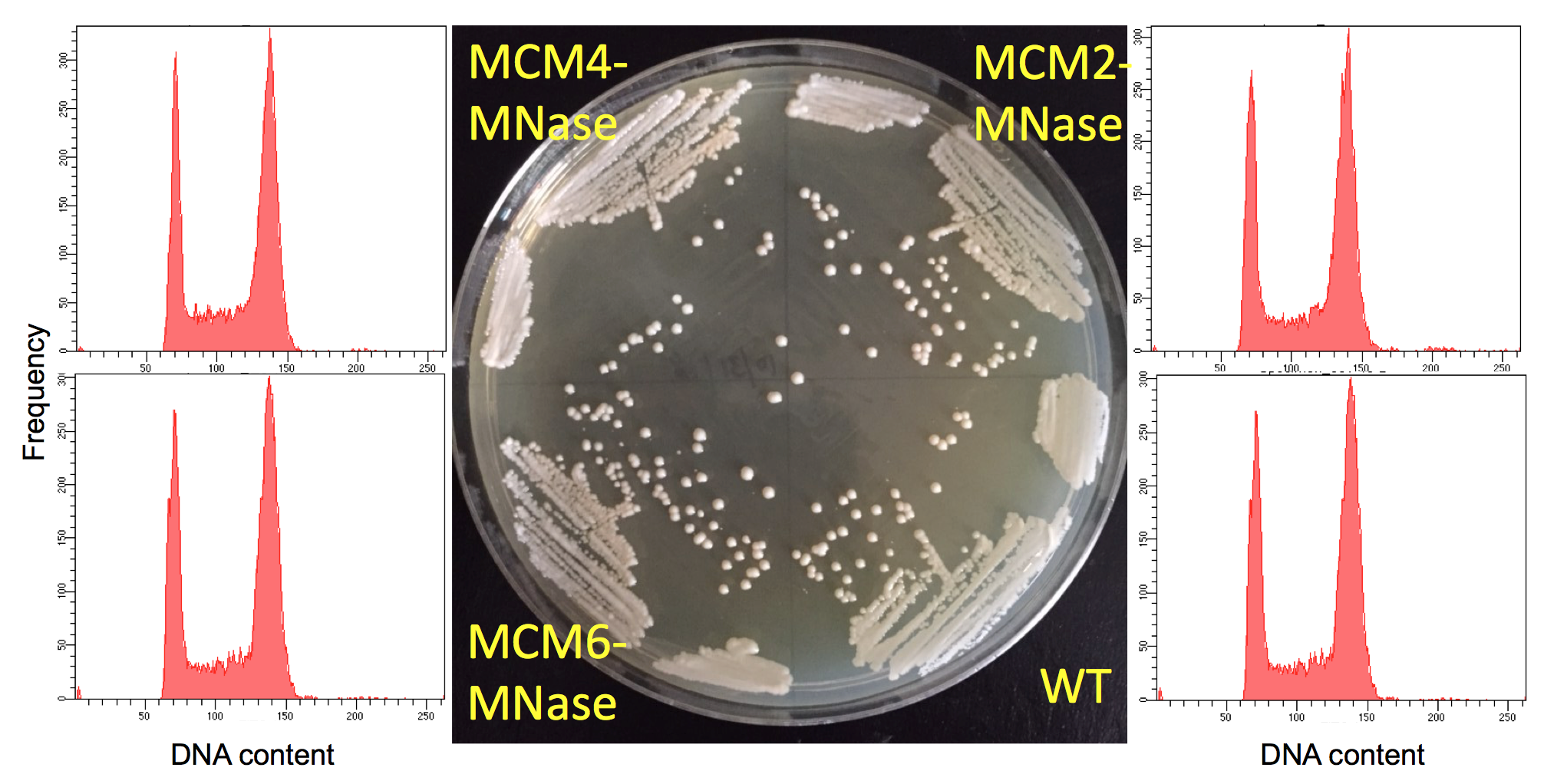

Supplement: S1 Fig — Flow cytometry profiles show log phase cultures stained for DNA content, with the two major peaks representing cells in G1 and G2. (TIF) [file pgen.1009714.s001.tif]

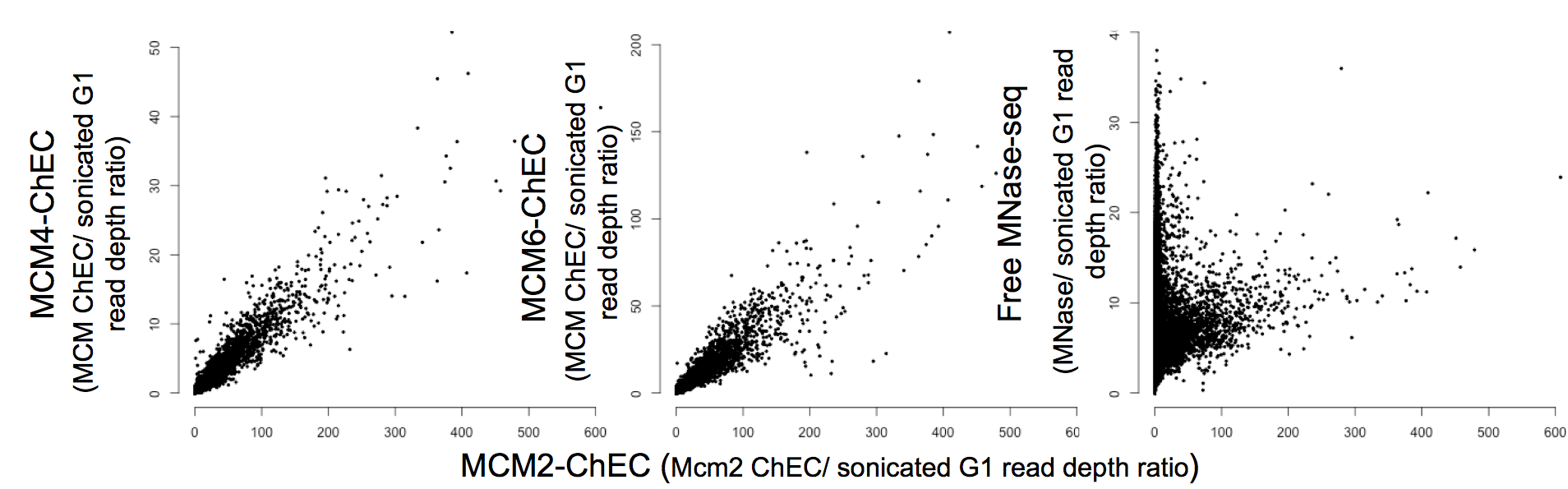

Supplement: S2 Fig — Genome-wide signal, in 100 base pair bins, plotted with Mcm2-ChEC on the x axis in all three cases, and on the y axis either Mcm4-ChEC (r = 0.96), Mcm6-ChEC (r = 0.94) or free MNase (r = 0.09). All fragment sizes were included in the calculations. (TIF) [file pgen.1009714.s002.tif]

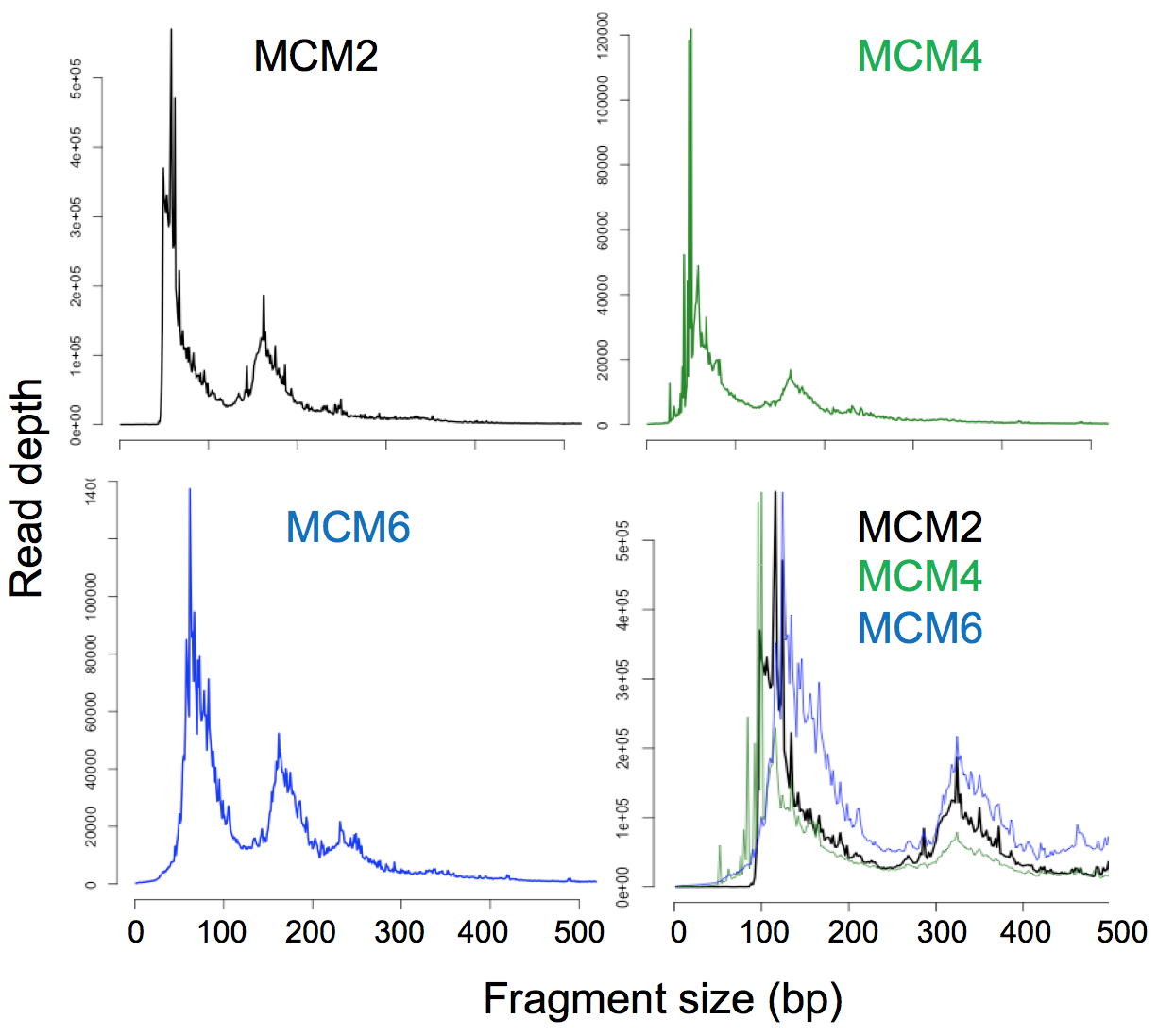

Supplement: S3 Fig — Fragment sizes for the peaks of Mcm2-, Mcm4- and Mcm6-tagged strains are 58, 50 and 62 base pairs, respectively, and median fragment lengths for fragments < = 100 base pairs were 61, 58 and 70 base pairs, respectively. (TIF) [file pgen.1009714.s003.tif]

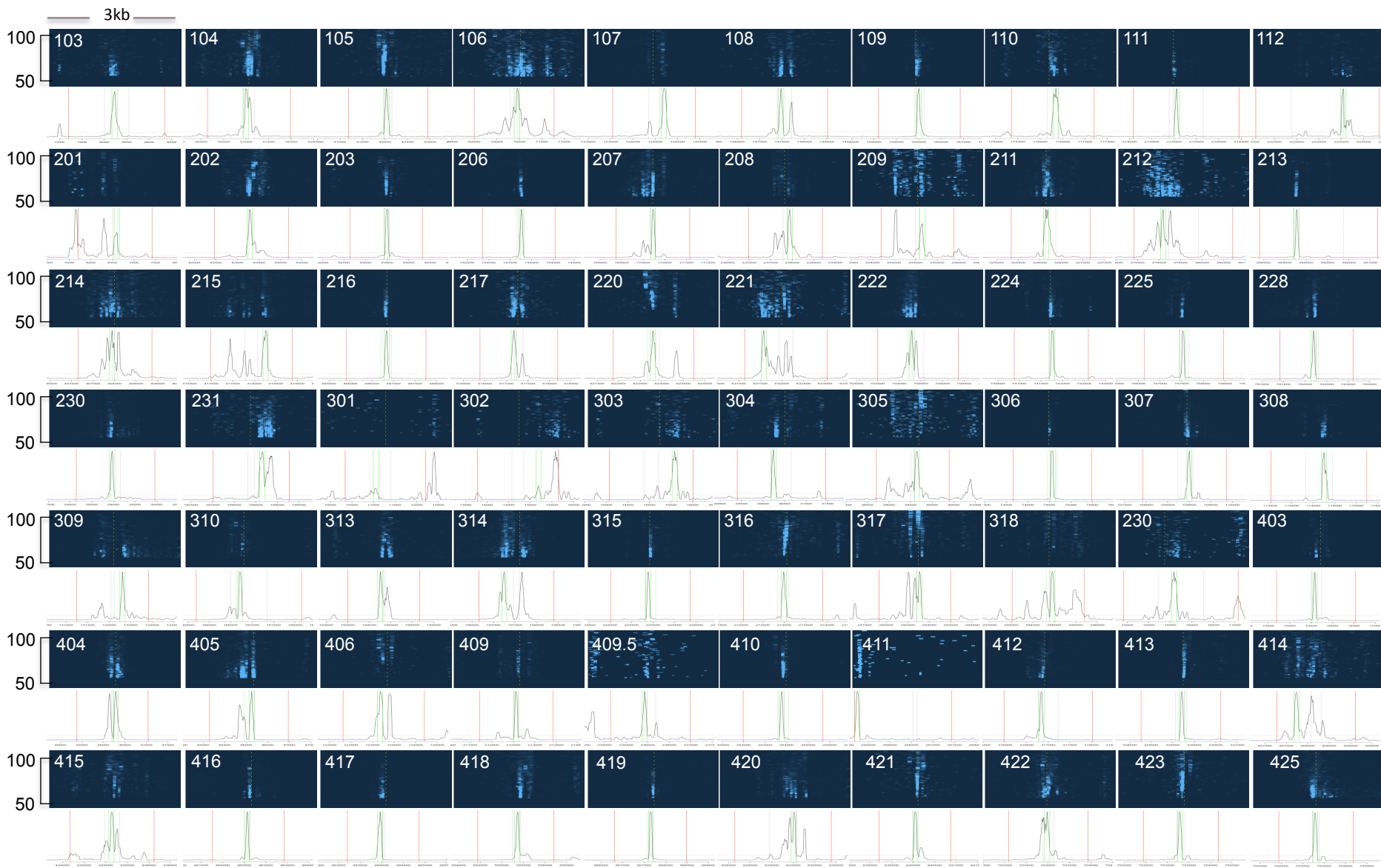

Panel 1

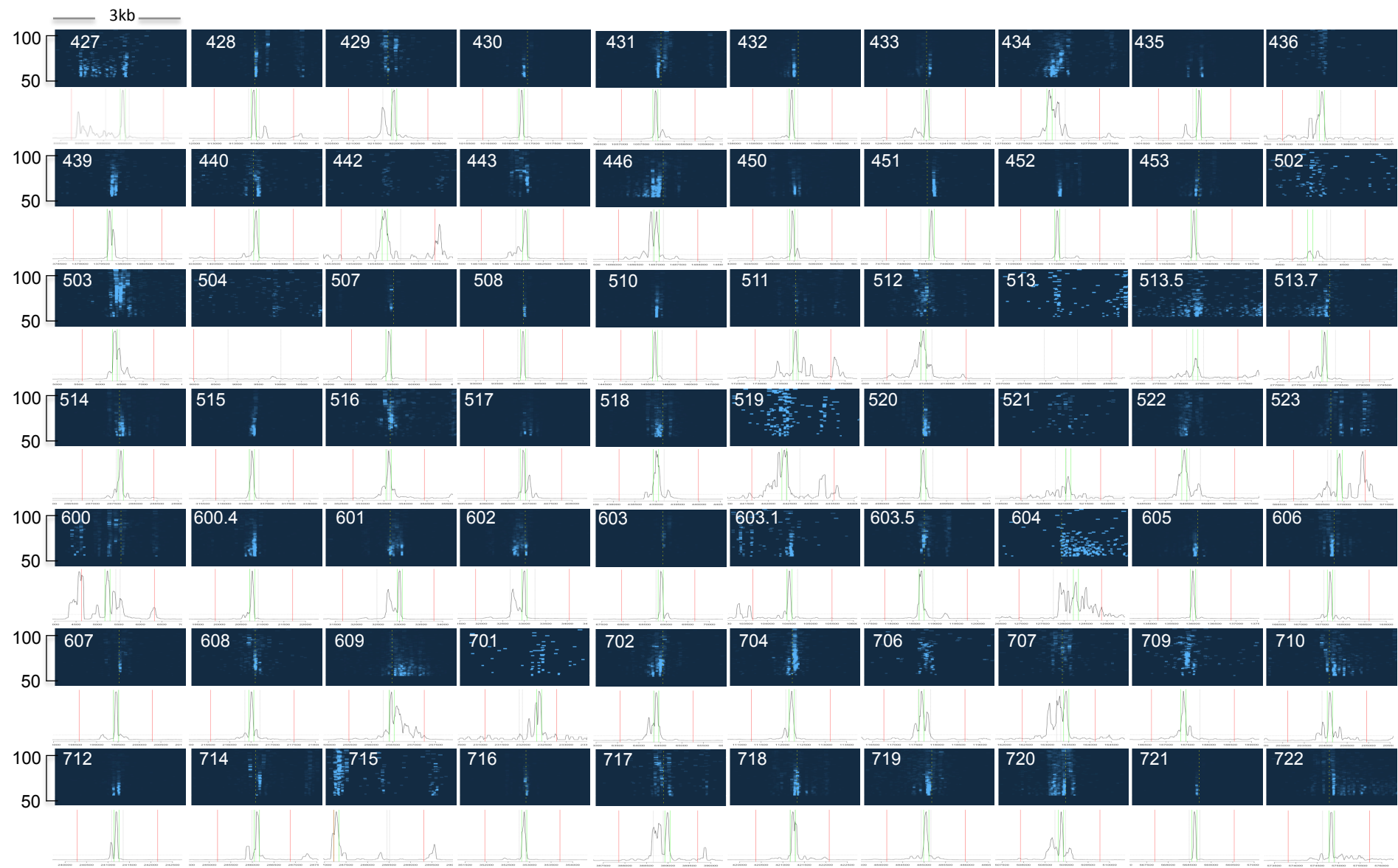

Panel 2

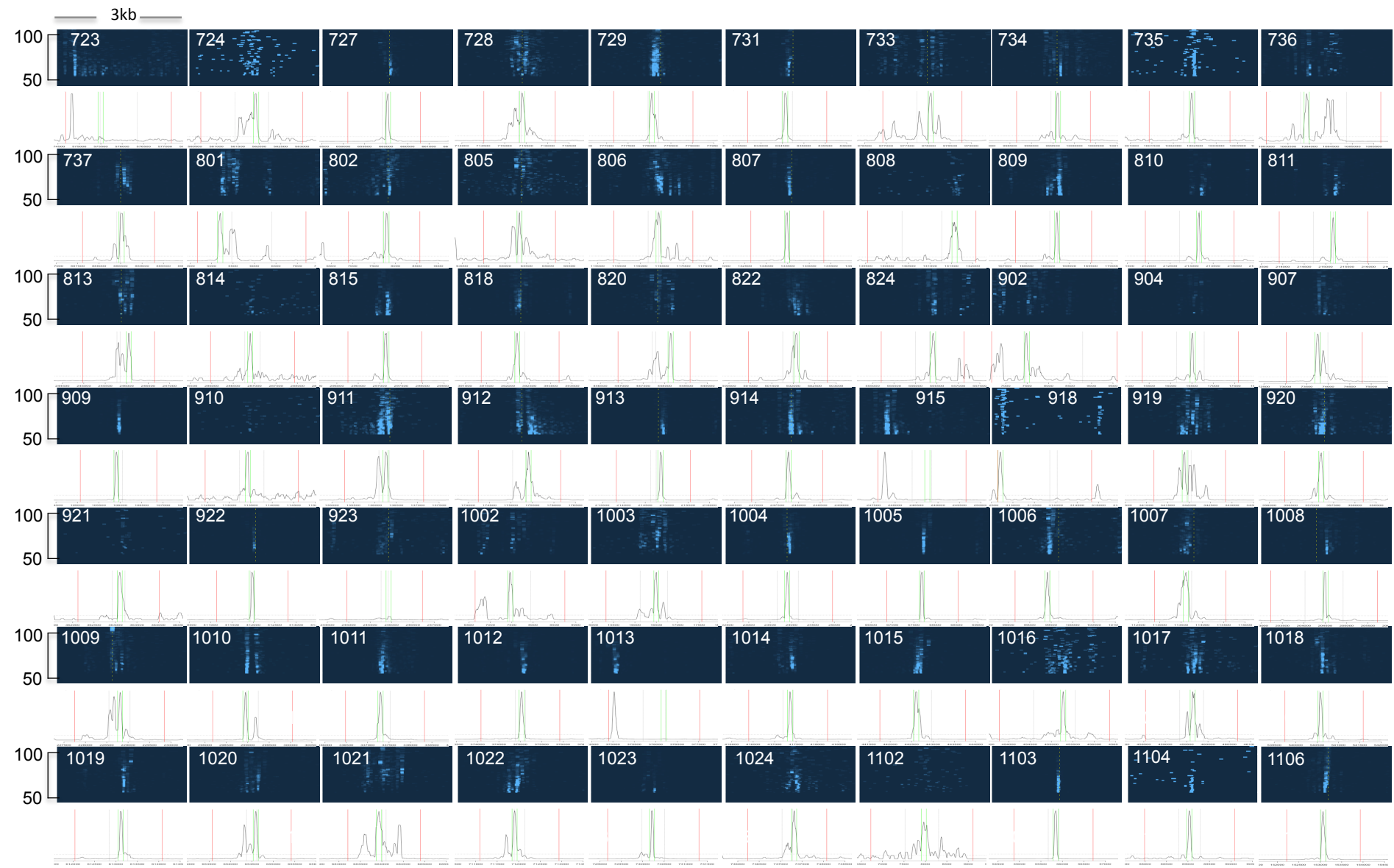

Panel 3

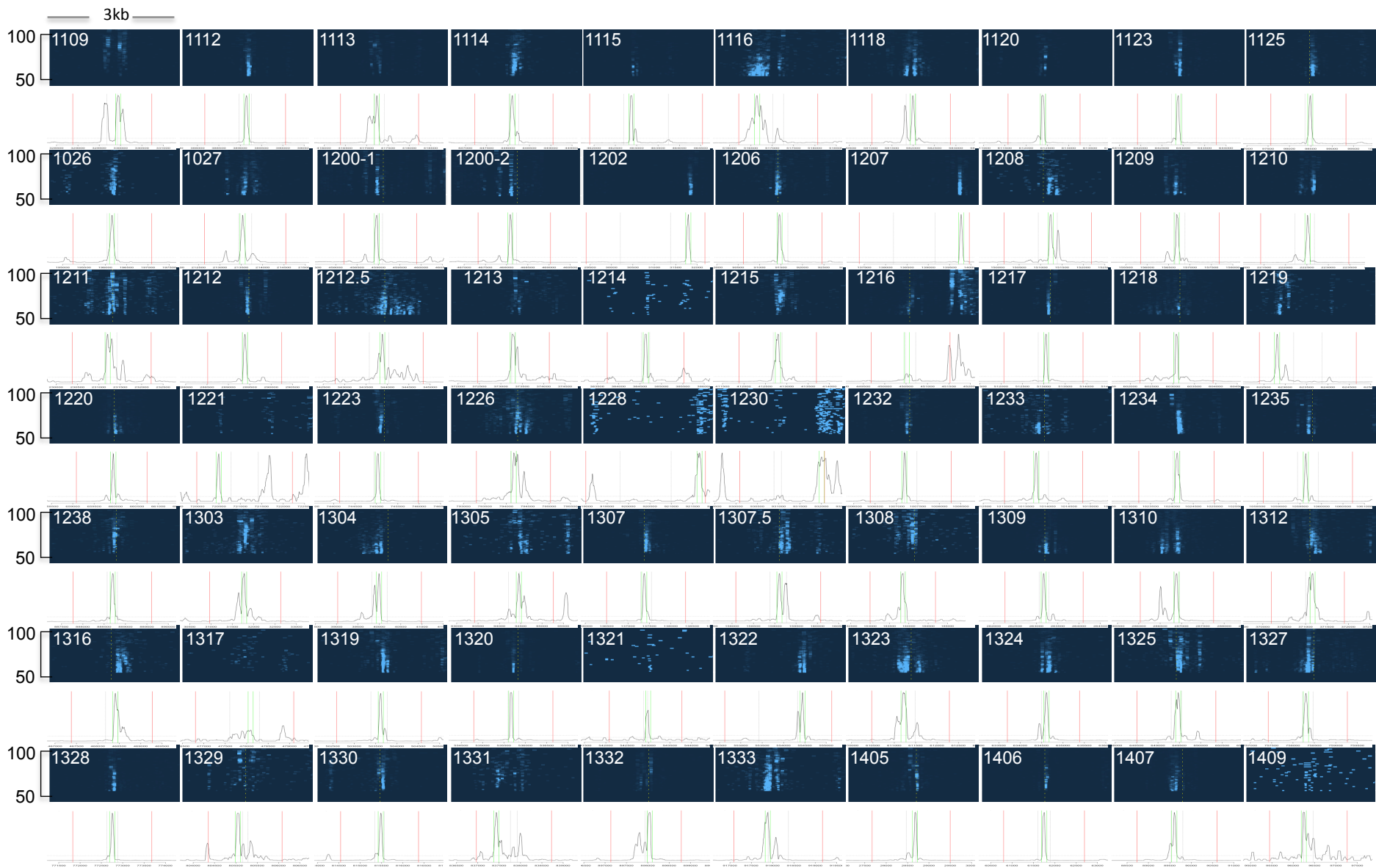

Panel 4

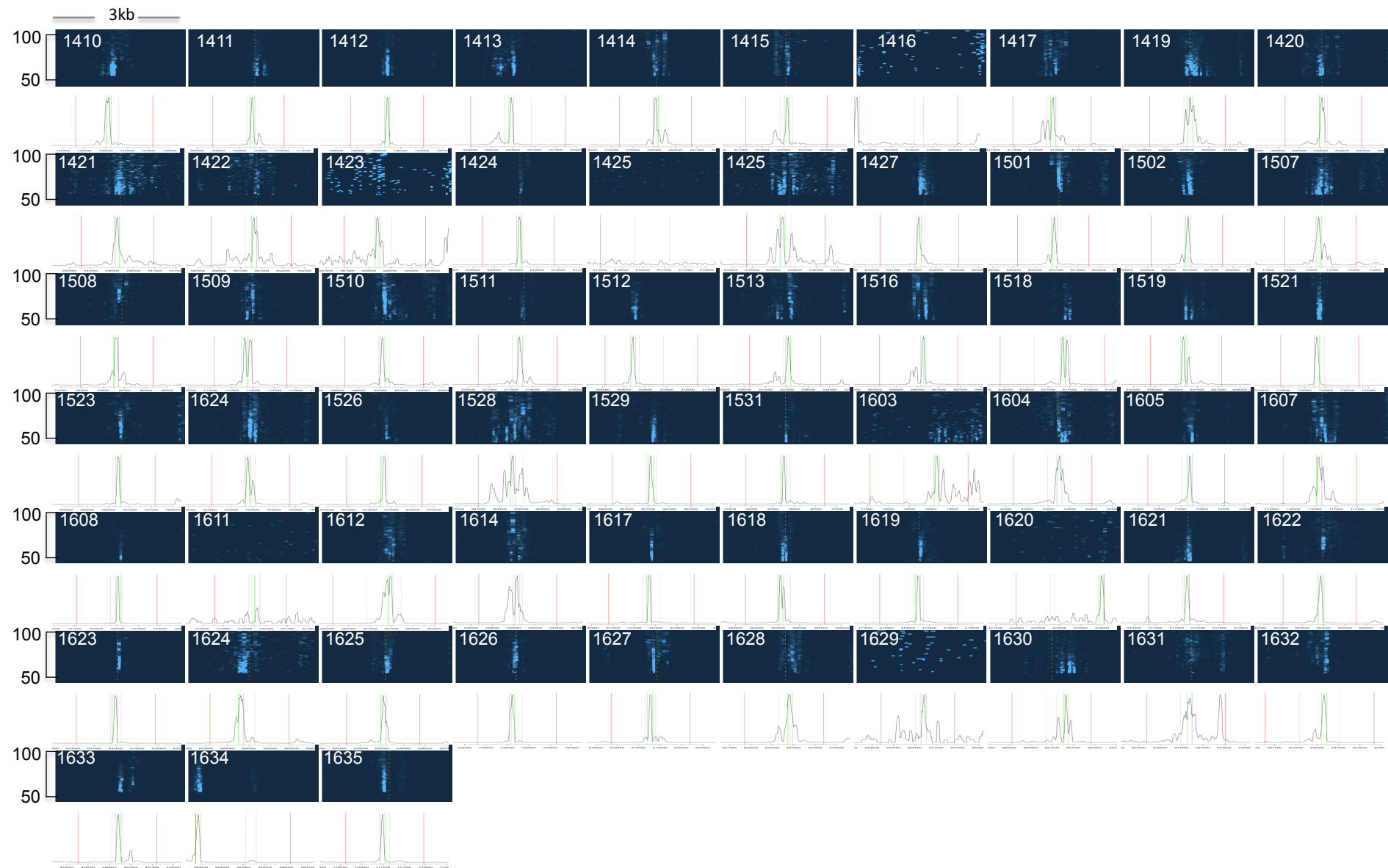

Panel 5

Supplement: S4 Fig — The markings on the plots are the same as for the 12 origins on chromosome IV in Fig 3. 9 origins listed in SGD were omitted from this analysis due bioinformatic difficulties, such as ambiguous mapping locations. 187 of the remaining 343 origins contained ACS sequences, and these are indicated with dotted lines. Each figure shows a 3 kb span on the X axis. Read depths are generally capped by setting the lightest color (highest read depth) to a per-base pair read depth that is less than the maximum read depth. This is done to enhance lower intensity signals. Capped read depths for each origin are listed here with maximum (uncapped) read depths following in parentheses: ARS1002: 160 (1285); ARS1003: 17 (143); ARS1004: 121 (975); ARS1005: 144 (1152); ARS1006: 12 (97); ARS1007: 53 (430); ARS1008: 69 (559); ARS1009: 56 (449); ARS1010: 26 (210); ARS1011: 128 (1025); ARS1012: 161 (1293); ARS1013: 161 (1293); ARS1014: 48 (384); ARS1015: 78 (631); ARS1016: 3 (27); ARS1017: 9 (77); ARS1018: 64 (513); ARS1019: 63 (510); ARS1020: 74 (593); ARS1021: 29 (234); ARS1022: 38 (304); ARS1023: 406 (3250); ARS1024: 21 (169); ARS103: 23 (190); ARS104: 41 (332); ARS105: 19 (159); ARS106: 14 (113); ARS107: 85 (683); ARS108: 16 (135); ARS109: 100 (804); ARS110: 15 (121); ARS1102: 7 (7); ARS1103: 397 (3177); ARS1104: 1 (14); ARS1106: 75 (603); ARS1109: 55 (443); ARS111: 658 (5270); ARS1112: 130 (1044); ARS1113: 94 (752); ARS1114: 39 (315); ARS1115: 80 (647); ARS1116: 24 (199); ARS1118: 52 (416); ARS112: 34 (275); ARS1120: 107 (858); ARS1123: 111 (892); ARS1125: 67 (541); ARS1126: 7 (56); ARS1127: 67 (542); ARS1200-1: 6655 (53244); ARS1200-2: 5457 (43658); ARS1202: 173 (1390); ARS1206: 65 (522); ARS1207: 104 (832); ARS1208: 6 (50); ARS1209: 106 (854); ARS1210: 72 (578); ARS1211: 13 (111); ARS1212: 81 (653); ARS1212.5: 12 (98); ARS1213: 40 (326); ARS1214: 1 (8); ARS1215: 21 (169); ARS1216: 162 (1302); ARS1217: 146 (1175); ARS1218: 131 (1055); ARS1219: 11 (95); ARS1220: 219 (1758); ARS1221: 5 ( [file pgen.1009714.s004.PDF]

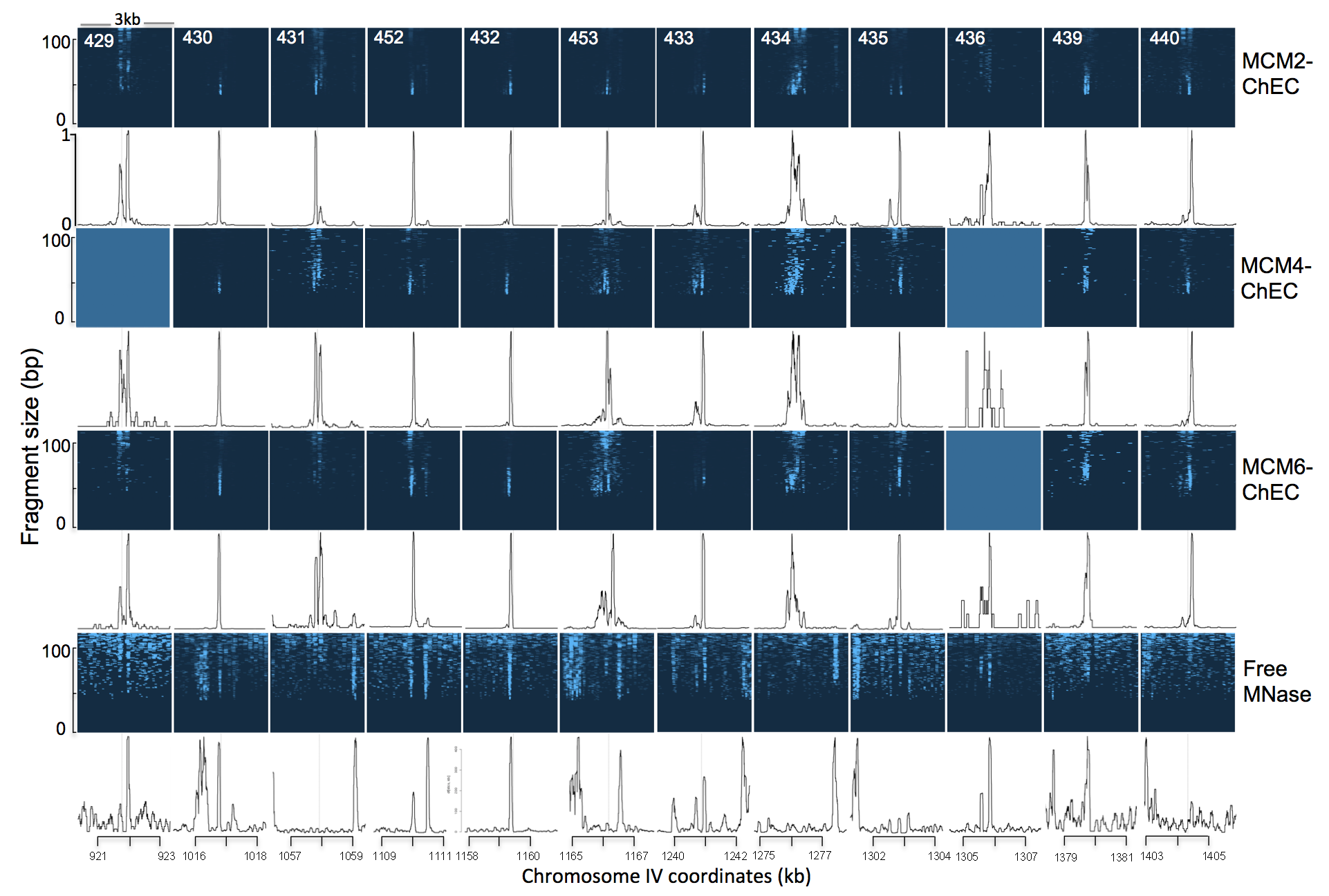

Supplement: S5 Fig — Footprints for Mcm2-ChEC (top row), Mcm4-ChEC (second row), and Mcm6-ChEC (third row) resemble each other at each of 12 consecutive origins on chrIV, but free MNase (bottom row) does not. Below each heat map is a relative distribution of read depths for the 50–100 bp size range. Capped and uncapped read depths for MCM2 are described and listed in the legend to S4 Fig. Corresponding numbers for MCM4 and MCM6 are listed below. MCM4 ARS429: 7 (7); MCM4 ARS430: 29 (237); MCM4 ARS431: 2 (17); MCM4 ARS432: 48 (388); MCM4 ARS433: 5 (43); MCM4 ARS434: 1 (15); MCM4 ARS435: 3 (31); MCM4 ARS436: 2 (2); MCM4 ARS439: 1 (10); MCM4 ARS440: 4 (32); MCM4 ARS452: 5 (42); MCM4 ARS453: 5 (40); MCM6 ARS429: 3 (24); MCM6 ARS430: 77 (622); MCM6 ARS431: 10 (83); MCM6 ARS432: 116 (930); MCM6 ARS433: 83 (664); MCM6 ARS434: 4 (35); MCM6 ARS435: 6 (48); MCM6 ARS436: 2 (2); MCM6 ARS439: 1 (13); MCM6 ARS440: 3 (28); MCM6 ARS452: 7 (63); MCM6 ARS453: 5 (45). (TIF) [file pgen.1009714.s005.tif]

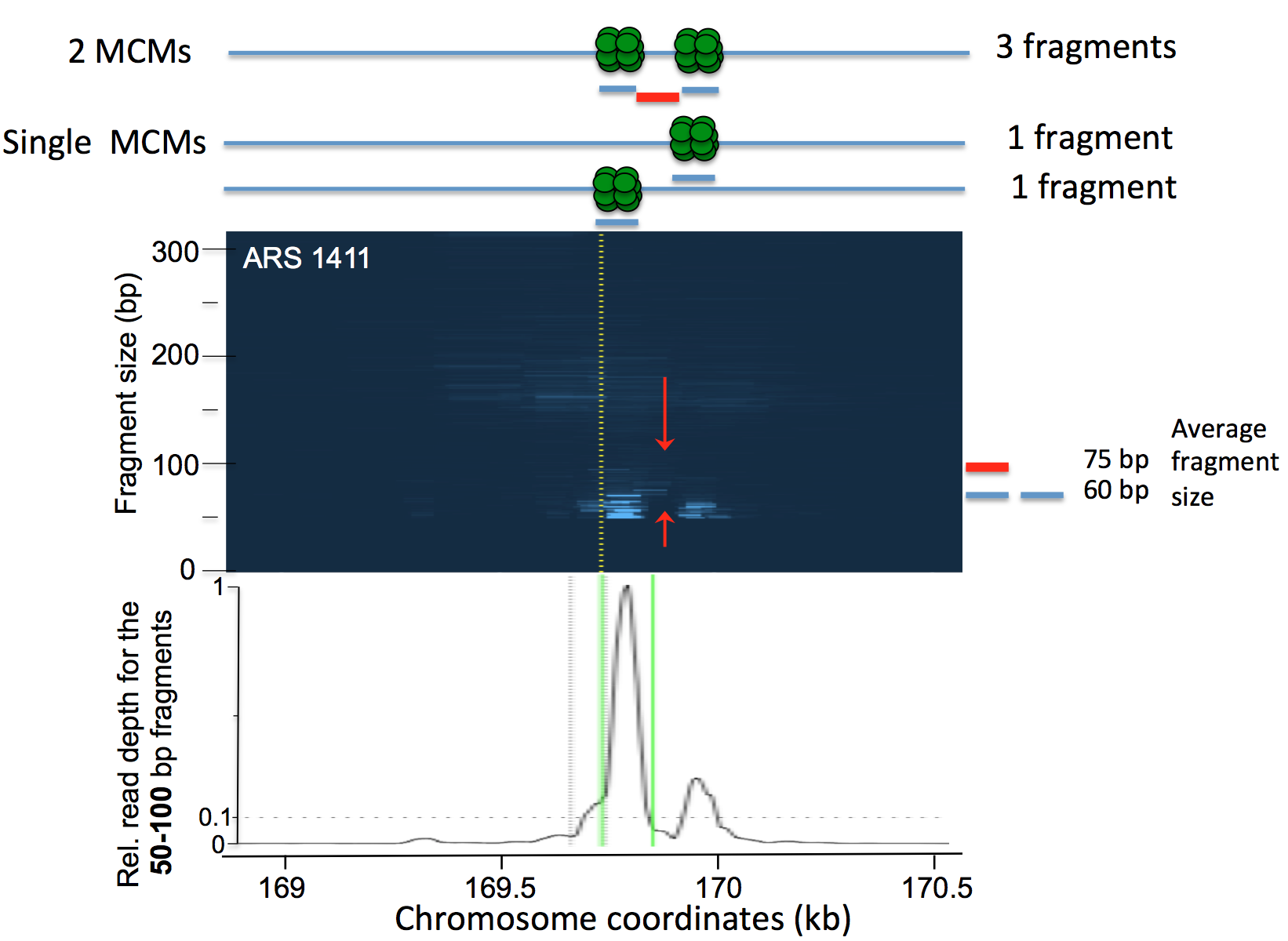

Supplement: S6 Fig — The heat plots that we use to visualize MCM binding in Figs 1B, 2A, 3A, S4 and S6 often show more than one adjacent MCM footprints. Because these plots reflect the composite binding of all of the cells in the population, the presence of multiple MCM signals need not indicate that more than one MCM DH binds within the same cell. Multiple MCM binding events within the same cell should generate fragments that span the region between those sites (red fragment in the top image). In the plot shown (ARS1411), this fragment should be approximately 75 base pairs long, as indicated by its position on the y axis; its absence (space between two red arrows) indicates that the two MCM DH footprints at this origin mostly reflect signals that emanate from two populations of cells, each with a single MCM DH. However, we cannot exclude the possibility that the 75 bb fragment could have been degraded. The single vertical dotted yellow line indicates the position of the ACS. The pairs of vertical gray and green lines delineate the ARS boundaries, as reported in SGD, and 60 base pairs on either side of the peak of Mcm2-ChEC signal. (TIF) [file pgen.1009714.s006.tif]

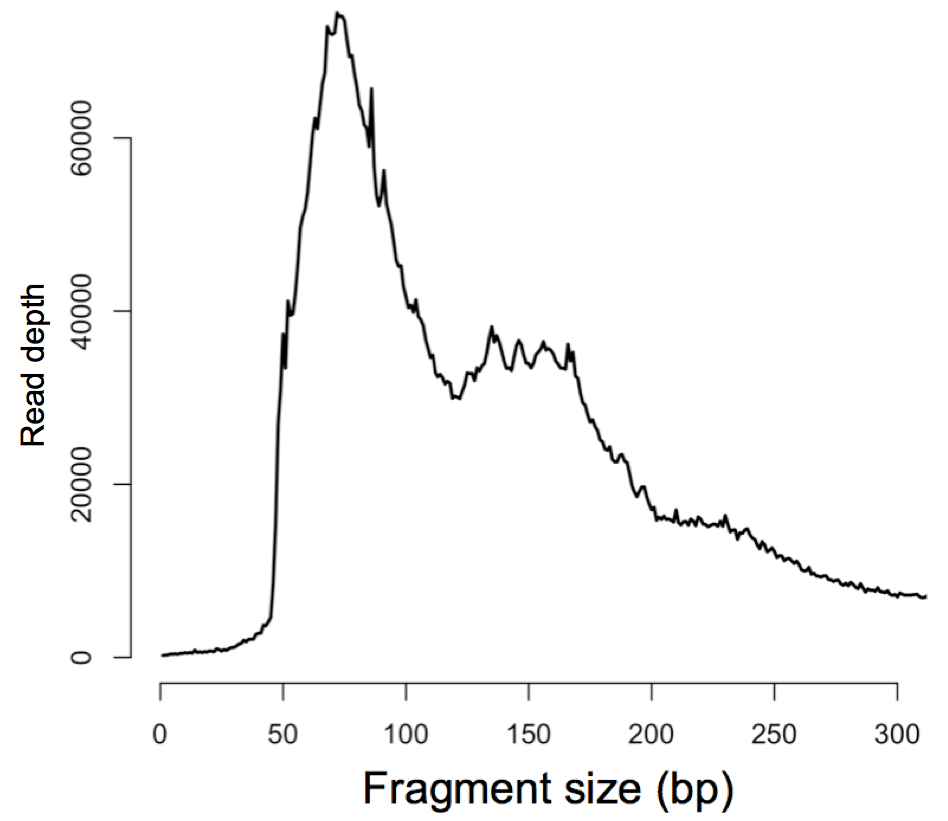

Supplement: S7 Fig — (TIF) [file pgen.1009714.s007.tif]

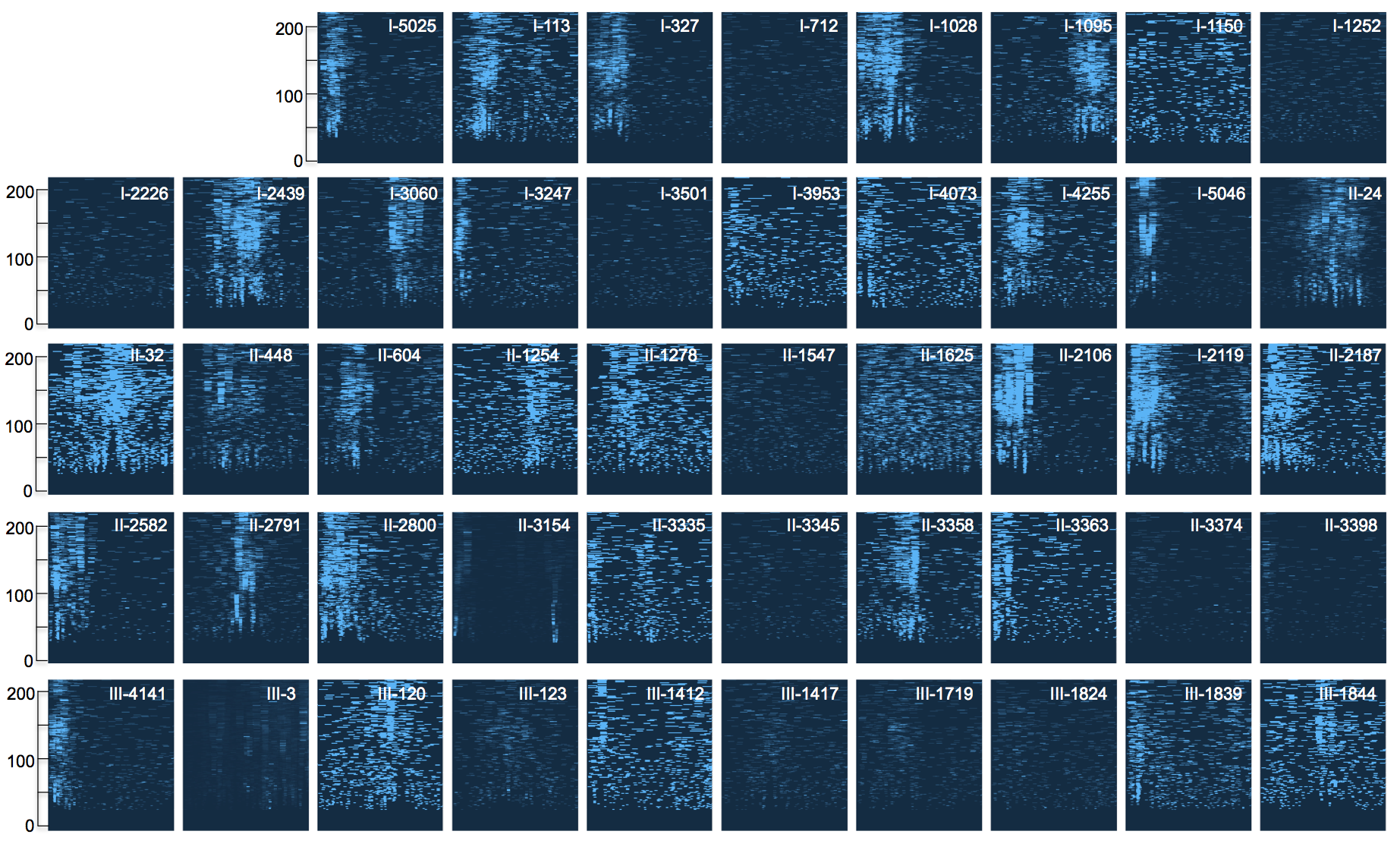

Supplement: S8 Fig — Each figure shows a 3 kb span on the X axis. Fragment sizes range from 0 to 200 bp on the Y axis. Plots are centered on midpoints of origins, as listed in OriDB. Read depths are listed as described in S4 Fig: chrIII_120001 1 (10); chrIII_123404 6 (6); chrIII_1411610 1 (8); chrIII_1417150 6 (6); chrIII_1719145 6 (6); chrIII_1823925 6 (6); chrIII_1838655 2 (20); chrIII_1844225 1 (10); chrIII_3479 259 (2074); chrII_1254200 1 (10); chrII_1278060 1 (9); chrII_1547425 5 (5); chrII_1625285 3 (29); chrII_2105805 3 (30); chrII_2118500 3 (25); chrII_2187470 1 (15); chrII_24041 20 (160); chrII_2582435 3 (31); chrII_2790759 6 (52); chrII_2800137 2 (21); chrII_3154425 35 (281); chrII_31591 1 (14); chrII_3334855 1 (9); chrII_3344900 5 (5); chrII_3357660 2 (18); chrII_3362660 1 (10); chrII_3374145 6 (6); chrII_3398460 6 (6); chrII_4141200 5 (44); chrII_448341 4 (32); chrII_603722 3 (26); chrI_1028000 3 (24); chrI_1094953 3 (24); chrI_113108 2 (16); chrI_1149805 1 (8); chrI_1252175 5 (5); chrI_2225555 4 (4); chrI_2438581 12 (103); chrI_3059510 4 (33); chrI_3247323 3 (29); chrI_327080 5 (46); chrI_3500690 4 (4); chrI_3954285 1 (9); chrI_4072925 1 (9); chrI_4254830 2 (19); chrI_5025445 4 (33); chrI_5046440 6 (55); chrI_712465 6 (6). (TIF) [file pgen.1009714.s008.tif]

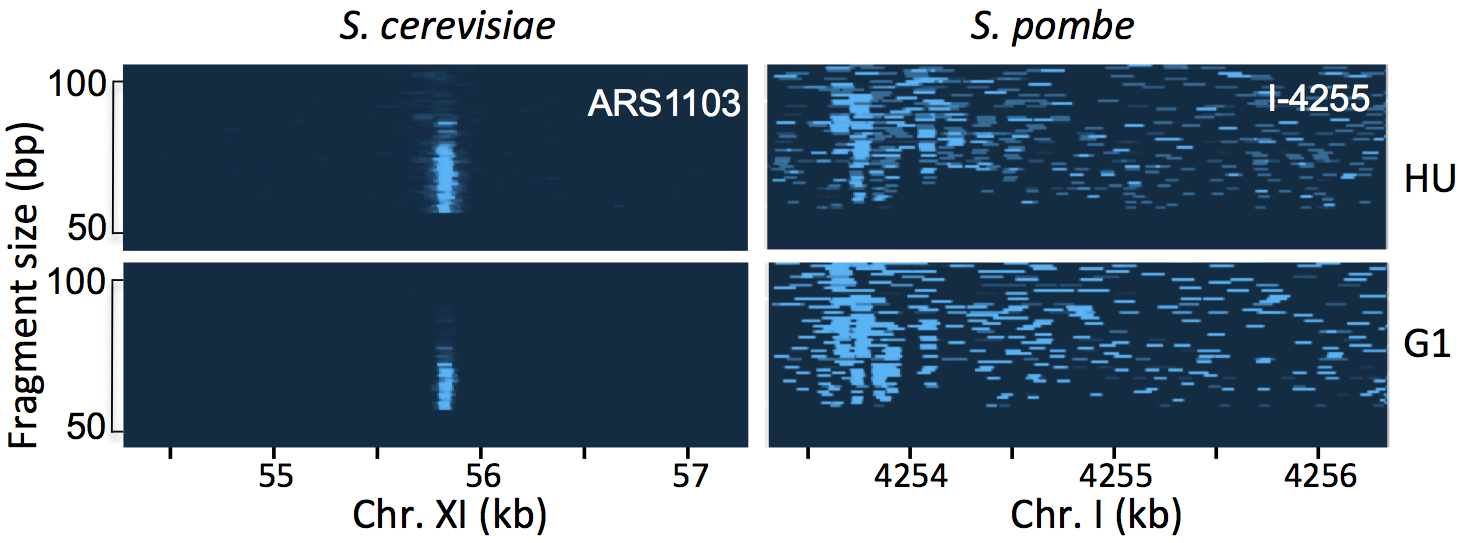

Supplement: S9 Fig — S. cerevisiae ARS1103 is a highly active origin that replicates early in S-phase. (TIF) [file pgen.1009714.s009.tif]

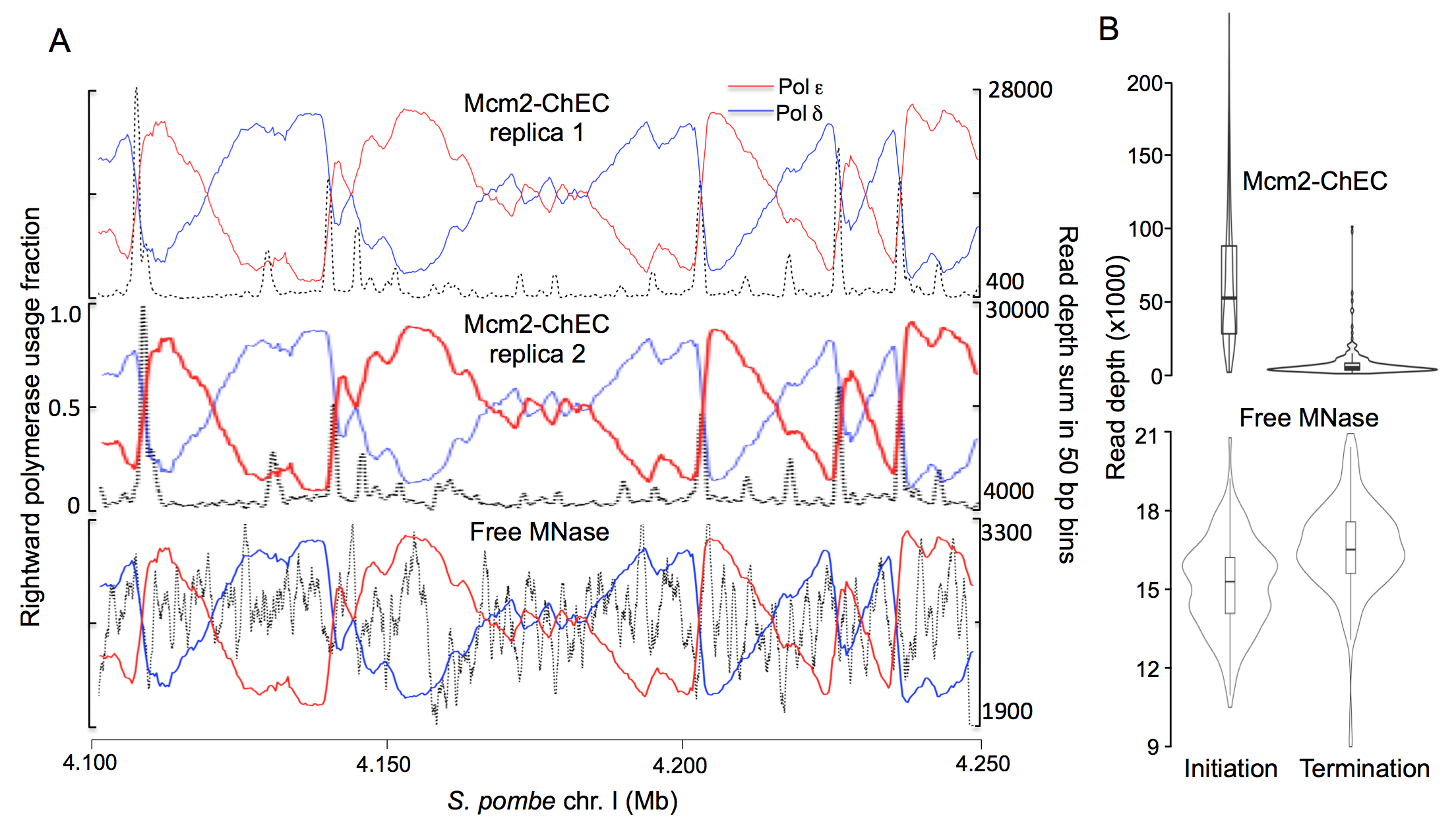

Supplement: S10 Fig — A. Pu-seq (red and blue for pol epsilon and pol delta, respecively) and Mcm2-ChEC (black) signal over 150 kb stretch of chrII. Replicate Mcm2-ChEC measurements (top two rows) show similar colocalization with sites of replication initiation, but not with sites of replication termination, as described in the text. Free MNase (bottom row) does not show colocalization with replication initiation or termination sites. All fragment sizes are included in the analysis. B. Genome-wide quantitation of data from A expressed as violin plots. (TIF) [file pgen.1009714.s010.tif]

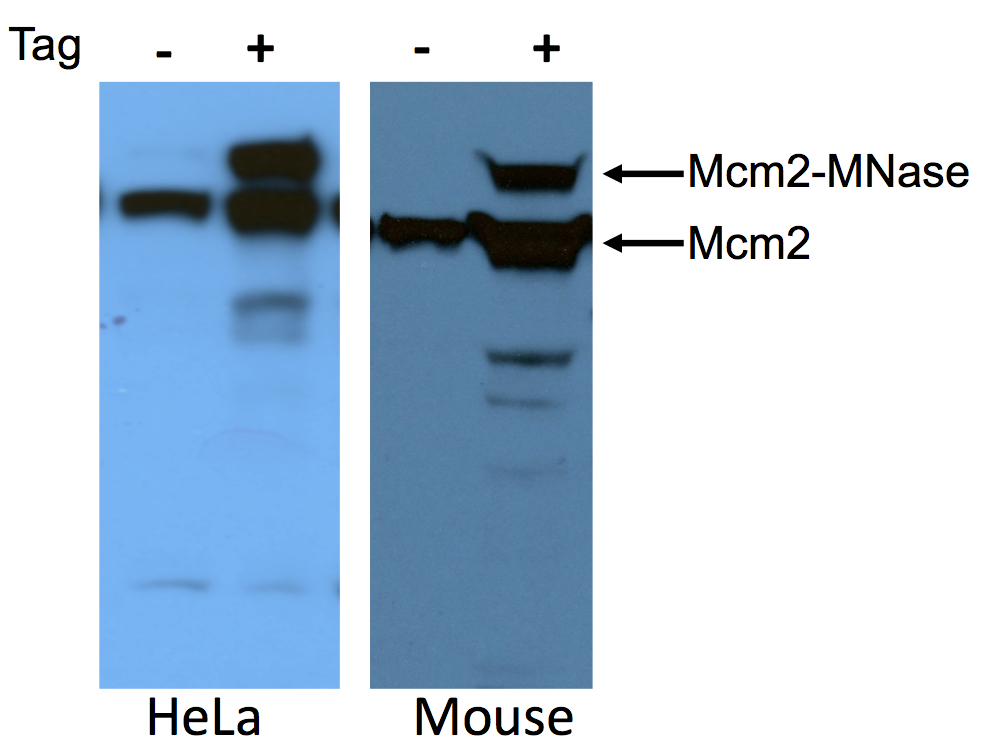

Supplement: S11 Fig — The relative abundance of tagged vs untagged bands for human and mouse cells was 0.4- and 0.25- fold, respectively. (TIF) [file pgen.1009714.s011.tif]

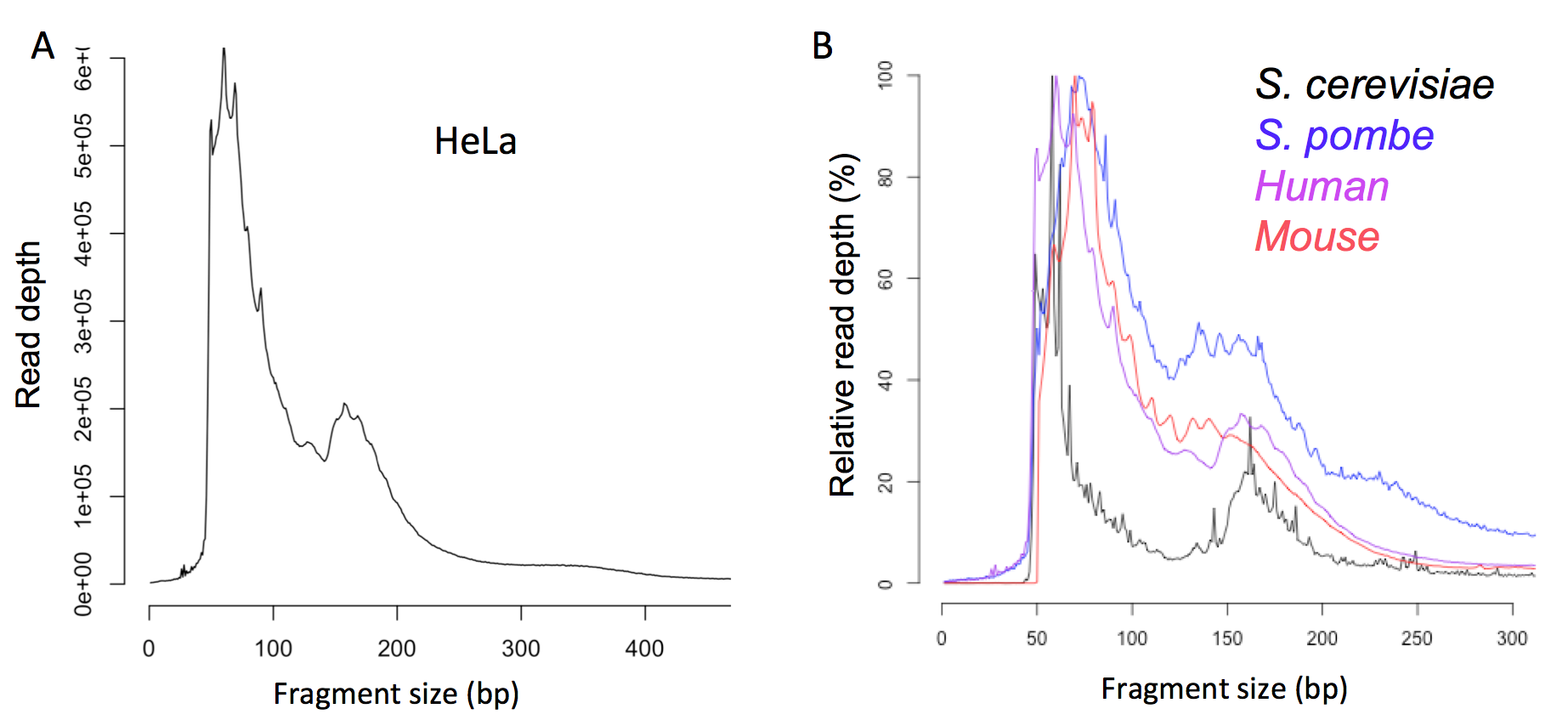

Supplement: S12 Fig — A. Size distribution of Mcm2-ChEC library fragments in HeLa cells. B. Size distribution of Mcm2-ChEC library fragments in budding yeas (black), fission yeast (blue), mouse (red) and human (purple). (TIF) [file pgen.1009714.s012.tif]

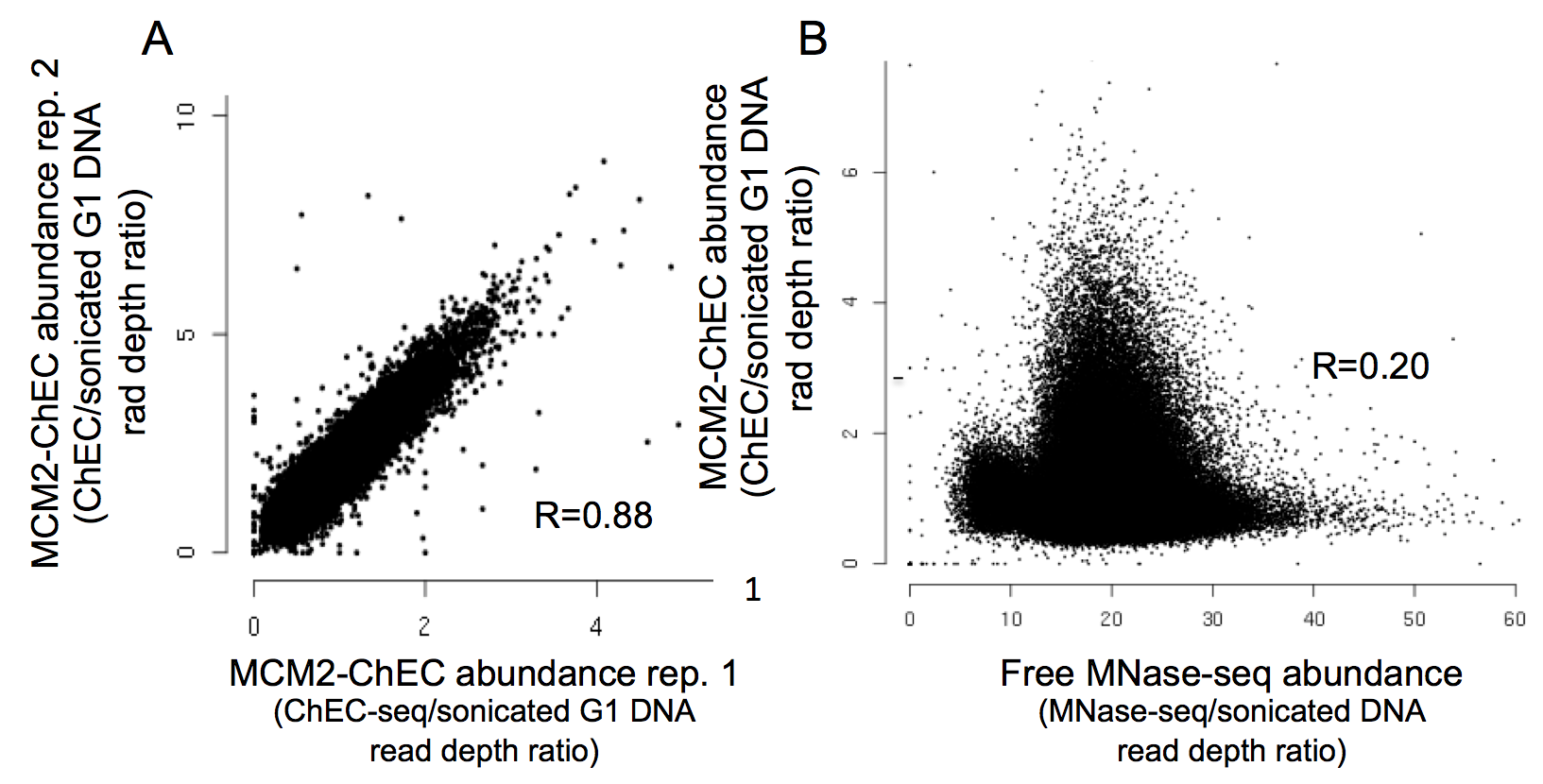

Supplement: S13 Fig — All fragment sizes are included in the analysis. (TIF) [file pgen.1009714.s013.tif]

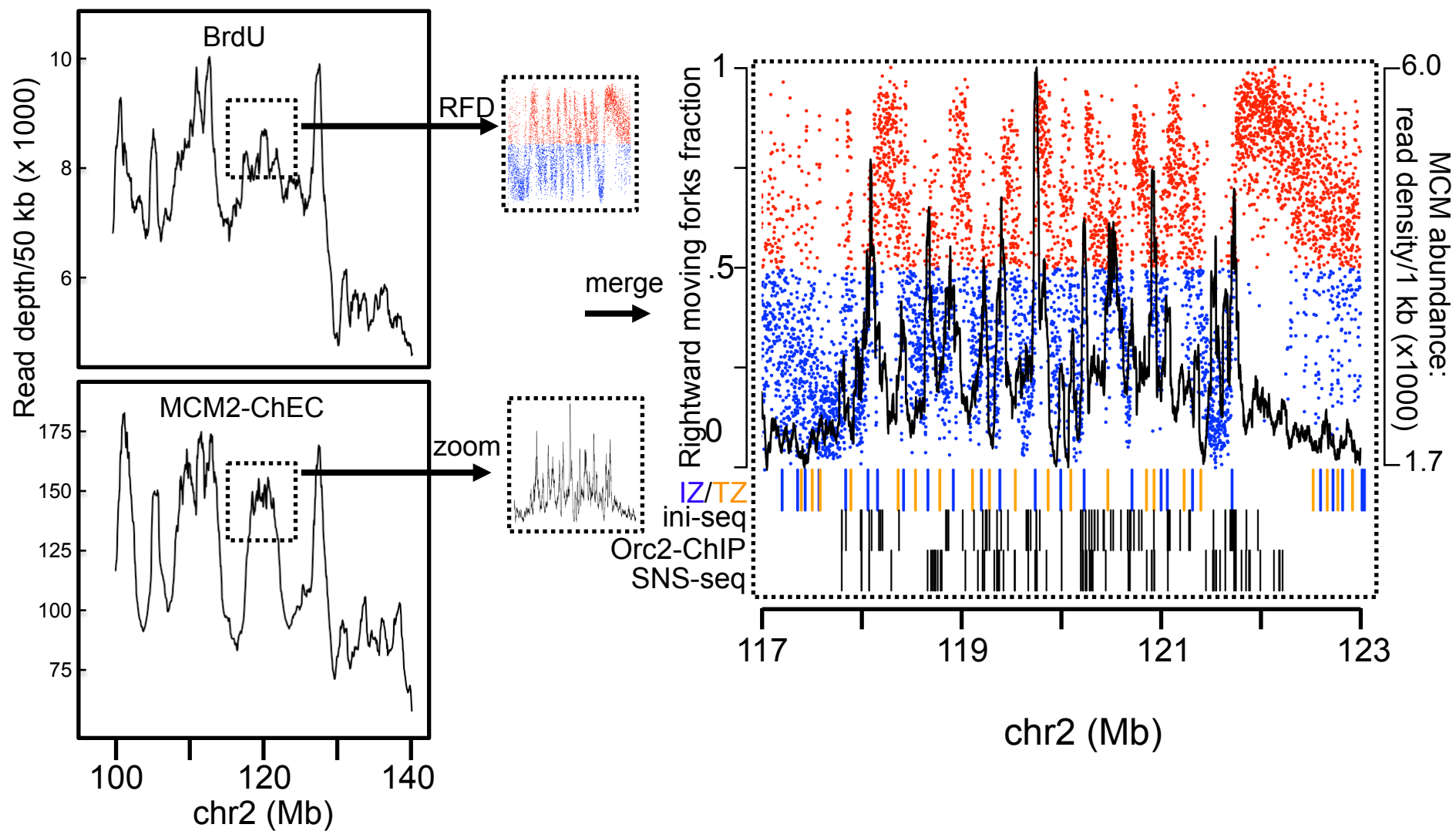

Panel A

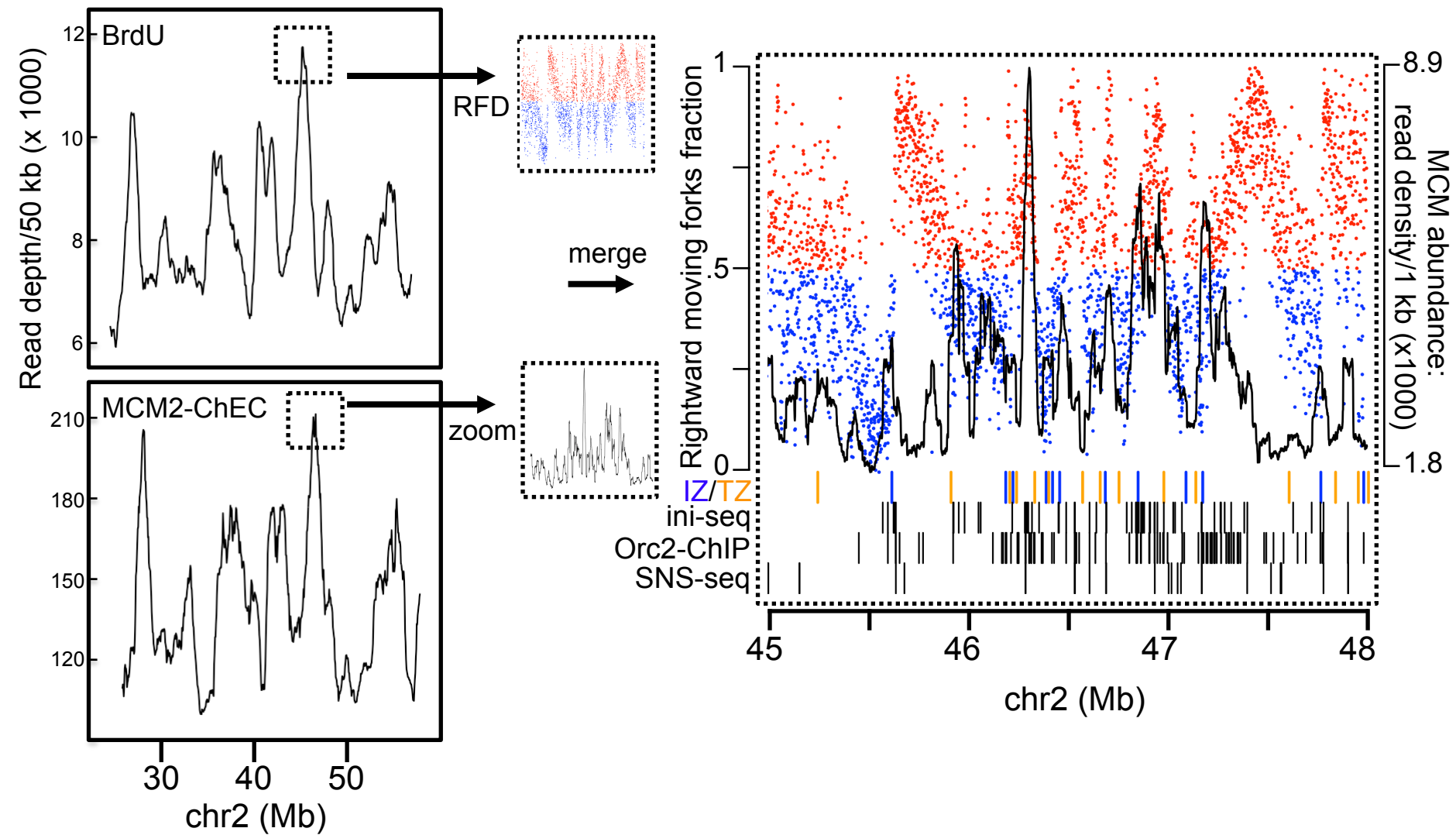

Panel B

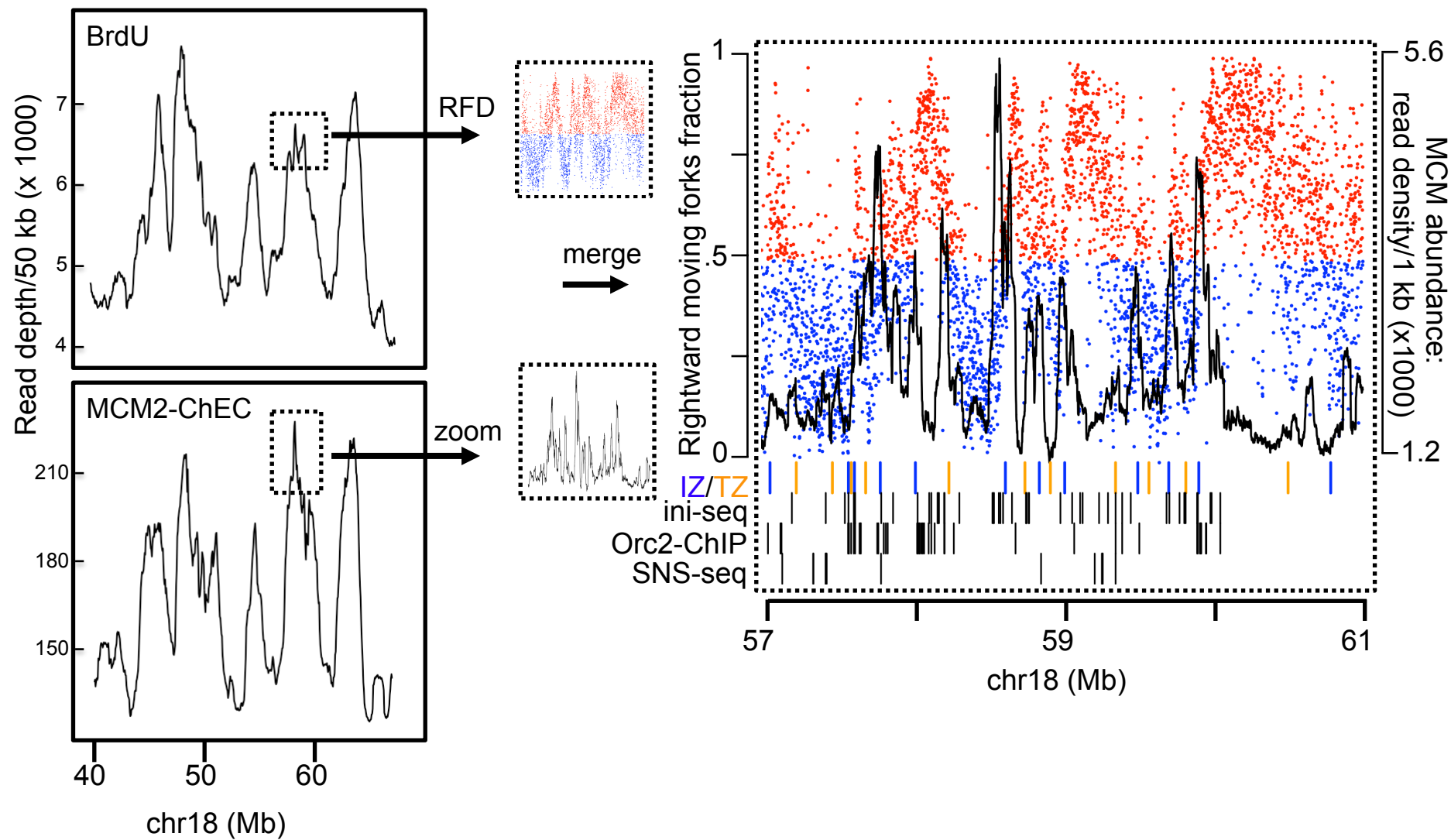

Panel C

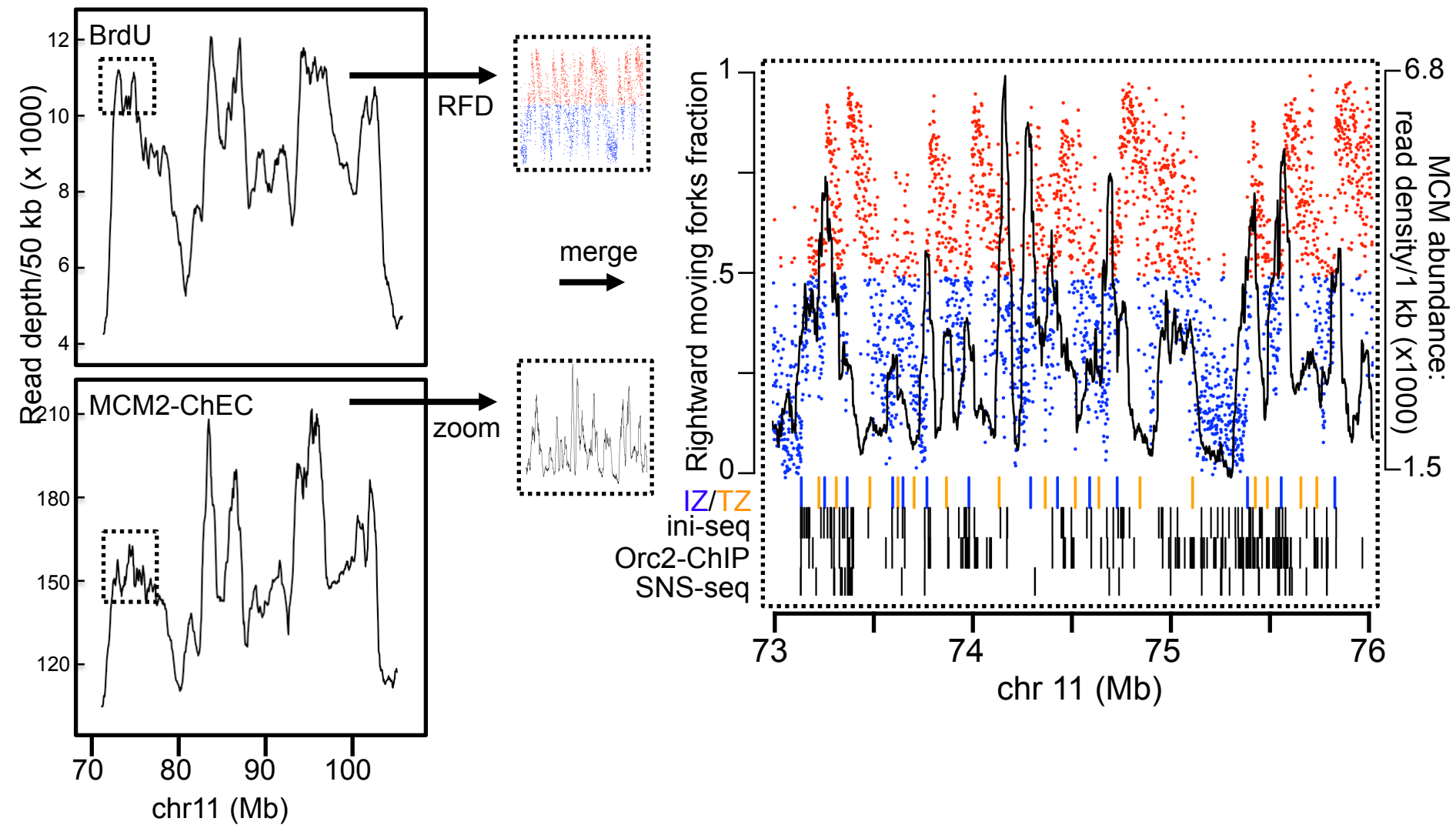

Panel D

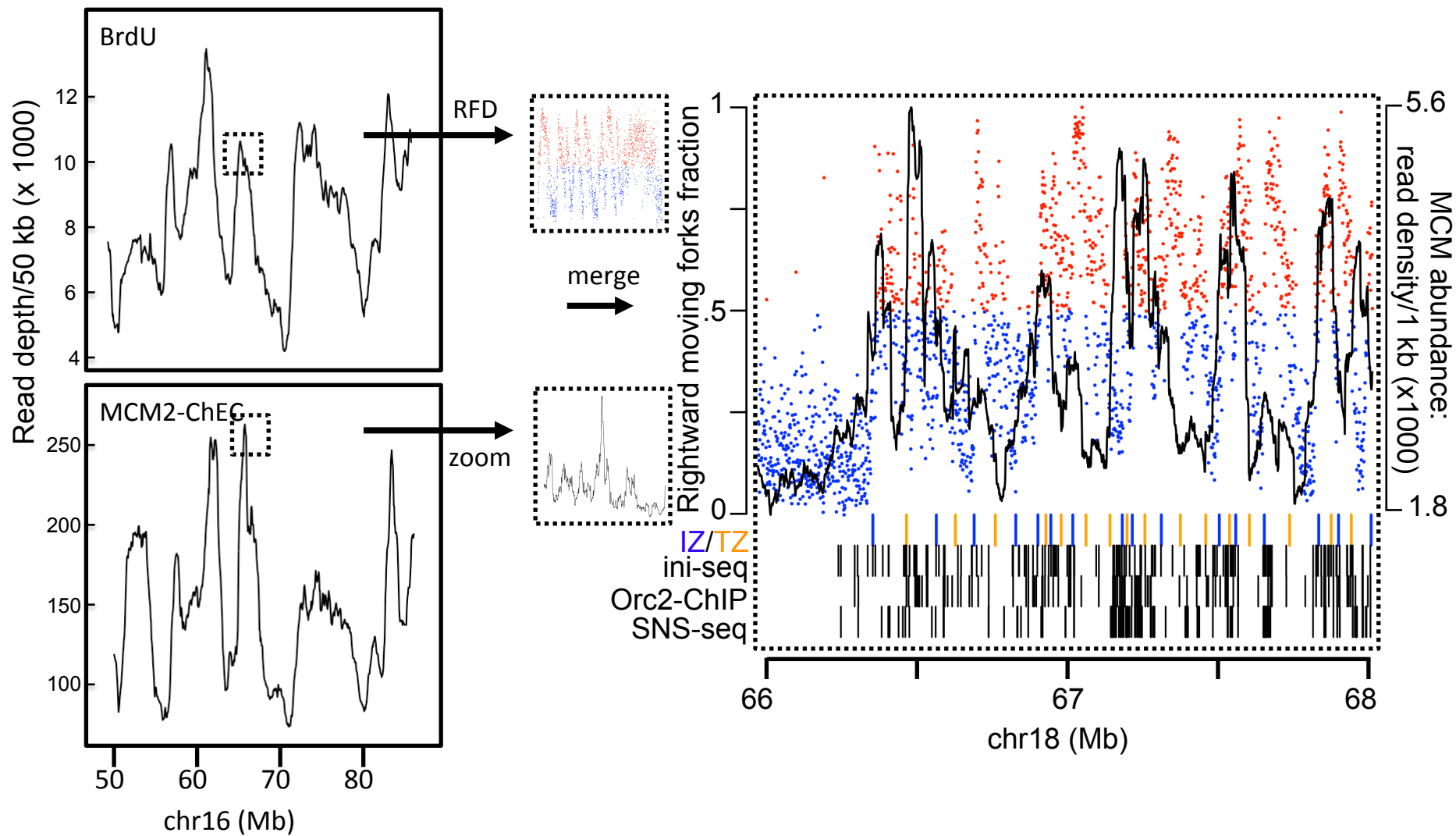

Panel E

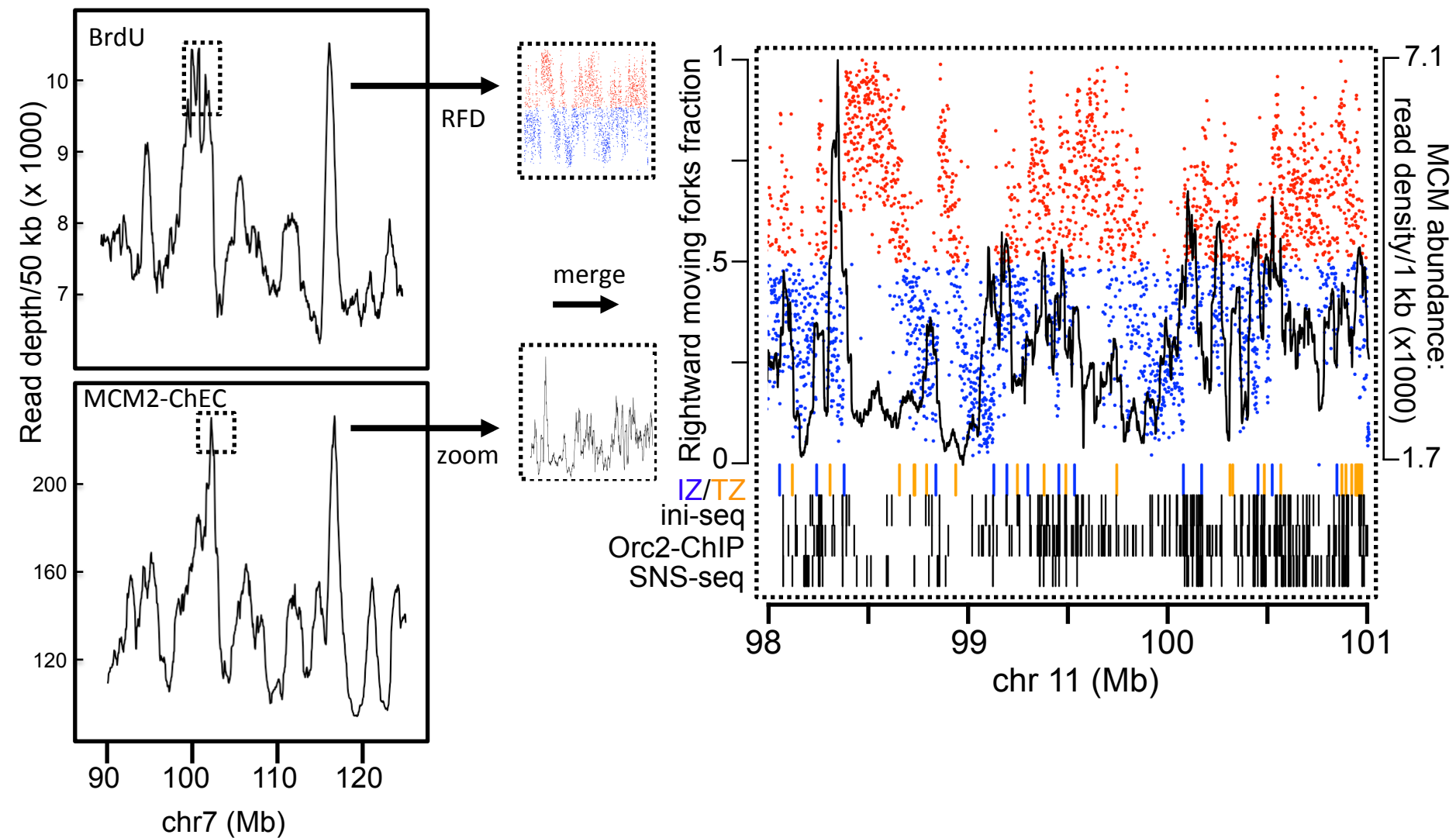

Panel F

Supplement: S14 Fig — A-F. Six examples of replication fork direction assays showing fraction of forks that synthesize the Watson strand that are moving rightward (red and blue dots used to indicate direction of movement of majority of forks) juxtaposed with MCM binding (black) as in Fig 7A. All Mcm2-ChEC fragment sizes are included in the analysis. (PDF) [file pgen.1009714.s014.PDF]

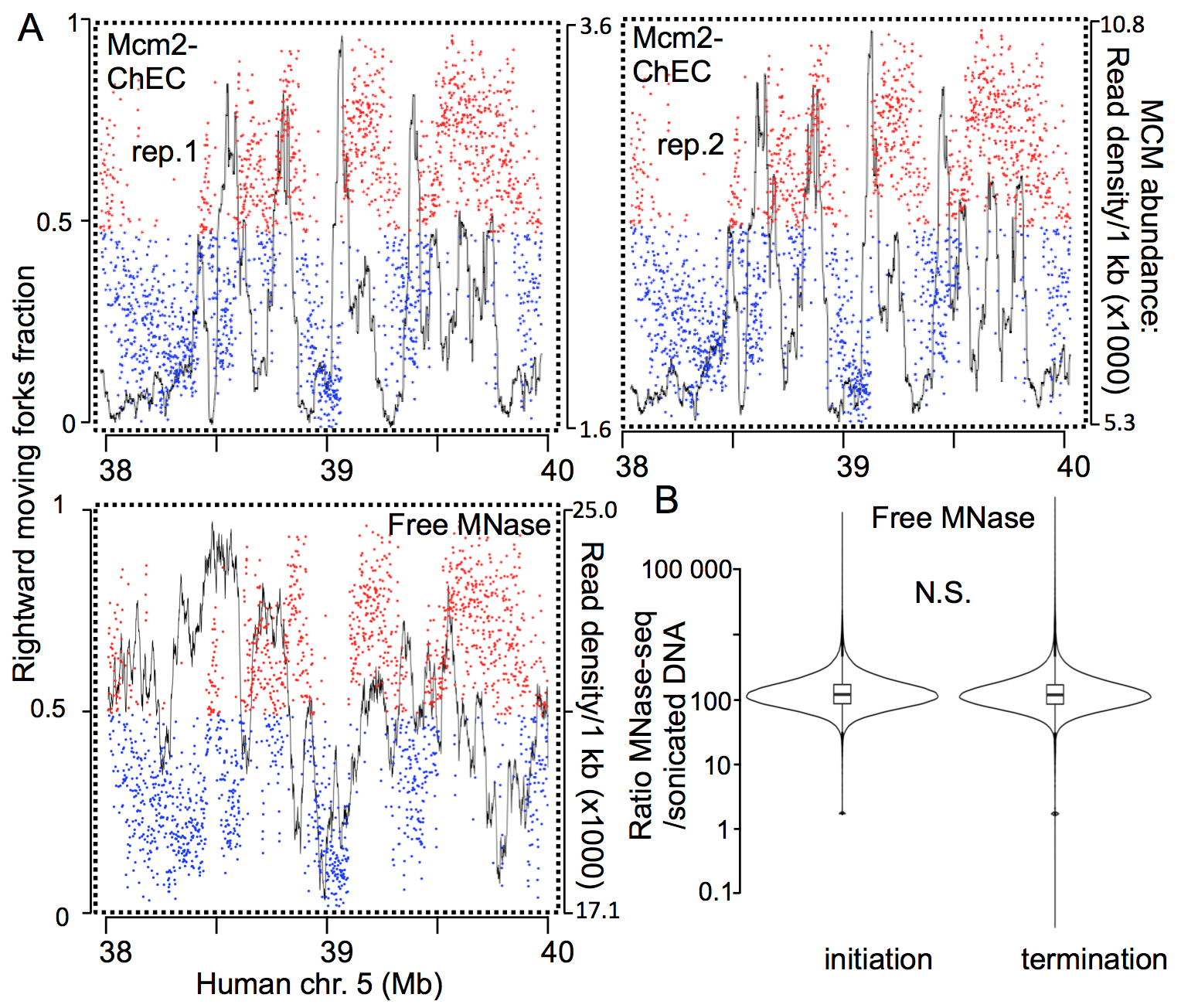

Supplement: S15 Fig — A. Dots represent the fraction of forks involved in synthesis of the Watson strand that are moving to the right, as described in Fig 7. Dots are red when >50% of forks are moving rightward, and blue otherwise. Replicate measurements of Mcm2-ChEC (black lines in two top panels) show comparable colocalization with replication initiation sites, but free MNase (black line in bottom left panel) does not. See text and Fig 7 for details. B. Quantitation of free MNase results from A, illustrated as a violin plot. "NS" indicates that the difference between levels of free MNase/G1 DNA ratio signal at replication initiation and termination sites are not significantly different. (TIF) [file pgen.1009714.s015.tif]

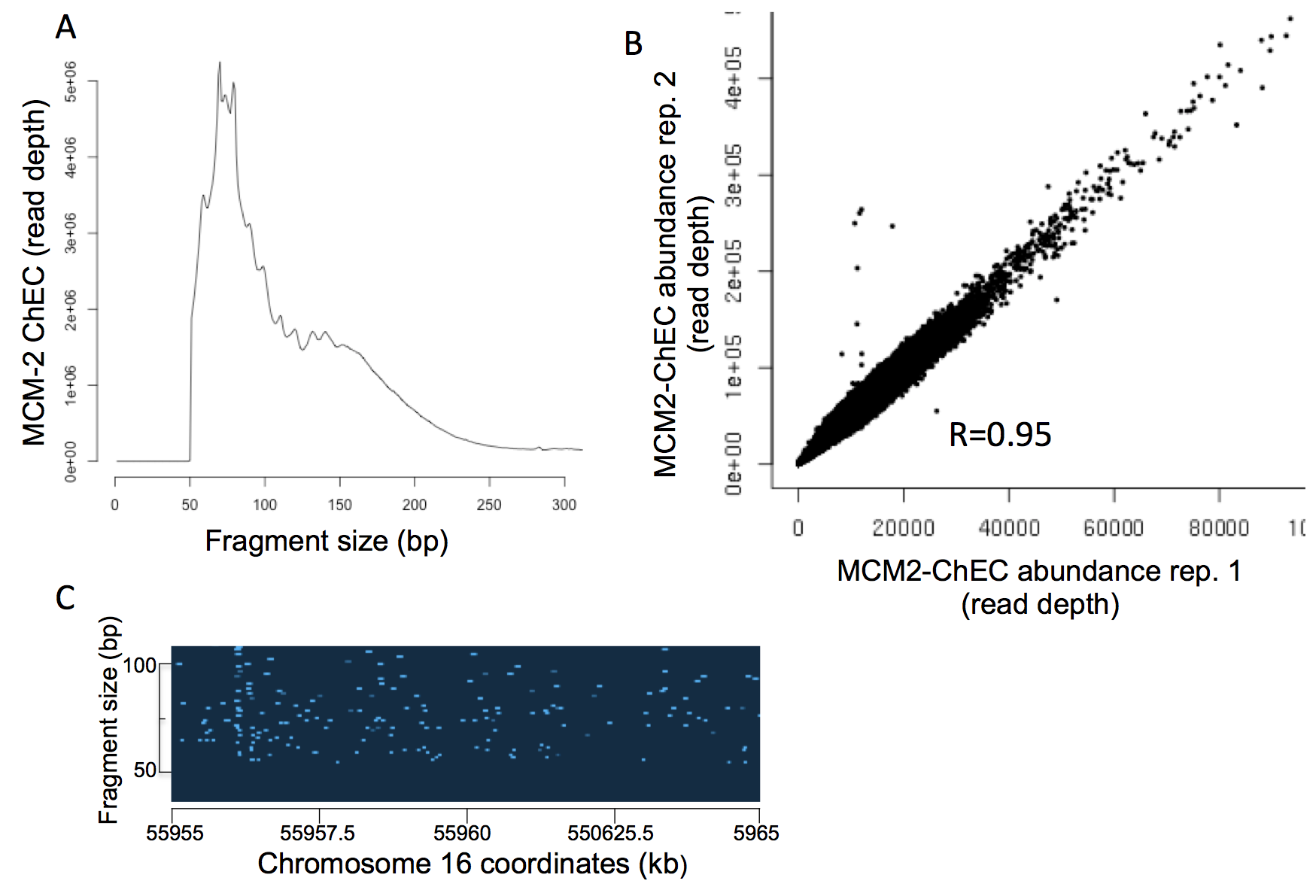

Supplement: S16 Fig — A. Size distribution of Mcm2-ChEC library. B. Replicate measurements of Mcm2-ChEC signal (10 kb bins) are highly correlated (R = 0.96). (TIF) [file pgen.1009714.s016.tif]

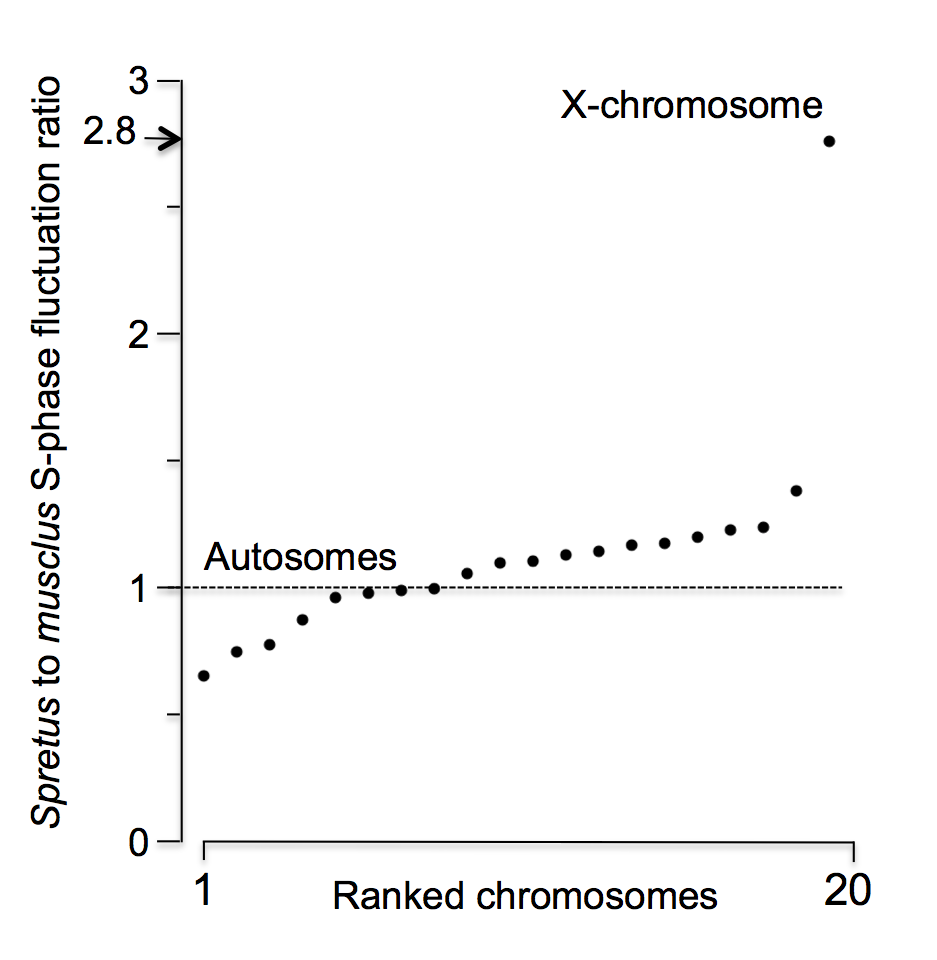

Supplement: S17 Fig — Absolute Deviations (MAD) of read depths in S-phase along M. spretus and M. musculus chromosomes were used to measure replication fluctuations for each chromosome. The ratio of fluctuations between two chromosomes was highest for the X chromosome (2.8), indicating that the difference in replication time between the two homologs is most extreme for this chromosome. Chromosomes are arranged in order of fluctuation ratios. (TIF) [file pgen.1009714.s017.tif]

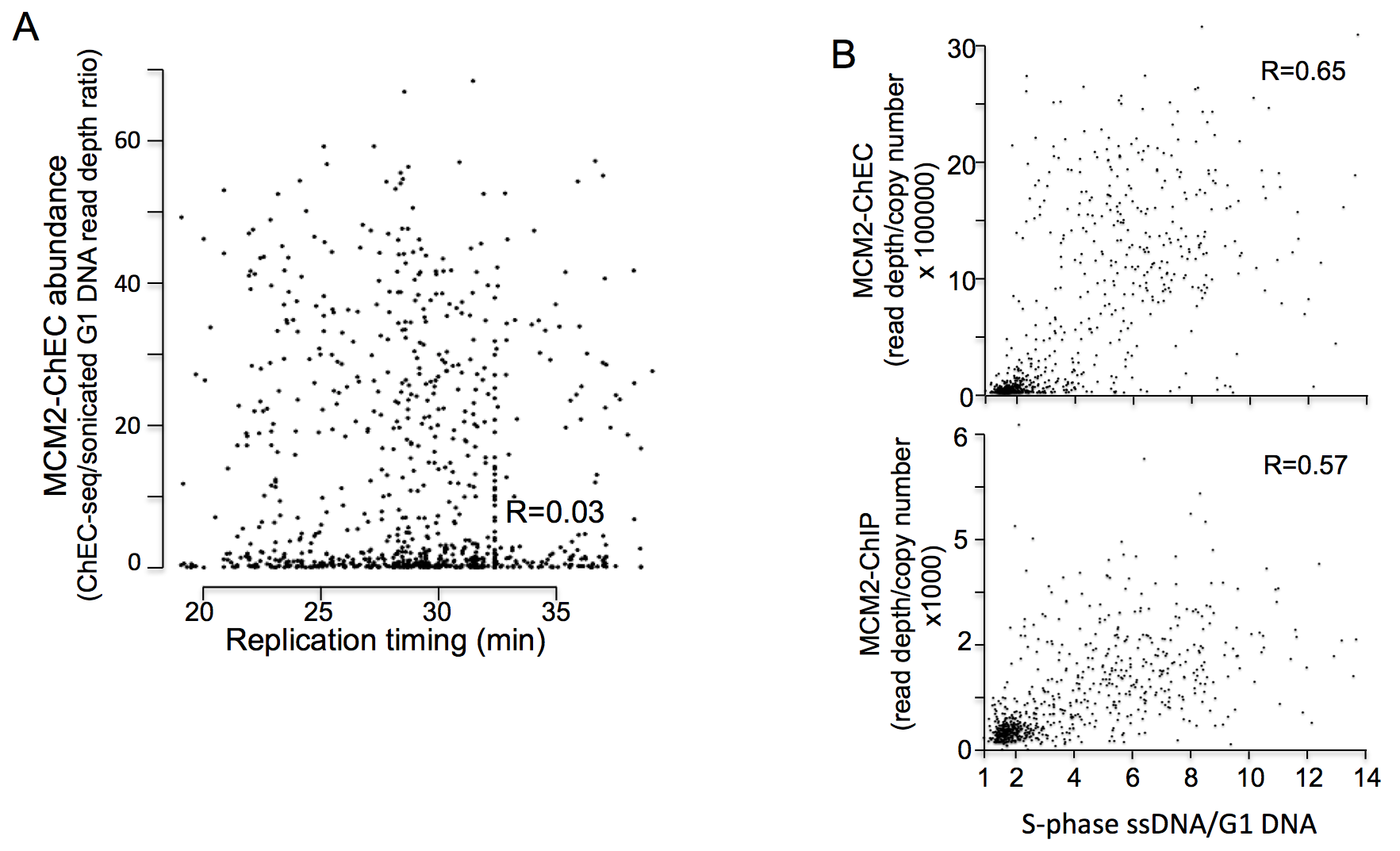

Supplement: S18 Fig — A. Mcm2-ChEC signal at 829 S. cerevisiae origins listed in OriDB is not correlated with replication timing. B. Replication activity at OriDB origins excluding repetitive regions, as measured by single-stranded DNA [46] is correlated with both Mcm2-ChEC (R = 0.65) and Mcm-ChIP (R = 0.57) [45] signal. All Mcm2-ChEC fragment sizes are included in the analysis. (TIF) [file pgen.1009714.s018.tif]

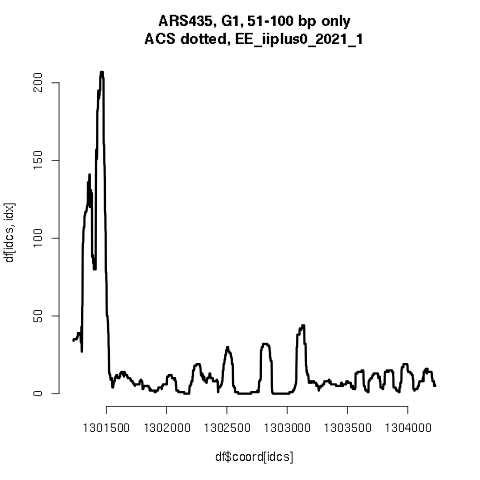

Supplement: S7 Source Files — (ZIP) [file pgen.1009714.s029.zip › source_files_for_S5Figure_to_S12Figure/S5_Figure/ARS435_chrIV_1302728_G1_51to100bp_lwd3_050921_1.tiff]

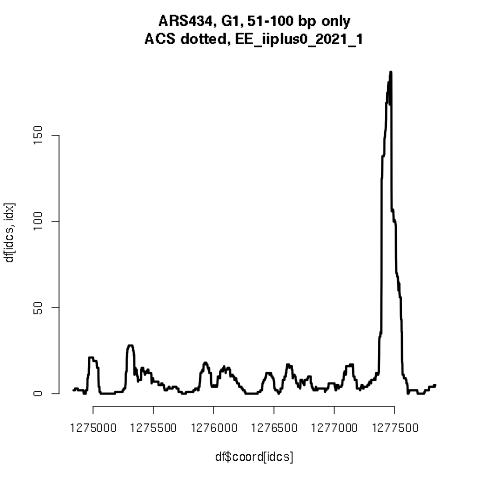

Supplement: S7 Source Files — (ZIP) [file pgen.1009714.s029.zip › source_files_for_S5Figure_to_S12Figure/S5_Figure/ARS434_chrIV_1276338_G1_51to100bp_lwd3_050921_1.tiff]

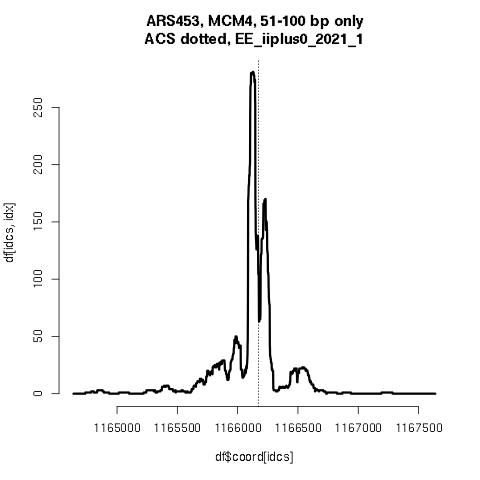

Supplement: S7 Source Files — (ZIP) [file pgen.1009714.s029.zip › source_files_for_S5Figure_to_S12Figure/S5_Figure/ARS453_chrIV_1166139_MCM4_51to100bp_lwd3_050921_1.tiff]

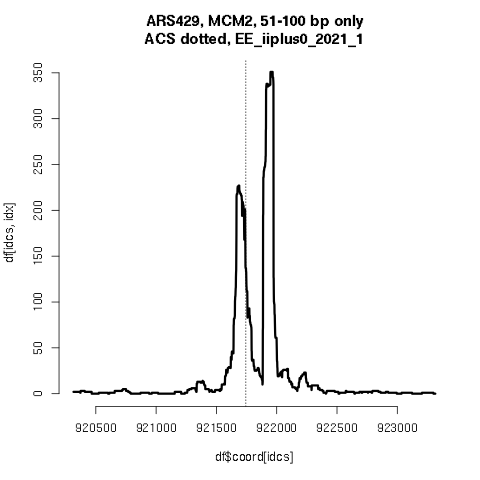

Supplement: S7 Source Files — (ZIP) [file pgen.1009714.s029.zip › source_files_for_S5Figure_to_S12Figure/S5_Figure/ARS429_chrIV_921815_MCM2_51to100bp_lwd3_050921_1.tiff]

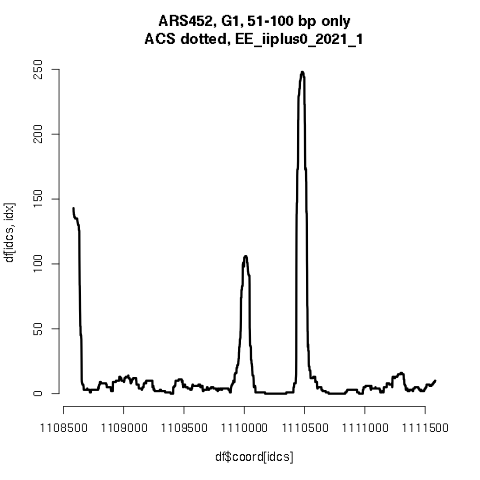

Supplement: S7 Source Files — (ZIP) [file pgen.1009714.s029.zip › source_files_for_S5Figure_to_S12Figure/S5_Figure/ARS452_chrIV_1110084_G1_51to100bp_lwd3_050921_1.tiff]

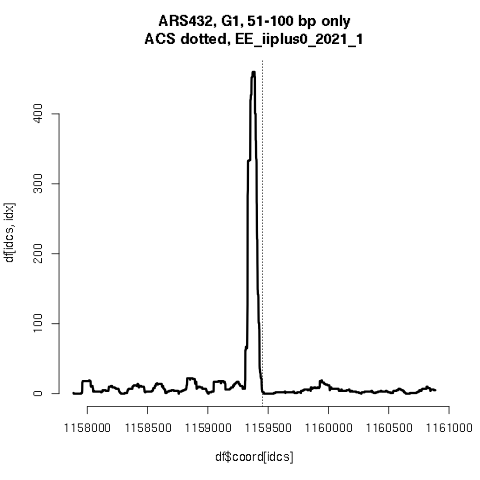

Supplement: S7 Source Files — (ZIP) [file pgen.1009714.s029.zip › source_files_for_S5Figure_to_S12Figure/S5_Figure/ARS432_chrIV_1159386_G1_51to100bp_lwd3_050921_1.tiff]

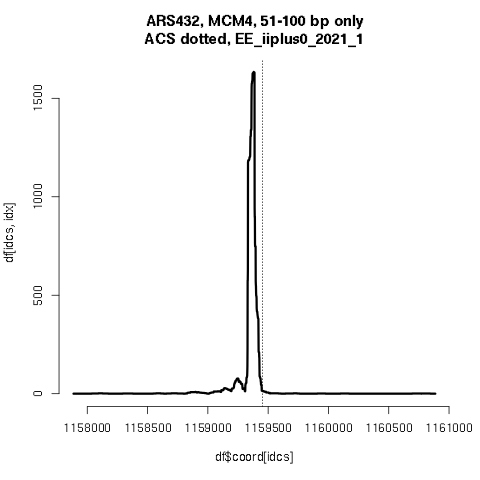

Supplement: S7 Source Files — (ZIP) [file pgen.1009714.s029.zip › source_files_for_S5Figure_to_S12Figure/S5_Figure/ARS432_chrIV_1159386_MCM4_51to100bp_lwd3_050921_1.tiff]

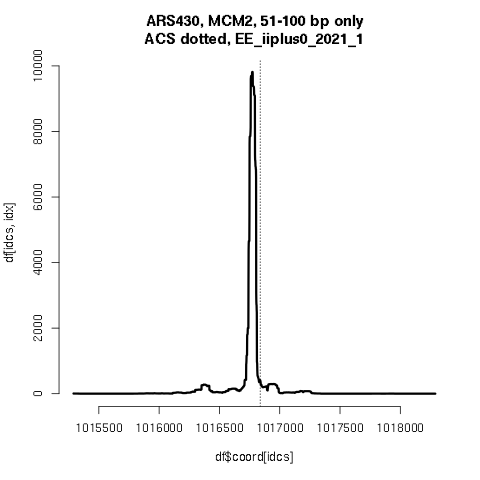

Supplement: S7 Source Files — (ZIP) [file pgen.1009714.s029.zip › source_files_for_S5Figure_to_S12Figure/S5_Figure/ARS430_chrIV_1016790_MCM2_51to100bp_lwd3_050921_1.tiff]

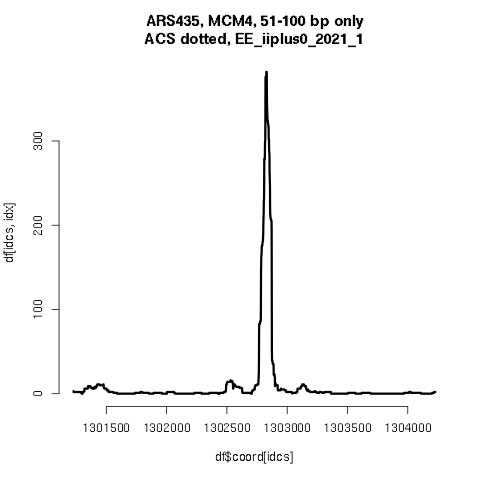

Supplement: S7 Source Files — (ZIP) [file pgen.1009714.s029.zip › source_files_for_S5Figure_to_S12Figure/S5_Figure/ARS435_chrIV_1302728_MCM4_51to100bp_lwd3_050921_1.tiff]

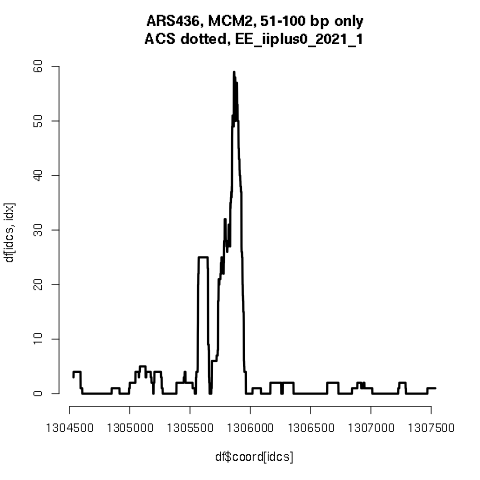

Supplement: S7 Source Files — (ZIP) [file pgen.1009714.s029.zip › source_files_for_S5Figure_to_S12Figure/S5_Figure/ARS436_chrIV_1306034_MCM2_51to100bp_lwd3_050921_1.tiff]

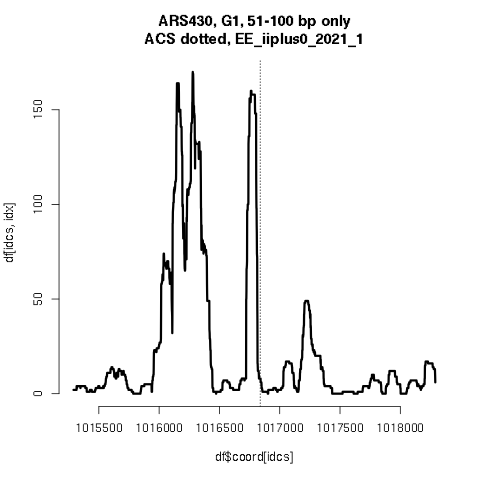

Supplement: S7 Source Files — (ZIP) [file pgen.1009714.s029.zip › source_files_for_S5Figure_to_S12Figure/S5_Figure/ARS430_chrIV_1016790_G1_51to100bp_lwd3_050921_1.tiff]

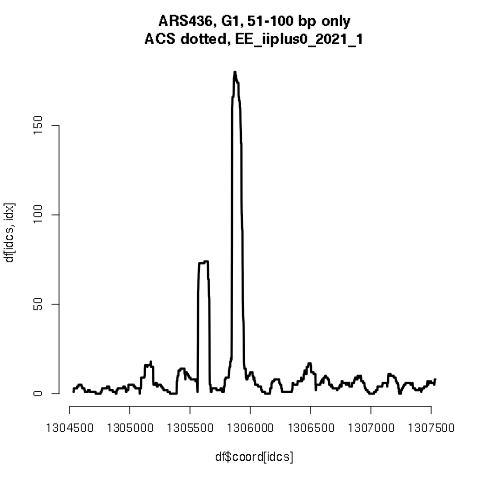

Supplement: S7 Source Files — (ZIP) [file pgen.1009714.s029.zip › source_files_for_S5Figure_to_S12Figure/S5_Figure/ARS436_chrIV_1306034_G1_51to100bp_lwd3_050921_1.tiff]

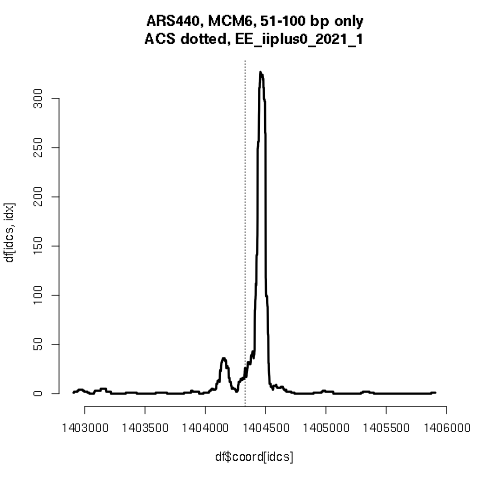

Supplement: S7 Source Files — (ZIP) [file pgen.1009714.s029.zip › source_files_for_S5Figure_to_S12Figure/S5_Figure/ARS440_chrIV_1404407_MCM6_51to100bp_lwd3_050921_1.tiff]

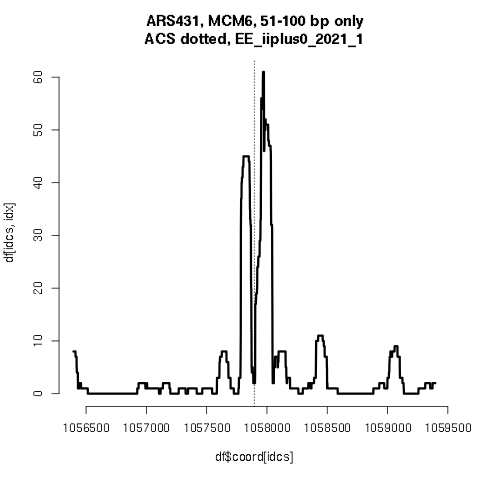

Supplement: S7 Source Files — (ZIP) [file pgen.1009714.s029.zip › source_files_for_S5Figure_to_S12Figure/S5_Figure/ARS431_chrIV_1057896_MCM6_51to100bp_lwd3_050921_1.tiff]

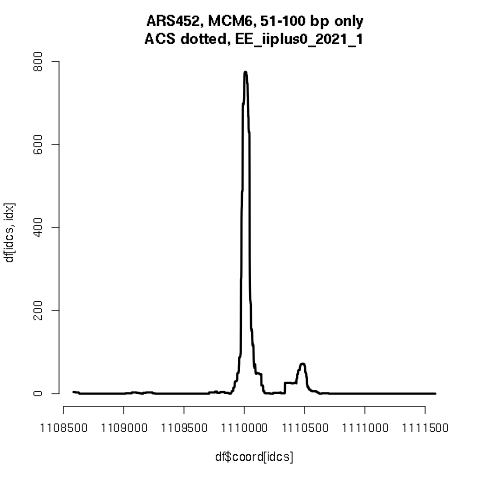

Supplement: S7 Source Files — (ZIP) [file pgen.1009714.s029.zip › source_files_for_S5Figure_to_S12Figure/S5_Figure/ARS452_chrIV_1110084_MCM6_51to100bp_lwd3_050921_1.tiff]

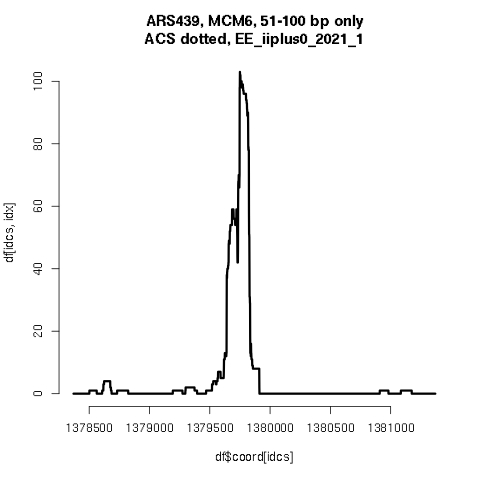

Supplement: S7 Source Files — (ZIP) [file pgen.1009714.s029.zip › source_files_for_S5Figure_to_S12Figure/S5_Figure/ARS439_chrIV_1379870_MCM6_51to100bp_lwd3_050921_1.tiff]

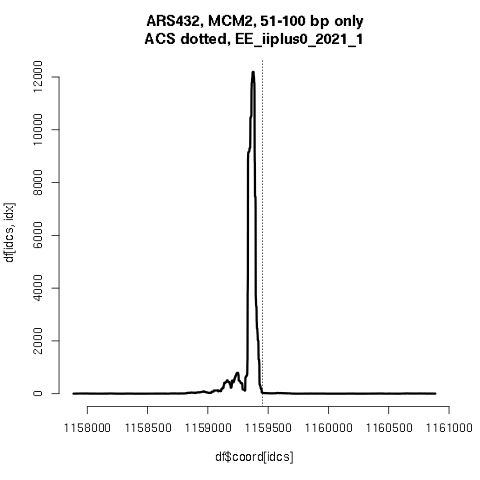

Supplement: S7 Source Files — (ZIP) [file pgen.1009714.s029.zip › source_files_for_S5Figure_to_S12Figure/S5_Figure/ARS432_chrIV_1159386_MCM2_51to100bp_lwd3_050921_1.tiff]

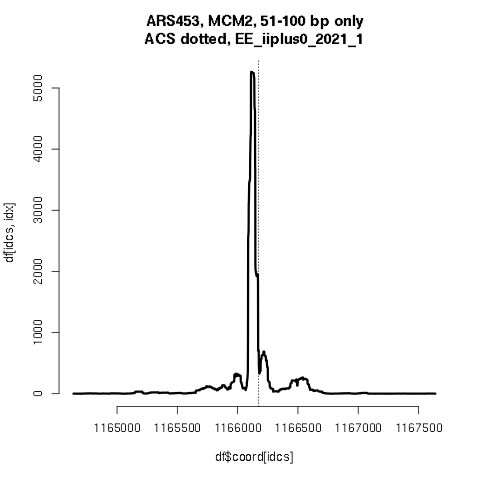

Supplement: S7 Source Files — (ZIP) [file pgen.1009714.s029.zip › source_files_for_S5Figure_to_S12Figure/S5_Figure/ARS453_chrIV_1166139_MCM2_51to100bp_lwd3_050921_1.tiff]

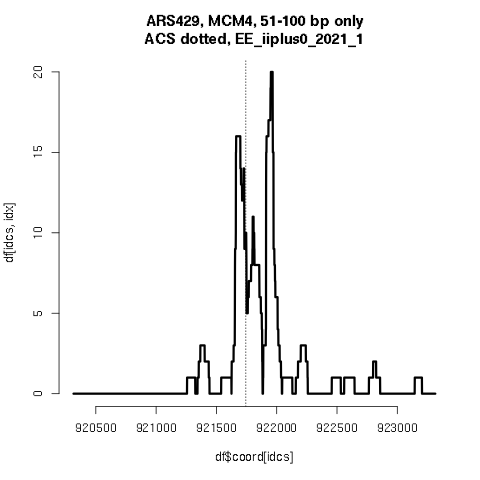

Supplement: S7 Source Files — (ZIP) [file pgen.1009714.s029.zip › source_files_for_S5Figure_to_S12Figure/S5_Figure/ARS429_chrIV_921815_MCM4_51to100bp_lwd3_050921_1.tiff]

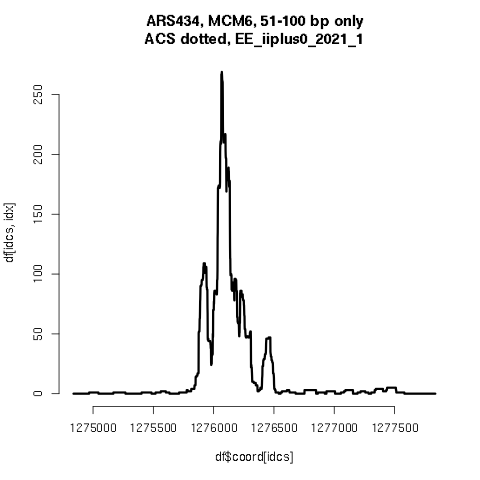

Supplement: S7 Source Files — (ZIP) [file pgen.1009714.s029.zip › source_files_for_S5Figure_to_S12Figure/S5_Figure/ARS434_chrIV_1276338_MCM6_51to100bp_lwd3_050921_1.tiff]

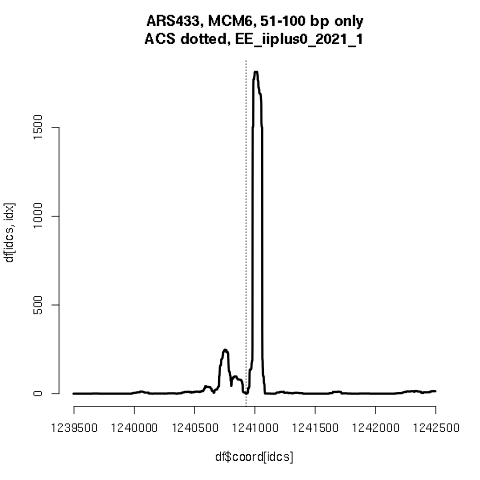

Supplement: S7 Source Files — (ZIP) [file pgen.1009714.s029.zip › source_files_for_S5Figure_to_S12Figure/S5_Figure/ARS433_chrIV_1240996_MCM6_51to100bp_lwd3_050921_1.tiff]

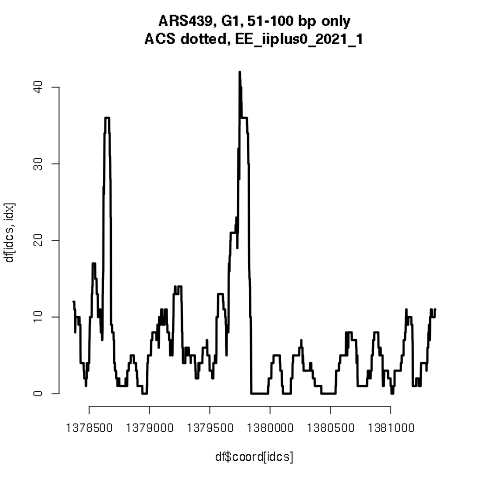

Supplement: S7 Source Files — (ZIP) [file pgen.1009714.s029.zip › source_files_for_S5Figure_to_S12Figure/S5_Figure/ARS439_chrIV_1379870_G1_51to100bp_lwd3_050921_1.tiff]

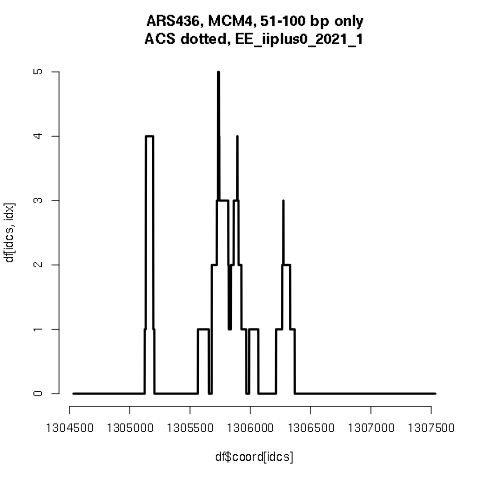

Supplement: S7 Source Files — (ZIP) [file pgen.1009714.s029.zip › source_files_for_S5Figure_to_S12Figure/S5_Figure/ARS436_chrIV_1306034_MCM4_51to100bp_lwd3_050921_1.tiff]

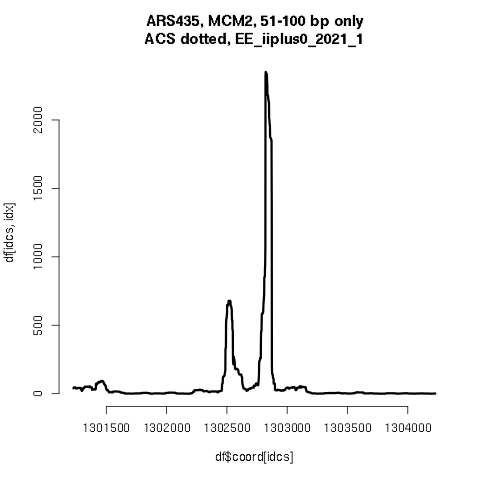

Supplement: S7 Source Files — (ZIP) [file pgen.1009714.s029.zip › source_files_for_S5Figure_to_S12Figure/S5_Figure/ARS435_chrIV_1302728_MCM2_51to100bp_lwd3_050921_1.tiff]

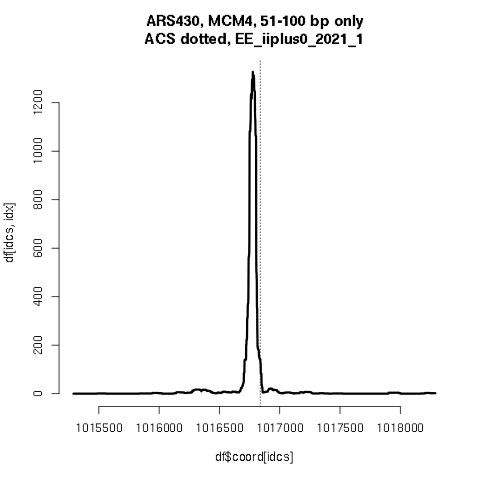

Supplement: S7 Source Files — (ZIP) [file pgen.1009714.s029.zip › source_files_for_S5Figure_to_S12Figure/S5_Figure/ARS430_chrIV_1016790_MCM4_51to100bp_lwd3_050921_1.tiff]

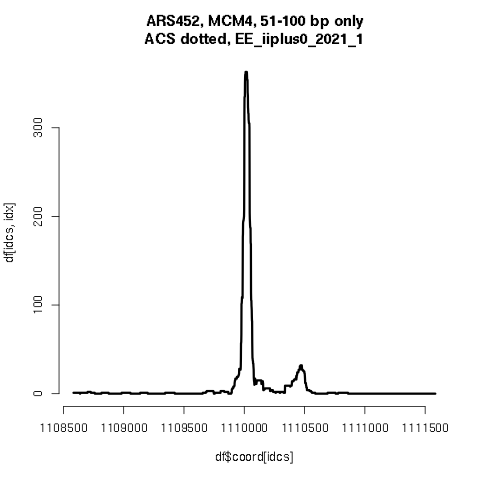

Supplement: S7 Source Files — (ZIP) [file pgen.1009714.s029.zip › source_files_for_S5Figure_to_S12Figure/S5_Figure/ARS452_chrIV_1110084_MCM4_51to100bp_lwd3_050921_1.tiff]

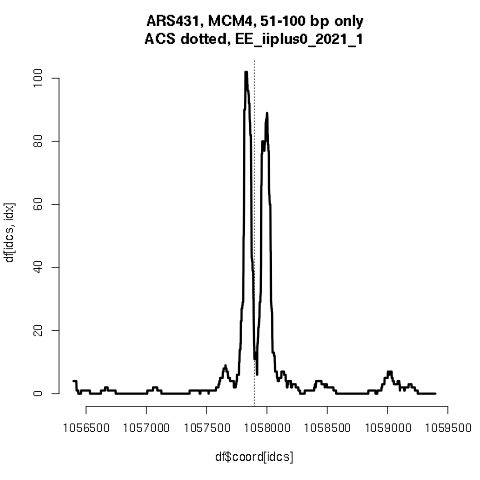

Supplement: S7 Source Files — (ZIP) [file pgen.1009714.s029.zip › source_files_for_S5Figure_to_S12Figure/S5_Figure/ARS431_chrIV_1057896_MCM4_51to100bp_lwd3_050921_1.tiff]

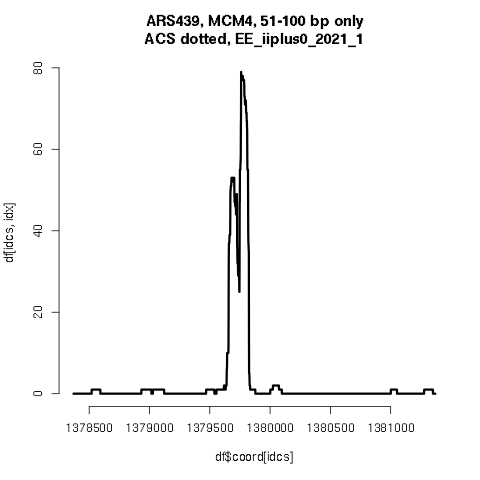

Supplement: S7 Source Files — (ZIP) [file pgen.1009714.s029.zip › source_files_for_S5Figure_to_S12Figure/S5_Figure/ARS439_chrIV_1379870_MCM4_51to100bp_lwd3_050921_1.tiff]

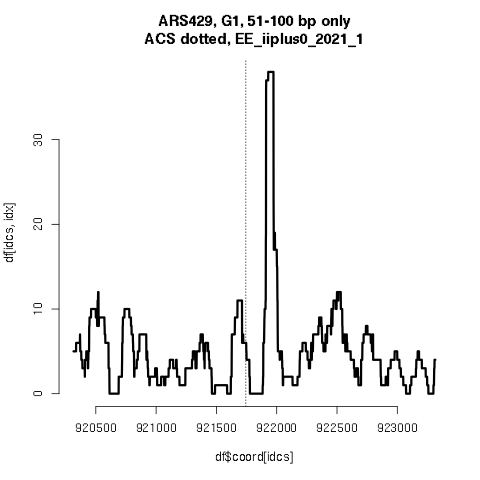

Supplement: S7 Source Files — (ZIP) [file pgen.1009714.s029.zip › source_files_for_S5Figure_to_S12Figure/S5_Figure/ARS429_chrIV_921815_G1_51to100bp_lwd3_050921_1.tiff]

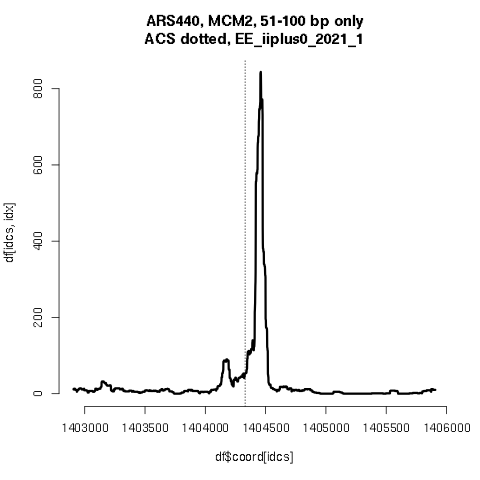

Supplement: S7 Source Files — (ZIP) [file pgen.1009714.s029.zip › source_files_for_S5Figure_to_S12Figure/S5_Figure/ARS440_chrIV_1404407_MCM2_51to100bp_lwd3_050921_1.tiff]

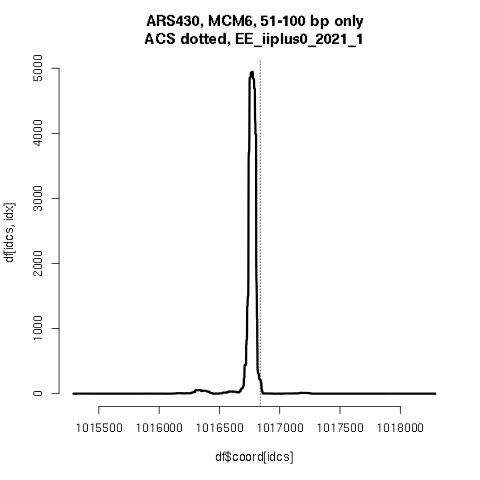

Supplement: S7 Source Files — (ZIP) [file pgen.1009714.s029.zip › source_files_for_S5Figure_to_S12Figure/S5_Figure/ARS430_chrIV_1016790_MCM6_51to100bp_lwd3_050921_1.tiff]

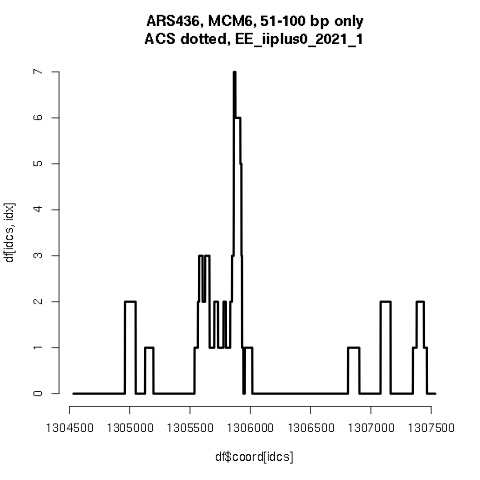

Supplement: S7 Source Files — (ZIP) [file pgen.1009714.s029.zip › source_files_for_S5Figure_to_S12Figure/S5_Figure/ARS436_chrIV_1306034_MCM6_51to100bp_lwd3_050921_1.tiff]

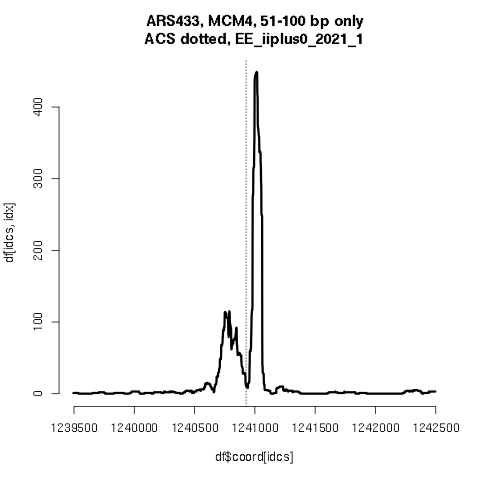

Supplement: S7 Source Files — (ZIP) [file pgen.1009714.s029.zip › source_files_for_S5Figure_to_S12Figure/S5_Figure/ARS433_chrIV_1240996_MCM4_51to100bp_lwd3_050921_1.tiff]

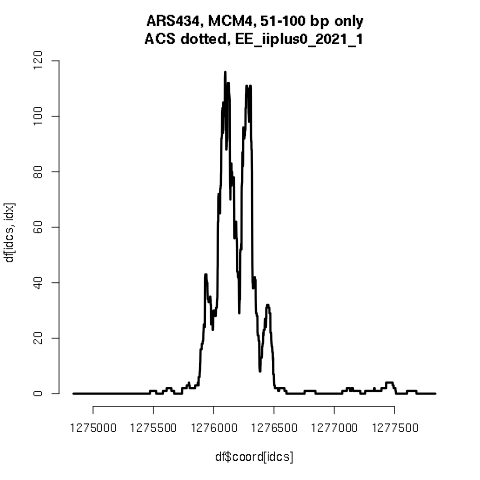

Supplement: S7 Source Files — (ZIP) [file pgen.1009714.s029.zip › source_files_for_S5Figure_to_S12Figure/S5_Figure/ARS434_chrIV_1276338_MCM4_51to100bp_lwd3_050921_1.tiff]

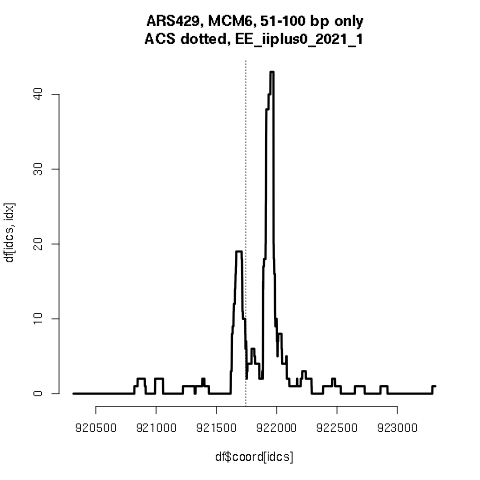

Supplement: S7 Source Files — (ZIP) [file pgen.1009714.s029.zip › source_files_for_S5Figure_to_S12Figure/S5_Figure/ARS429_chrIV_921815_MCM6_51to100bp_lwd3_050921_1.tiff]

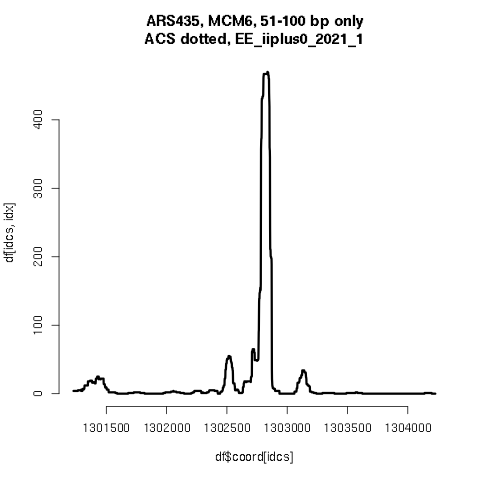

Supplement: S7 Source Files — (ZIP) [file pgen.1009714.s029.zip › source_files_for_S5Figure_to_S12Figure/S5_Figure/ARS435_chrIV_1302728_MCM6_51to100bp_lwd3_050921_1.tiff]

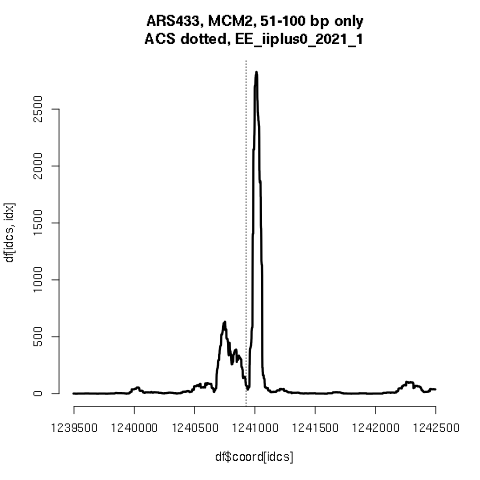

Supplement: S7 Source Files — (ZIP) [file pgen.1009714.s029.zip › source_files_for_S5Figure_to_S12Figure/S5_Figure/ARS433_chrIV_1240996_MCM2_51to100bp_lwd3_050921_1.tiff]

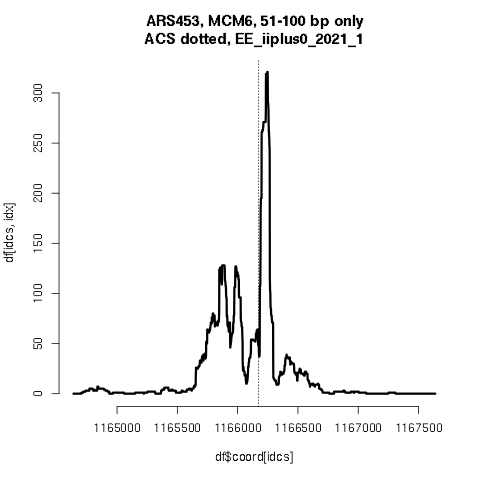

Supplement: S7 Source Files — (ZIP) [file pgen.1009714.s029.zip › source_files_for_S5Figure_to_S12Figure/S5_Figure/ARS453_chrIV_1166139_MCM6_51to100bp_lwd3_050921_1.tiff]

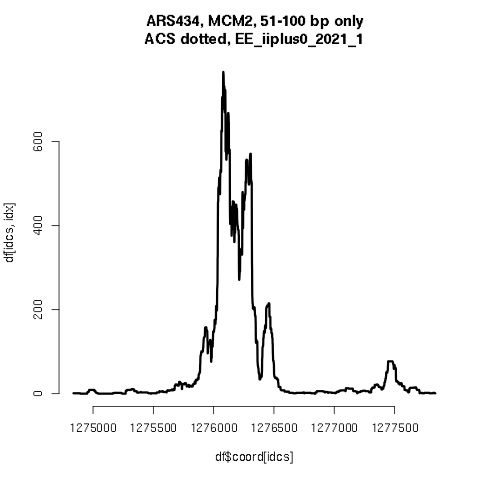

Supplement: S7 Source Files — (ZIP) [file pgen.1009714.s029.zip › source_files_for_S5Figure_to_S12Figure/S5_Figure/ARS434_chrIV_1276338_MCM2_51to100bp_lwd3_050921_1.tiff]

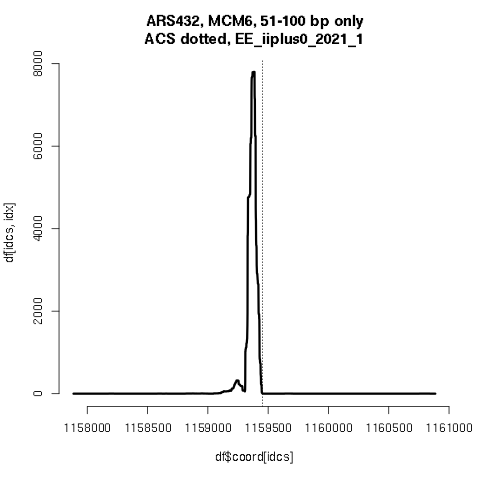

Supplement: S7 Source Files — (ZIP) [file pgen.1009714.s029.zip › source_files_for_S5Figure_to_S12Figure/S5_Figure/ARS432_chrIV_1159386_MCM6_51to100bp_lwd3_050921_1.tiff]

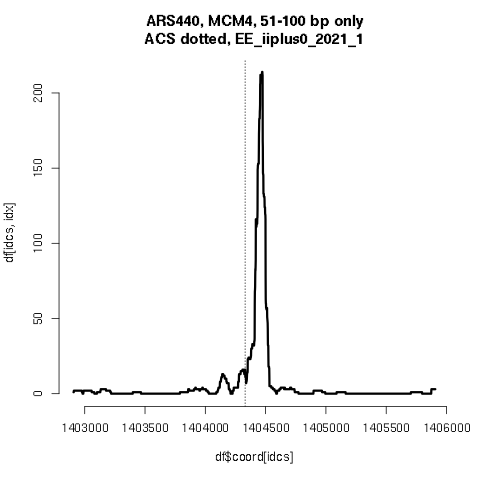

Supplement: S7 Source Files — (ZIP) [file pgen.1009714.s029.zip › source_files_for_S5Figure_to_S12Figure/S5_Figure/ARS440_chrIV_1404407_MCM4_51to100bp_lwd3_050921_1.tiff]

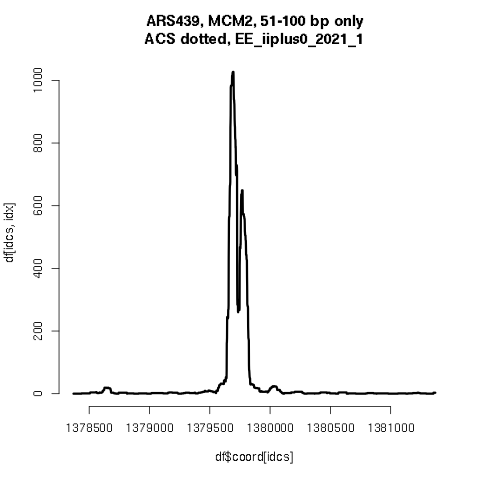

Supplement: S7 Source Files — (ZIP) [file pgen.1009714.s029.zip › source_files_for_S5Figure_to_S12Figure/S5_Figure/ARS439_chrIV_1379870_MCM2_51to100bp_lwd3_050921_1.tiff]

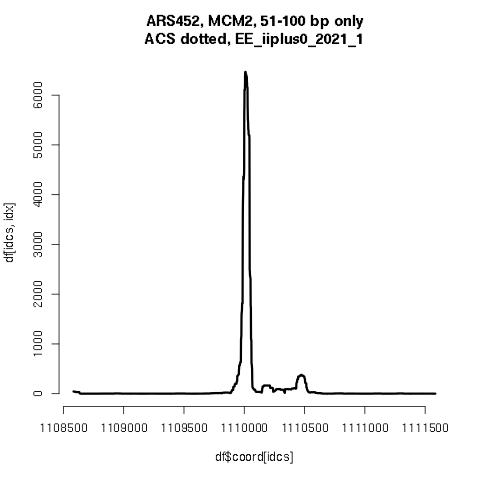

Supplement: S7 Source Files — (ZIP) [file pgen.1009714.s029.zip › source_files_for_S5Figure_to_S12Figure/S5_Figure/ARS452_chrIV_1110084_MCM2_51to100bp_lwd3_050921_1.tiff]

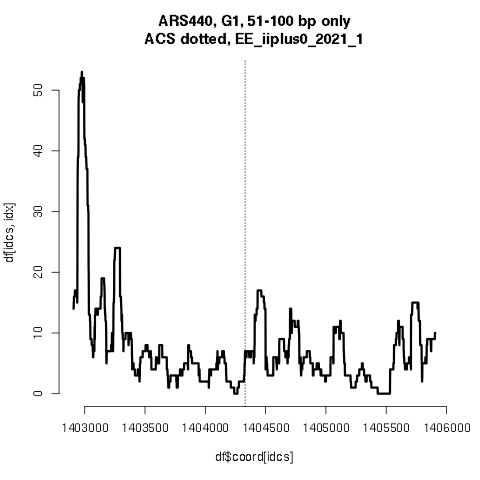

Supplement: S7 Source Files — (ZIP) [file pgen.1009714.s029.zip › source_files_for_S5Figure_to_S12Figure/S5_Figure/ARS440_chrIV_1404407_G1_51to100bp_lwd3_050921_1.tiff]

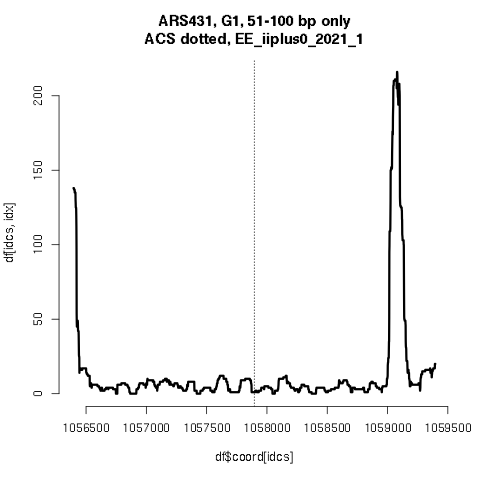

Supplement: S7 Source Files — (ZIP) [file pgen.1009714.s029.zip › source_files_for_S5Figure_to_S12Figure/S5_Figure/ARS431_chrIV_1057896_G1_51to100bp_lwd3_050921_1.tiff]

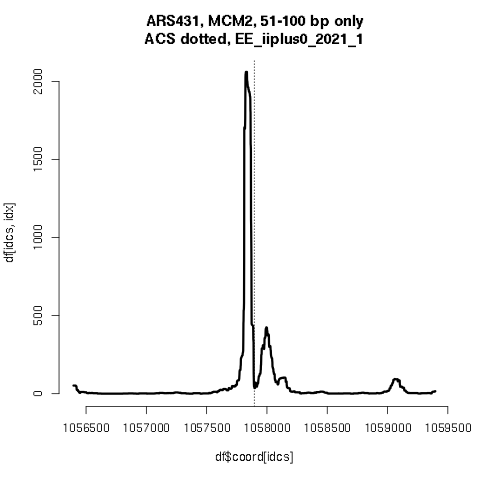

Supplement: S7 Source Files — (ZIP) [file pgen.1009714.s029.zip › source_files_for_S5Figure_to_S12Figure/S5_Figure/ARS431_chrIV_1057896_MCM2_51to100bp_lwd3_050921_1.tiff]

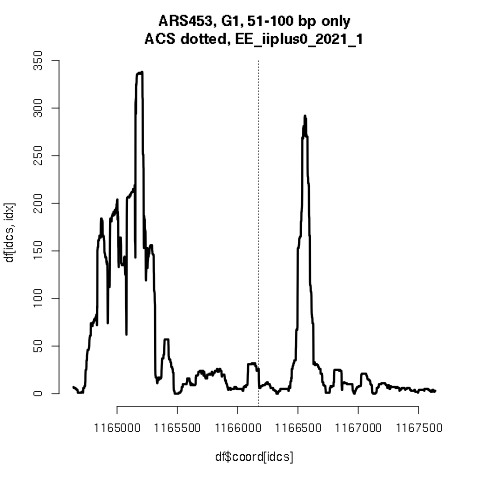

Supplement: S7 Source Files — (ZIP) [file pgen.1009714.s029.zip › source_files_for_S5Figure_to_S12Figure/S5_Figure/ARS453_chrIV_1166139_G1_51to100bp_lwd3_050921_1.tiff]

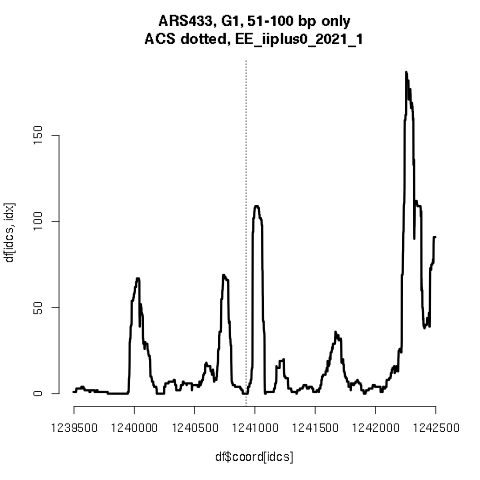

Supplement: S7 Source Files — (ZIP) [file pgen.1009714.s029.zip › source_files_for_S5Figure_to_S12Figure/S5_Figure/ARS433_chrIV_1240996_G1_51to100bp_lwd3_050921_1.tiff]
