# Supplementary material for: Chromosomal Mcm2-7 distribution and the genome replication program in species from yeast to humans
Source: PLoS Genet. 2021 Sep 2;17(9):e1009714. doi: 10.1371/journal.pgen.1009714 (PMC8443269; doi:10.1371/journal.pgen.1009714)
Supplement: S3 Table — (Word document). (DOCX) [file pgen.1009714.s021.docx]

S4 Table

| strain number | genotype | tagged subunit |
| --- | --- | --- |
| 16747 (*S. cerevisiae*) | MATa, his3, leu2, met15, ura3, hml-alpha::HYG, MCM2-MNase-3xFLAG::KanMX | MCM2 |
| 16749 (*S. cerevisiae*) | MATa, his3, leu2, met15, ura3, hml-alpha::HYG, MCM4-MNase-3xFLAG::KanMX | MCM4 |
| 16753 (*S. cerevisiae*) | MATa, his3, leu2, met15, ura3, hml-alpha::HYG, MCM6-MNase-3xFLAG::KanMX | MCM6 |
| 17000 (*S. pombe*) | h-, MCM2-MNase-3xFLAG::KanMX | MCM2 |
