# Supplementary material for: Chromosomal Mcm2-7 distribution and the genome replication program in species from yeast to humans
Source: PLoS Genet. 2021 Sep 2;17(9):e1009714. doi: 10.1371/journal.pgen.1009714 (PMC8443269; doi:10.1371/journal.pgen.1009714)
Supplement: Attachment — Submitted filename: Review_of_Foss_et_al_2020_PLoS_Genetics.pdf [file pgen.1009714.s035.pdf]

Review of: “Chromosomal Mcm2-7 distribution is the primary driver of the genome replication program in species from yeast to humans”

Synopsis:

In this work from Foss et al. the authors perform genome-wide mapping of the binding sites for core MCM components of the pre-replication complex in multiple organisms; specifically, they examine MCM2, MCM4 and MCM6 binding in budding yeast and MCM2 binding in fission yeast, mouse Patski cells and human Hela cells. Binding sites are mapped by tagging MCM proteins with micrococcal nuclease and sequencing the resultant digestion products; this technique yields a spatially precise readout, and this precision is important for the inferences made by the authors regarding binding of the MCM complex. Overall, the authors find that in *S.cerevisiae*, MCM proteins bind at known replication origins, however, it is difficult to discern if similar binding patterns occur in the other organisms. Indeed, overall, while the sections of the paper that deal with *cerevisiae* are relatively convincing, the data and analyses for the other three organisms require extensive work.

Major general comments:

The lack of controls are a major omission from the manuscript. Free MNase controls (as in the original ChEC-Seq paper [1]), ChEC-Seq in temperature sensitive mutants[2] and/or MCM ChEC-Seq controls in non-replicating cells would be very helpful. A comparison with published Mcm ChIP-Seq data may also be helpful to put the results of this method in context. Controls should be provided for all ChEC-Seq experiments, but are particularly important for *S. pombe*, mouse and human, where it is not clear to me if the method worked as the authors intended.

The analyses for mouse & human lack necessary depth and should be extensively revisited (see detailed comments).

The authors mention in the methods that “two biological replicas were prepared for each genotype” for *S. cerevisiae*, mouse and human cells (not sure about *S.pombe*). However, the authors do not provide information about the consistency across replicates for any of their analyses. The paper would greatly benefit from the addition of extensive details about replicates.

Overall, it is not clear what this paper adds to our body-of-knowledge of MCM loading. The most solid conclusion is that there appears to be a single MCM DH complex bound per origin (at least in *S. cerevisiae*), but there are caveats to this interpretation, not discussed by the authors. The other purported major findings are all questionable in my opinion.

The title of the paper does not reflect the findings. The authors have surveyed MCM2, 4 and 6 binding in *S. cerevisae* and just MCM2 binding in *S. pombe* and human cells (not MCM2-7, as

described in the title). Also, they do not provide evidence that the distribution of MCM proteins is the ***primary*** driver of the replication program. A substantially different title is necessary.

Minor general comments:

The manuscript would benefit from a more careful organization of figures. Many figures, panels and analyses appear out-of-order or interspersed with data that are not presented until far later in the paper. For example, parts of Figure 2A, 2B, 2C are not discussed in the text until after Figure 4, many pages after the figure is first mentioned.

The authors frequently write in absolutes, but should substantially temper their writing to acknowledge the intrinsic error in their measurements. Statements such as :"we identify […] *all* sites where replicative helicases have been loaded", "we observe *identical* MCM DH footprints", etc. should be re-worded.

Detailed comments:

Author summary:

Please make the opening less anthropomorphic and the findings less absolute.

Introduction:

There are numerous studies that have previously studied Mcm2-7 binding genome-wide in *S. cerevisiae*, yet these studies are not cited in this context. A more complete representation of the state-of-the-art and a more refined explanation of what this paper adds above-and-beyond these studies is crucial.

Results:

1. Pg. 6: "Because PCR involved in library preparation preferentially amplifies short fragments, which in turn are generated by MNase activity, we predicted that resulting libraries would reflect the sites at which Mcm2-7 DHs had been loaded"

I don't follow this logic; why are PCR amplification biases relevant? How long is considered "short"? Would these biases disfavor detection of longer fragments, potentially bound by multiple MCM complexes? Please expand.

2. *Pg. 6: "Strains with tagged Mcm proteins exhibited growth rates comparable to those in wild type, indicating that the presence of the tag did not perturb protein function"*

The yeast is grown in rich media, but is it possible that deficiencies in protein function are only observed if cells are grown under more challenging conditions? Is the amount of tagged MCM

protein in transformed cells comparable to untagged MCM in wild-type? It would be useful to explicitly clarify in the text that the endogenous locus was tagged.

3. *Page 6: General comment:*

Other labs have previously mapped MCM2 binding sites in *S. cerevisiae* using ChIP-Seq instead of ChEC-Seq. Please offer a brief comparison to demonstrate the commonalities of the data from the two approaches and the advantages conferred by ChEC-Seq.

4. *Figure 1:*
   - It would be useful to add some annotation to panel A, B & C (and all similar panels throughout the paper) to indicate the locations of ARS consensus sites.

   - The figure depicts a globally similar pattern of MCM2/4/6 in the genome, however it is important to also compare these datasets at high resolution. A brief glance at the authors' data suggested to me that patterns are broadly similar but that there are differences in the fine-scale patterning of MCM2/4/6 at the some loci (ARS1405, 1406, for example). A more systematic analysis for all three MCM2/4/6 datasets would be useful and showing all three datasets for the supplemental heatmaps would be a good idea. Again here, analysis of replicates would help to tease out what, if any, are the differences between binding of these three proteins.

   - Are the color density scales for all heatmaps the same? Please provide a color density legend for each heatmap. The same should be done for all heatmaps in the paper. What quantity is shown? Is it raw read density or is this value normalized?

   - Data for controls should also be shown here.

5. *Fig. S2:*

- The authors suggest that all three experiments yield data consistent with the capture of 62bp fragments, yet it looks like there are substantial differences between the three experiments. Notably, the short (<100bp) MCM6 fragments appear to be something like 50% longer than the short MCM2/4 fragments. MCM4 fragments also seem shorter than those for MCM2, but it is difficult to see. Information on replicates would be useful here. Please comment on these differences.

- It is difficult to see the MCM4 distribution. It would be helpful to show the three as separate graphs. It would also be useful to indicate the median of the short fragment distribution for each experiment.

6. *Figure 2:*

From the top panel, it appears that the captured fragments from Mcm2 differ in size across the three species. Only in *S. cerevisiae* is there the enrichment of fragments in the 62bp range; in pombe, the median size is more like 80 bp, in human, it appears that there is no favored size in the range shown. There also appear to be substantial differences in the distribution of MCM-ChEC fragments at origins in each species. Also, it is not clear why the data from mouse are not shown here. Please discuss.

7. *Fig. S3*:

I agree with the authors that one need not invoke two MCM DHs to explain the double peak. However, I don't think that the lack of the "red fragment" is sufficient to indicate that double binding does not occur. Is it not possible that the MNase would simply digest the unbound DNA between two bound MCM hexamers?

- Is this a plot of ARS411(heatmap) or of ARS1118 (legend)? Actually, I think it's ARS1411.
- Please detail what the green lines represent in the legend and indicate the distance between them.
- Please provide the number of tags on the y-axis of the bottom panel.
- Please indicate the location of the ARS (center, I assume)

8. *Fig. S4:*

Several of the ARS sites lack labels. Please rectify.

9. *Page7: "Furthermore, G1-specific and Cdc6-dependent footprints".*

Please explain the rationales for these experiments to help non-experts. Also, please show examples at some of the loci studied here (perhaps as a supplementary figure) for the G1-specific and Cdc6-dependent MNase experiments.

10. *Page 7:*

Are MCM2/4/6 peaks constrained to only ARS consensus sites or are there additional peaks elsewhere? If so, some discussion and analyses of these additional peaks would be welcome:

are they weak? Are they common to all 3 experiments? Do they coincide with putative ARS sequences? Etc.

11. Page 7:

Previous work has indicated that multiple MCM-loading occurs at some origins in yeast (e.g. [3]). Can the authors compare their data at these ARS sites to check if their findings support or contradict the previous conclusions.

12. Figure 4:

(A) Please provide some measure of variance around the mean for each bin. Also, please provide the number of genomic intervals per bin. (B) Please define the units for the y-axes. (C,D) Why were per chromosome and not genome-wide measurements made here? Are there differences between chromosomes? Please provide details of the tests used to calculate P-values. (C) Please reconcile the numbers in the legend (67,884, 61,214, etc.) with the numbers on the y-axis of the figure.

13. Page 8 / Fig. S6:

Please indicate the origin locations on each panel of Fig. S6. I have interpreted these graphs to be centered at the origin … If this interpretation is correct, do the authors have any thoughts as to why the Mcm2 signal is displaced from the origin?

A second question relates to data quality. The images for *pombe* are clearly noisier than those for *cerevisiae* (which are remarkably lacking in any background signal). Can the authors offer some thoughts or explanation as to why this may be?

14. Page 8: "Which in turn, drives the replication initiation pattern in S. pombe"

These data suggest that MCM loads at AT-rich sites bound by the ORC complex. In turn, AT-content, ORC density and MCM density all correlate with the replication initiation landscape. As written, it seems as though the authors find evidence for MCMs' role in patterning but to me, their data seem to imply that MCM loading is fully dependent on ORC localization and binding, consistent with previous knowledge. I suggest that the authors revisit this phrase to highlight what it is they think their study adds to this picture.

15. Page 9: "using a lentiviral vector, choosing an expression level that did not exceed physiological levels" / Fig. S8:

It appears that the expression level of endogenous MCM2 is substantially elevated in the transformed cell lines. The MNase-MCM2 expression is similarly elevated with respect to wild-type in mouse cells. Please quantify the relevant bands shown in the gels and discuss the potential impact of a large excess of MCM2 in these cells. Please define what is meant by “physiological levels” in the text. Does the presence of endogenous MCM2 change how these experiments should be interpreted compared to the experiments in yeast?

16. Page 9 / Figure 2 B,C:

The authors interpret the *cerevisiae* vs others differences as differences in MCM activity / binding, however their metrics are equally consistent with a less efficient experimental setup in organisms other than *S. cerevisiae* (the organism in which the assay was developed and refined). Indeed, for human in particular there appears to be little, if any enrichment. Good controls are essential here to explore the more technical aspects of whether the experiments worked in the non-*cerevisiae* organisms.

Overall, I am not convinced that the Mcm2 ChEC-Seq experiment worked in the human / mouse cells. Correlation with the pattern of replication timing could simply reflect the underlying biases in genome accessibility / patterns of euchromatin & heterochromatin. The authors should analyze these datasets in extensively more depth, examining the Mcm2 distribution around known origins of replication in both species and performing matched controls for these experiments.

17. Page 7-10 & throughout paper:

The authors refer to Mcm2-7 footprints. In *S. cerevisiae*, their data broadly support this type of generalization, yet in *S. pombe*, Hela and Patski cells, they only have data for Mcm2. They should be more explicit in describing their data in these organisms and not generalize to all MCMs.

18. Page 10 / Figure 5:

It is not clear what the baseline expectation is for these graphs, since many properties of the genome correlate with replication timing. Controls are absolutely required. How do these plots look if the authors instead used data from MNase-Seq experiments? Have the authors considered looking in high resolution at sites of replication origins? Are there “peaks” in the Mcm2 ChEC-Seq data, as in yeast?

19. *“indicating that the basic architecture of the complex is conserved.The MCM signal distribution along the chromosome in HeLa cells was more disperse than in budding yeast and similar to that in fission yeast”*

It is not clear what aspect of the “basic architecture” is conserved. In *S. cerevisiae*, there is a narrow peak derived from short (50-75bp) fragments, in *S.pombe*, there are broad MCM “zones” composed of 70-90 bp fragments, and in human, there appear to be sort-of clustered MCM2-ChEC reads with little evidence of any size preference. The pictures look very different to me. To that end, could the authors comment on why the human data look more binary than those for yeast and lack the read density gradient (Fig. 2A). Finally, could the authors include mouse data here too?

20. Page 9/10: Replication modelling

Could the disparities between the modelled and the experimental RT in repetitive regions result from difficulties in accurately mapping Mcm ChEC-Seq reads in repetitive regions?

Does the use of ChEC-Seq estimates give better / worse / comparable models compared to other measures of origin firing (Mcm2 ChIP-Seq / ORC binding / open chromatin)?

21. Figure 6:

It is very difficult to reconcile the written text with the data in this figure. Part of the reason this is challenging is the lack of specific quantities on the y-axes. I do not see evidence that replication of the inactive X is markedly delayed (in 6A)? To me, it looks like there are large regional fluctuations on the inactive X that are not seen on the active X, but it is not clear what this means. The authors should assure that appropriate units are provided for all figures.

Another concern is that mapping issues may contribute to these unusual patterns. These may arise from read mapping biases from using the mm10 reference (B6) genome; Spretus-specific indels may also play a role. A quick examination of the spretus and musculus bedgraph files in the GEO submission (GSE160775_musculus_G1_bwa_110519_1.bedgraph, GSE160775_musculus_S_bwa_110519_1.bedgraph, GSE160775_spretus_G1_bwa_110519_1.bedgraph) shows unusual patterns of coverage; specifically, coverage varies something like 10-fold among chromosomes (within each file), but also, some chromosomes are heavily enriched in Spretus (chr4, chr11), while others are heavily enriched in Musculus (chr19). The authors should investigate and explain these unusual patterns and demonstrate expansively that mapping issues are not a concern.

Discussion:

22. Page 13:

The authors conclude that a single MCM DH likely loads at most origins in every cell. This is OK, but I would be interested in knowing the authors' opinion on potential biases in the MCM-ChEC-Seq method; for instance, the authors allude to an ambiguous PCR bias for shorter fragments on Page 6. Is bead-based size selection used for ChEC-Seq as per the original protocol? Is it possible that the method disproportionately excludes fragments derived from loci at which multiple, closely spaced MCM complexes are loaded? If two MCM DH complexes sit adjacent to each other, the C-termini MNase tags will potentially abut; can the MNase always cut in between? Perhaps these caveats could be discussed.

23. Page 14: The 100 kb initiation zone

What is the "100 kb initiation zone in mammals"?

Methods:

24. Methods:

Generally, the methods could be substantially improved. Far more details are required to negate the need for the reader to consult numerous other papers.

25. Please provide version numbers for all bioinformatics tools used.

26. *Methods: Tagging Mcm with MNase*

"Finally, to restore the Mcm2 protein sequence to wild type, the Trp 185 residue was changed to Arg" ...what is this mutation? Please explain in more detail.

References

1. Zentner GE, Kasinathan S, Xin B, Rohs R, Henikoff S. ChEC-seq kinetics discriminates transcription factor binding sites by DNA sequence and shape in vivo. Nat Commun. 2015;6: 8733.

2. Foss EJ, Gatbonton-Schwager T, Thiesen AH, Taylor E, Soriano R, Lao U, et al. Sir2 suppresses transcription-mediated displacement of Mcm2-7 replicative helicases at the ribosomal DNA repeats. PLOS Genetics. 2019. p. e1008138.

doi:10.1371/journal.pgen.1008138

3. Das SP, Borrman T, Liu VWT, Yang SC-H, Bechhoefer J, Rhind N. Replication timing is regulated by the number of MCMs loaded at origins. Genome Res. 2015;25: 1886–1892.
