# Supplementary material for: Chromosomal Mcm2-7 distribution and the genome replication program in species from yeast to humans
Source: PLoS Genet. 2021 Sep 2;17(9):e1009714. doi: 10.1371/journal.pgen.1009714 (PMC8443269; doi:10.1371/journal.pgen.1009714)
Supplement: Attachment — Submitted filename: Review_of_Foss_et_al_resubmission.pdf [file pgen.1009714.s037.pdf]

Review of PGENETICS-D-20-01578_R1:

First, I would like to mirror the thoughts of the other reviewers to state that the experiments presented in this work certainly have the potential to offer new insights into MCM localization and activity. However, the authors continue to lean into conclusions not supported by the data, have not addressed many points raised by reviewers and many of the new analyses and datasets do not satisfactorily address the concerns raised during peer review. In many ways, the authors' response has been quite frustrating.

Major critiques:

1. The inclusion of control and replicate data is inconsistent and opaque (see below).
2. Threaded through the manuscript is the assertion that the multiplicity of MCM binding can be inferred using ChEC-Seq. The authors highlight this as an advantage of ChEC-Seq over ChIP-Seq, and assert that it allows them to demonstrate that one MCM binds per origin in *S. cerevisiae*. Nonetheless, the authors agree in response #7 that this type of inference is not possible and I see no other evidence to back up this claim. They have ignored the suggestion to compare ChEC-Seq to origins previously found to bind multiple MCMs and continue to assert that their findings contradict this previous work.
3. The authors have not responded to many of my suggestions (and those of the other reviewers). In many cases, they do so without providing a rationale.
4. The analyses of the data from pombe, mouse and human have only marginally improved and remain unconvincing. Most frustrating is that while a key aspect of this method is the ability to resolve beyond the limits of techniques such as ChIP-Seq, the authors have not performed any high resolution analyses to demonstrate that the method worked in these other organisms.
5. The authors have not clarified what this study adds to our body of knowledge.

Detailed critiques:

Controls:

Although the authors have added MNase controls to some figures, several things remain unclear:

1. Did the authors themselves perform the control experiments in conditions matched to those of the ChEC-Seq experiments? Several recent papers have debated the merits of ChEC-Seq [1–4], and it seems clear that the MNase controls should be closely matched to the experiment performed[5]. It is not clear from the details provided by the authors if that is the case here. For HeLa cells, the authors used published data. It seems unlikely that this is a sufficiently well matched control.

2. The authors claim that the pattern of cleavage using MCM-ChEC-Seq is notably different to the MNase controls, however in the single figure that shows the per-locus data side-by-side (in cerevisiae, Figure S5), the patterning is remarkably similar at some loci.

Indeed, the biggest difference in some cases appears to be the unusually low background in the ChEC-Seq experiments. Since the authors have not complied with my request to provide numerical metrics of read density in the heatmaps (which I still disagree with), it is impossible to tell if the differences between the MNase coverage and ChEC-Seq are simply the result of coverage imbalances. I attempted to get this information from one of the supplementary tables, however the MNase experiments are not listed in the table. Thus, I cannot conclude much from these data as presented. Controls should be presented far more clearly and unambiguously.

3. The authors state that:

   "Of particular note is our observation that they do not show the preferential association with replication initiation sites over replication termination sites that we see with Mcm-ChEC in S pombe (S10 Fig) and HeLa cells (S16 Fig)."

   I would caution the authors that while replication initiates at relatively well defined loci, termination seems likely to be more stochastic, spread across a far wider region than initiation is in most cases. Thus, these side-by-side comparisons of initiation vs termination regions should be taken with this precaution in mind and are not suitable as the primary evidence that their experiments are working.

4. The authors provide numerous scatterplots to compare ChEC-Seq to free MNase. These comparisons raise multiple questions. First, the authors should explicitly explain what exactly they are plotting. Is it all reads, or reads of a particular size? In cerevisiae (Fig. S2), the scatterplots are generated using 100 bp windows, which is reasonable, as it reflects the scale of the expected ChEC-Seq signal. However, in other organisms, the authors have performed these comparisons in 10Kb bins across the entire genome. This is the wrong scale for this comparison and much of the correlation (or lack thereof) is potentially driven by reads derived from the genomic background (S13, S17). Finally, the correlations for ChEC-Seq are all a little unusual because they appear to lack any stochastic genomic background. Is this the result of some data processing not described in the figure legend? Such background appears to be absent in the heatmaps too but is not explained by the authors. This point was raised, but not addressed in my initial review. Importantly, it is not clear how much the correlation with MNase-Seq would improve if their analyses were restricted to just the places where MCM-ChEC-Seq shows a "peak" or signal of some sort. This should be explored in depth.

5. The authors do not show any data for replicates at high resolution. This is essential to validate the consistency of their technique.

Figure S3:

The data for individual panels are not the same as the grouped panel. In particular, the MCM6 data are shifted.

Reviewer 1; point 6:

Figure S12B: The authors provide a new figure that helps to better infer the MCM2 ChEC-Seq fragment length in different species. This figure corroborates the issue I raised in my review but the differences between species are still not discussed by the authors.

Reviewer 1; point 7:

The authors agree that their method cannot distinguish between double MCM peaks and multiple binding sites for single MCMs. Nonetheless, they persist in stating that an advantage of ChEC-Seq is precisely that they can do this analysis, both in the legend of S6:

"indicates that the two MCM DH footprints at this origin mostly reflect signals that emanate from two populations of cells, each with a single MCM DH"

and in the introduction:

"Mcm ChIP, while adequate for determining the relative MCM abundance among the origins,lacks the necessary resolution to unambiguously resolve the exact number of loaded MCM complexes. The high resolution of the ChEC technique overcomes this barrier and thus enabled us to address the question directly"

Similar comments are also made in the discussion and should be rectified.

Review point 11:

The authors have not performed this analysis and have ignored this suggestion. Instead, and despite their acknowledgement that ChEC-Seq ***cannot*** make inferences about binding multiplicity, the authors persist in claiming that:

"Our results contradict the previous conclusions by Das et al., and we now note this in the Discussion."

Das et al. showed that multiple MCMs appear to load at yeast origins. At one origin they studied in detail (ARS1; aka ARS416), they observed that three MCM complexes load at this locus on each plasmid in their system. A quick glance at Figure S4 shows multiple (2-4) peaks at this locus using ChEC-Seq. Given these data together, I would suggest that in contrast to the assertions of the authors, the ChEC-Seq experiment may instead be showing exactly the expected pattern if multiple MCMs load at the same time.

Review point 12: (Fig 6)

The authors have inexplicably provided variance estimates for the figure as a separate table instead of as error bars. I understand that the standard deviations are huge relative to the mean, but that is exactly why they should be shown on the graph! A boxplot would be a better solution and this should be remedied. Also, it is not clear why the inclusion of other chromosomes would make "computational analyses simpler and less error-prone". Although this is commendably

honest, the authors should explain what “errors” arise when using other chromosomes? Finally, the numerical values on the y-axis still do not match the (revised) numbers in the figure legend.

Reviewer 1; point 15:

This point was modified in one place in the text but not others. It should be checked throughout.

Reviewer 1; point 18:

The authors have neither addressed my request to examine MCM2-ChEC Seq at high resolution in human cells, nor explained why they have ignored this request. High resolution proxies for origins of replication in human include ORC2 ChIP-Seq [6], Ini-Seq [7] and SNS-Seq in HeLA cells [8](GSE134988). SNS-Seq data from mouse are also available (Cayrou et al., 2015) & (Almeida et al., 2018). These analyses are essential to understand if these experiments worked in human & mouse cells. As mentioned above, it is not clear if published MNase data are an apt control for ChEC-Seq.

Reviewer 1; point 19:

In human, although some initiation appears to occur in zones spanning tens of Kb, experiments such as those mentioned above also identify discrete sites that represent putative origins of replication of <1 Kb in width. These could be assessed.

I also disagree that showing mouse data in Figure 2A is redundant. There is no figure that shows high-resolution mouse data, analogous to figure 2A (top). Given the high-resolution nature of the ChEC-Seq protocol, this is essential.

Reviewer 1; point 22:

The authors have not responded to my questions.

Reviewer 1; point 23:

This point still requires clarification.

References:

1. Donczew R, Lalou A, Devys D, Tora L, Hahn S. An improved ChEC-seq method accurately maps the genome-wide binding of transcription coactivators and sequence-specific transcription factors. Cold Spring Harbor Laboratory. 2021. p. 2021.02.12.430999. doi:10.1101/2021.02.12.430999

2. Mittal C, Rossi MJ, Franklin Pugh B. High similarity among ChEC-seq datasets. doi:10.1101/2021.02.04.429774

3. Bruzzone MJ, Albert B, Hafner L, Kubik S, Lezaja A, Mattarocci S, et al. ChEC-seq: a robust method to identify protein-DNA interactions genome-wide. Cold Spring Harbor Laboratory. 2021. p. 2021.02.18.431798. doi:10.1101/2021.02.18.431798

4. Zentner GE, Policastro RA, Henikoff S. ChEC-seq produces robust and specific maps of transcriptional regulators. Cold Spring Harbor Laboratory. 2021. p. 2021.02.11.430831. doi:10.1101/2021.02.11.430831

5. Zentner G. Chromatin Endogenous Cleavage and high-throughput sequencing (ChEC-seq) in S. cerevisiae v1 (protocols.io.bgthjwj6). protocols.io. ZappyLab, Inc.; 2020. doi:10.17504/protocols.io.bgthjwj6

6. Miotto B, Ji Z, Struhl K. Selectivity of ORC binding sites and the relation to replication timing, fragile sites, and deletions in cancers. Proc Natl Acad Sci U S A. 2016;113: E4810–9.

7. Langley AR, Gräf S, Smith JC, Krude T. Genome-wide identification and characterisation of human DNA replication origins by initiation site sequencing (ini-seq). Nucleic Acids Res. 2016;44: 10230–10247.

8. Long H, Zhang L, Lv M, Wen Z, Zhang W, Chen X, et al. H2A.Z facilitates licensing and activation of early replication origins. Nature. 2020;577: 576–581.
