# Supplementary material for: Chromosomal Mcm2-7 distribution and the genome replication program in species from yeast to humans
Source: PLoS Genet. 2021 Sep 2;17(9):e1009714. doi: 10.1371/journal.pgen.1009714 (PMC8443269; doi:10.1371/journal.pgen.1009714)
Supplement: Attachment — Submitted filename: Cover_ResponsesToReviewsFoss.docx [file pgen.1009714.s036.docx]

Dear Micheal,

We are submitting a revised manuscript. I would like to thank you for your patience as it took some time to carry out additional experiments and data analyses suggested by the reviewer. We believe we have addressed all the major concerns that were raised during the review and we hope that you will accept our revised manuscript for publication.

Sincerely,

Antonio Bedalov

Responses:

Dear Tony,

Thank you very much for submitting your Research Article entitled 'Chromosomal Mcm2-7 distribution is the primary driver of the genome replication program in species from yeast to humans' to PLOS Genetics. Also, thank you for making the additional information requested available to reviewers. I regret the extended period that the paper was out for review, mainly due to a delay in receiving one of the reviews.

The manuscript was fully evaluated at the editorial level and by independent peer reviewers. The reviewers appreciated the attention to an important problem, but raised substantial concerns about the current manuscript. Based on the reviews, we will not be able to accept this version of the manuscript, but we would be willing to review a much-revised version. We cannot, of course, promise publication at that time.

Below are some specific comments that I (ML) have, which I hope will be helpful in crafting your revision and response. You need not respond to them specifically since they are also comments made by the reviewers (to which you should respond, of course).

1. There was a general feeling that the paper would benefit from more in-depth analysis and discussion; to me the paper feels as if it had been originally written with page/word limits in mind. This should not be a concern with an online journal like PLOS Genetics. The Materials and Methods section is particularly brief.

We have carried out additional analyses as suggested by the reviewers and extended both Discussion and Material and Methods sections.

2. There was also a concern that the ChEC-Seq approach was not sufficiently validated, particularly in S. pombe and in mammalian cells. To reiterate one reviewer's comment, if high-efficiency origins have been identified in these cells (where admittedly most are not), perhaps those could be used for validation.

The most in-depth characterization of replication initiation in *S. pombe* and human cells were carried out by polymerase-usage sequencing (Pu-Seq) and Okazaki fragment sequencing, respectively. These are the data that we used to ask whether our MCM-ChEC seq results match the observed replication initiation pattern in these species.

We have added controls with free MNase, as suggested by the reviewers, in both *S pombe* and human cells. These controls show that the pattern of DNA digestion that results from exogenously added MNase is different from that obtained by Mcm2-ChEC, thus strengthening our claim that the Mcm2-ChEC signal reflects MCM distribution and not simply sensitivity to MNase digestion.

3. There was also a concern that the tagged Mcm constructs in budding yeast were not fully functional. I don't think colony size is sufficient. Since you used FACS-sorting to isolate G1 and S phase populations, it should be relatively straightforward to quantify the fraction of cells in G1, S and G2 in tagged and untagged strains as a test.

We have compared cell cycle profiles in tagged and untagged cells, as suggested.

Should you decide to revise the manuscript for further consideration here, your revisions should address the specific points made by each reviewer. We will also require a detailed list of your responses to the review comments and a description of the changes you have made in the manuscript.

**Reviewer 1.**

Synopsis:

In this work from Foss et al. the authors perform genome-wide mapping of the binding sites forcore MCM components of the pre-replication complex in multiple organisms; specifically, they examine MCM2, MCM4 and MCM6 binding in budding yeast and MCM2 binding in fission yeast, mouse Patski cells and human Hela cells. Binding sites are mapped by tagging MCM proteins with micrococcal nuclease and sequencing the resultant digestion products; this technique yields a spatially precise readout, and this precision is important for the inferences made by the authors regarding binding of the MCM complex. Overall, the authors find that in S.cerevisiae , MCM proteins bind at known replication origins, however, it is difficult to discern if similar binding patterns occur in the other organisms. Indeed, overall, while the sections of the paper that deal with cerevisiae are relatively convincing, the data and analyses for the other three organisms require extensive work.

**Major general comments:**

The lack of controls are a major omission from the manuscript. Free MNase controls (as in the original ChEC-Seq paper [1] ), ChEC-Seq in temperature sensitive mutants [2] and/or MCM ChEC-Seq controls in non-replicating cells would be very helpful. A comparison with published Mcm ChIP-Seq data may also be helpful to put the results of this method in context. Controls should be provided for all ChEC-Seq experiments, but are particularly important for S. pombe, mouse and human, where it is not clear to me if the method worked as the authors intended. The analyses for mouse & human lack necessary depth and should be extensively revisited (see detailed comments).

We have added free MNase analyses for S. cerevisiae, S. pombe and human cells, as suggested by the reviewer. These results, presented in S2, S5, S10, S13 and S16 Figs, demonstrate that the pattern of free MNase does not recreate the signal we see with Mcm-ChEC. Of particular note is our observation that they do not show the preferential association with replication initiation sites over replication termination sites that we see with Mcm-ChEC in *S pombe* (S10 Fig) and HeLa cells (S16 Fig).

The authors mention in the methods that “two biological replicas were prepared for each

genotype” for S. cerevisiae , mouse and human cells (not sure about S.pombe ). However, the authors do not provide information about the consistency across replicates for any of their analyses. The paper would greatly benefit from the addition of extensive details about replicates.

We have added replica results in *S cerevisiae*, *S. pombe*, mouse and human cells, as requested (S2, S13, S17) and the analysis of replicate sample (S10 and S16 Fig).

Overall, it is not clear what this paper adds to our body-of-knowledge of MCM loading. The most solid conclusion is that there appears to be a single MCM DH complex bound per origin (at least in S. cerevisiae), but there are caveats to this interpretation, not discussed by the authors. The other purported major findings are all questionable in my opinion. The title of the paper does not reflect the findings. The authors have surveyed MCM2, 4 and 6 binding in S. cerevisae and just MCM2 binding in S. pombe and human cells (not MCM2-7, as described in the title). Also, they do not provide evidence that the distribution of MCM proteins is the primary driver of the replication program. A substantially different title is necessary.

In the title and throughout the manuscript “MCM2-7” refers to a complex rather than the individual components of the complex. Given that Mcm2 is part of the complex, we used Mcm2 footprints as a proxy for the localization of the complex. In support of the validity of this approach, we show that the ChEC footprints generated by tagging of Mcm2, Mcm4 and Mcm6 in *S. cerevisiae* co-localize. Similarly, published MCM ChIP experiments typically immunoprecipitate one of the components to measure MCM complex abundance across the genome.

In the Discussion, we have highlighted the advantage of Mcm-ChEC over Mcm-ChIP with regard to resolution. We have also provided Southern blot and qPCR results to demonstrate the efficacy of cutting by Mcm-ChEC, and we have added analyses of single- versus multiple-footprint origins that strengthen our claim that most origins in budding yeast have either one or zero MCM DHs.

We have changed the title to moderate our claim that MCM distribution is the primary driver of replication timing.

The title has been changed from:

Chromosomal Mcm2-7 distribution is the primary driver of the genome replication program in species from yeast to humans

to:

Chromosomal Mcm2-7 distribution and the genome replication program in species from yeast to humans

Minor general comments:

The manuscript would benefit from a more careful organization of figures. Many figures, panels and analyses appear out-of-order or interspersed with data that are not presented until far later in the paper. For example, parts of Figure 2A, 2B, 2C are not discussed in the text until after Figure 4, many pages after the figure is first mentioned.

We made changes so that the figures are numbered in the order in which they are first referred to in the text. One issue with this approach is Figure 2A whose parts seem to be discussed out of order. This figure juxtaposes MCM distribution at both fine and chromosomal scale and chromosomal replication pattern in budding yeast, fission yeast and human cells. This arrangement simplifies the comparison among the species to better convey the link between MCM distribution and replication across the species. We feel that the benefit to the reader from this consolidation of results from different species in a single figure outweighs the potential inconvenience to the reader created by references to different parts of Figure 2A appearing throughout the text, as we move from one organism to another.

The authors frequently write in absolutes, but should substantially temper their writing to

acknowledge the intrinsic error in their measurements. Statements such as :“we identify […] all sites where replicative helicases have been loaded”, “we observe identical MCM DH footprints”, etc. should be re-worded.

We have re-worded the text to moderate our claims throughout the text as suggested.

Detailed comments:

Author summary:

Please make the opening less anthropomorphic and the findings less absolute.

We have rewritten the Author summary as suggested.

Introduction:

There are numerous studies that have previously studied Mcm2-7 binding genome-wide in S. cerevisiae , yet these studies are not cited in this context. A more complete representation of the state-of-the-art and a more refined explanation of what this paper adds above-and-beyond these studies is crucial.

We have cited several studies of genome wide MCM distribution by ChIP-seq and analyzed the correlation between relative abundance of origins in ChEC vs ChIP. In the discussion section, cited below, we discuss advantages of ChEC vs ChIP in addressing MCM stoichiometry at replication origins.

"One question that has attracted significant attention over the last two decades is whether multiple MCM complexes are loaded at individual origins in a single cell and, if so, whether increased numbers of helicases are associated with higher origin activity [45, 53]. ... Budding yeast is an ideal organism in which to address this question, due to sequence specificity of its replication origins; however, Mcm ChIP, while adequate for determining the relative MCM abundance among the origins, lacks the necessary resolution to unambiguously resolve the exact number of loaded MCM complexes. The high resolution of the ChEC technique overcomes this barrier and thus enabled us to address the question directly, as follows:"

Results:

1. Pg. 6: “Because PCR involved in library preparation preferentially amplifies short fragments, which in turn are generated by MNase activity, we predicted that resulting libraries would reflect the sites at which Mcm2-7 DHs had been loaded”

I don’t follow this logic; why are PCR amplification biases relevant? How long is considered “short”? Would these biases disfavor detection of longer fragments, potentially bound by multiple MCM complexes? Please expand.

Our goal was to minimize DNA manipulations like size selection to avoid sample loss. We reasoned that DNA fragments between origins, would not be amplified due to their large size, and thus there would be no need to carry out size selection prior to preparation of the library. In other words, we expected that PCR's bias toward amplification of small fragments would work in our favor when attempting to identify DNA fragments cleaved by Mcm-ChEC, and these expectations were borne out by our results. We have clarified this point in the text:

“Because PCR involved in library preparation preferentially amplifies short fragments, which in turn are generated by MNase activity, we predicted that resulting libraries would reflect the sites at which Mcm2-7 DHs had been loaded; in other words, fragments generated by cutting on either side of one MCM helicase complex should be much shorter than inter-origin fragments, and therefore should be preferentially amplified.”

2. Pg. 6: “Strains with tagged Mcm proteins exhibited growth rates comparable to those in wild type, indicating that the presence of the tag did not perturb protein function”

The yeast is grown in rich media, but is it possible that deficiencies in protein function are only observed if cells are grown under more challenging conditions? Is the amount of tagged MCM2 protein in transformed cells comparable to untagged MCM in wild-type? It would be useful to explicitly clarify in the text that the endogenous locus was tagged.

We have clarified in the text that we have tagged the single endogenous locus. The reviewer is certainly correct that normal growth rate does not rule out the possibility that a tag on an essential protein subtly alters protein function. We have therefore analyzed cell cycle profiles of the tagged strains reasoning that altered MCM function may increase the fraction of cell in S-phase (Supplemental ). As we could not detect changes in cell cycle distribution between tagged and untagged strains, we feel safe in concluding that the presence of the tag did not grossly perturb protein function under the conditions in our experiments. We have changed the text (changes bolded):

“Strains with tagged Mcm proteins exhibited growth rates and **cell cycle distribution** comparable to those in wild type, indicating that the presence of the tag did not **grossly** perturb protein function **under our experimental conditions**.”

3. Page 6: General comment: Other labs have previously mapped MCM2 binding sites in S. cerevisiae using ChIP-Seq instead of ChEC-Seq. Please offer a brief comparison to demonstrate the commonalities of the data from the two approaches and the advantages conferred by ChEC-Seq.

As suggested, we now report the correlation between MCM abundance measured by ChEC-seq and that in published ChIP-seq datasets. In addition we compared correlations between replication initiation pattern with Mcm-ChEC and Mcm-ChIP, as discussed in greater detail in the replies of reviewers 2 and 3. Lastly, we have discussed the advantages of ChEC in determining fine resolution of Mcm2 localization, specifically as it relates to determining the number of MCM complexes per origin.

4. Figure 1:

- It would be useful to add some annotation to panel A, B & C (and all similar panels

throughout the paper) to indicate the locations of ARS consensus sites.

There is not an objective distinction between sequences that do and do not conform to the ARS consensus sequence (ACS), and "reasonable" matches can be found at over 3,000 sites across the genome. Thus, adding all the ACS site would be impractical. We have noted ACSs that have been previously experimentally validated.

- The figure depicts a globally similar pattern of MCM2/4/6 in the genome, however it is

important to also compare these datasets at high resolution. A brief glance at the

authors’ data suggested to me that patterns are broadly similar but that there are

differences in the fine-scale patterning of MCM2/4/6 at the some loci (ARS1405, 1406,

for example). A more systematic analysis for all three MCM2/4/6 datasets would beuseful and showing all three datasets for the supplemental heatmaps would be a good idea. Again here, analysis of replicates would help to tease out what, if any, are the differences between binding of these three proteins.

We have correlated MCM2/4/6 relative abundances (S2 Fig).

We have also provided heat maps for MCM2 4 and 6 for 12 consecutive origins in S5 Fig.

- Are the color density scales for all heatmaps the same? Please provide a color density

legend for each heatmap. The same should be done for all heatmaps in the paper. What

quantity is shown? Is it raw read density or is this value normalized?

The purpose of the color density is to accentuate the relative read depth within a single image, and not to compare read depths between images. We feel that adding color scales will cause confusion, and have instead clarified this issue by changing the text as folows:

“In this report, we visualize individual Mcm2-7 footprints as heat maps, with fragment size plotted according to genomic location, and **relative** read depths **within each image** represented by color intensity [43].”

- Data for controls should also be shown here.

We have provided exogenous free MNase controls for cerevisiae, pombe and human cells.

5. Fig. S2:

- The authors suggest that all three experiments yield data consistent with the capture of 62bp fragments, yet it looks like there are substantial differences between the three experiments. Notably, the short (<100bp) MCM6 fragments appear to be something like 50% longer than the short MCM2/4 fragments. MCM4 fragments also seem shorter than those for MCM2, but it is difficult to see. Information on replicates would be useful here. Please comment on these differences.

We believe the fragment lengths to be sufficiently close to that suggested by cryo-EM studies, given that variations in the orientation of MNase attached to different subunits are expected to generate different size fragments, and we had noted the fragment size range in the text ("Furthermore, fragment size distributions for all three libraries peaked in the 50-62 bp size range, consistent with the 60-62 bp protected by Mcm2-7 DHs observed in cryo-electron microscopy studies [21, 22] (S3 Fig)."). In order to further allow the reader to pass judgment on this question, we have noted fragment sizes for the different constructs in the legend to S3Fig:

**S3 Fig.** Fragment sizes in libraries from S. cerevisiae strains with tagged Mcm2, Mcm4 and Mcm6 are comparable and consistent with the 62 bp of DNA shown to be protected by the MCM helicase complex by cryo-EM. Fragment sizes for the peaks of Mcm2-, Mcm4- and Mcm6-tagged strains are 58, 50 and 62 base pairs, respectively, and median fragment lengths for fragments <= 100 base pairs were 61, 58 and 70 base pairs, respectively.

- It is difficult to see the MCM4 distribution. It would be helpful to show the three as separate graphs. It would also be useful to indicate the median of the short fragment distribution for each experiment.

We provide size distribution in three separate graphs (S3 Fig) and we have calculated the medians, as noted above.

6. Figure 2:

From the top panel, it appears that the captured fragments from Mcm2 differ in size across the three species. Only in S. cerevisiae is there the enrichment of fragments in the 62bp range; in pombe, the median size is more like 80 bp, in human, it appears that there is no favored size in the range shown. There also appear to be substantial differences in the distribution of MCM-ChEC fragments at origins in each species. Also, it is not clear why the data from mouse are not shown here. Please discuss.

Heat maps like the one the reviewer refers to are not the best way to assess the size distributions in ChEC experiments, as they provide only a snapshot of a short (in this case 10 kb) region. Size distributions should be assessed instead with the plots that we have provided for budding and fission yeast and for human. We have now added such a plot for mouse (S17 Fig) as well as a joint plot for all four species (S12B).

7. Fig. S3 (Now renumbered to S6):

I agree with the authors that one need not invoke two MCM DHs to explain the double peak. However, I don’t think that the lack of the “red fragment” is sufficient to indicate that double binding does not occur. Is it not possible that the MNase would simply digest the unbound DNA between two bound MCM hexamers?

- Is this a plot of ARS411 (heatmap) or of ARS1118 (legend)? Actually, I think it’s

ARS1411.

Yes, it is ARS1411. Thank you pointing this out. We agree that the digestion of the fragments between two MCMs could make these fragments disappear. We recognize this possibility in the figure legend as follows:

“However, we cannot exclude the possibility that the absence of 75 bp fragments is not a consequence of their degradation.”

- Please detail what the green lines represent in the legend and indicate the

distance between them.

Vertical green lines represent 60 base pairs from the apex of the MCM-ChEC peak. We regret having forgotten to include this information in the legend to S6 Fig.,, and we have corrected this omission.

- Please provide the number of tags on the y-axis of the bottom panel.

We have labeled the Y-axis.

- Please indicate the location of the ARS (center, I assume)

We have corrected the legend to S6 Fig. to note that the yellow dotted line indicates the ACS.

8. Fig. S4:

Several of the ARS sites lack labels. Please rectify.

Thank you for pointing this out. We have added the labels.

9. Page7: “Furthermore, G1-specific and Cdc6-dependent footprints”.

Please explain the rationales for these experiments to help non-experts. Also, please show examples at some of the loci studied here (perhaps as a supplementary figure) for the G1-specific and Cdc6-dependent MNase experiments.

We eliminated the text that refers to to G1-specific and Cdc6-dependent footprints to simplify the presentation of data.

10. Page 7:

Are MCM2/4/6 peaks constrained to only ARS consensus sites or are there additional peaks elsewhere? If so, some discussion and analyses of these additional peaks would be welcome: are they weak? Are they common to all 3 experiments? Do they coincide with putative ARS sequences? Etc.

ARS consensus sequences are only loosely defined. Mcm-ChEC signals are not always next to ARS consensus sequences, but, by the same token, neither do budding yeast origins always contain obvious ACS sequences; for example, SGD lists ACS sequences for only about half of yeast origins. Mcm-ChEC peaks also do not always correspond to origins that are listed in SGD (or OriDB); this can be seen in the middle panel of the 3 cerevisiae panels in Figure 2A, since there are visible Mcm-ChEC peaks that do not coincide with red triangles. However, what does and what does not constitute a "peak" of MCM signal is a subjective judgement that depends, among other things, on read depth (the more you sequence, the more you'll find). We are currently working on a manuscript to address these issues in more depth, but we feel that it is beyond the scope of the current manuscript.

11. Page 7:

Previous work has indicated that multiple MCM-loading occurs at some origins in yeast (e.g. [3] ). Can the authors compare their data at these ARS sites to check if their findings support or contradict the previous conclusions.

We found a single footprint at approximately half (165 / 343) of the origins that are listed in SGD; moreover, these single footprint origins are among the most abundant ones, as shown in the new figures that we have added. We also provide a Southern blot and q PCR analysis of a ChEC time course at the highly efficient and abundant ARS 1103 that shows that at this single footprint origin MCM has been loaded in >90% of the cells. Our results contradict the previous conclusions by Das et al., and we now note this in the discussion.

12. Figure 4:

(A) Please provide some measure of variance around the mean for each bin. Also, please provide the number of genomic intervals per bin. (B) Please define the units for the y-axes.

We have provided these data in the supplemental Table.

(C,D) Why were per chromosome and not genome-wide measurements made here? Are there differences between chromosomes? Please provide details of the tests used to calculate P-values.

Genome-wide calculations gave similar results, and we have never seen differences between chromosomes that would suggest that there is anything atypical about chromosome II (which constitutes 36% of the genome). We restricted ourselves to chromosome II just because it made some computational analyses simpler and less error-prone.

p values were calculated with 2-sided t tests using R's "t.test" function.

(C) Please reconcile the numbers in the legend (67,884, 61,214, etc.) with the

numbers on the y-axis of the figure.

We had unintentionally listed absolute values in the legend to Figure 4C, whereas we plotted relative values. We are grateful to the reviewer for catching this error, and w have fixed it as follows:

“Mean value for initiations was 0.19 (relative per-base-pair read depth), with 95% confidence intervals extending from 0.17 to 0.21; for terminations, mean was 0.023, with 95% confidence intervals extending from 0.020 to 0.027, P=5.3x10^-46^ “.

13. Page 8 / Fig. S6:

Please indicate the origin locations on each panel of Fig. S6. I have interpreted these graphs to be centered at the origin … If this interpretation is correct, do the authors have any thoughts as to why the Mcm2 signal is displaced from the origin?

Figure S6 (now renumbered to S8) contains a set of 20 “confirmed” OrDB pombe origins that have been experimentally validated by 2D gels and genome-wide studies, but their published coordinates have not been “centered” according to the center of origin activity. Therefore, it is not surprising that Mcm2-ChEC signal is often off the center.

We have added the following text:

“Plots are centered on midpoints of origins, as listed in OriDB”.

A second question relates to data quality. The images for pombe are clearly noisier than those for cerevisiae (which are remarkably lacking in any background signal). Can the authors offer some thoughts or explanation as to why this may be?

We believe that the “noisier” appearance of *S. pombe* origins, compared to those in *S. cerevisiae*, is a reflection of differences of ORC binding preference in the two species, AT content and ACS, respectively. Compared to the discrete ACS sites, sliding AT content varies continuously across the genome, creating the appearance of “noisier” binding. When we measured the MCM abundance according to average AT content in 20 bp windows in figure 6A, we find high correlation between AT content and MCM abundance.

14. Page 8: “Which in turn, drives the replication initiation pattern in S. pombe”

These data suggest that MCM loads at AT-rich sites bound by the ORC complex. In turn,

AT-content, ORC density and MCM density all correlate with the replication initiation landscape. As written, it seems as though the authors find evidence for MCMs’ role in patterning but to me, their data seem to imply that MCM loading is fully dependent on ORC localization and binding, consistent with previous knowledge. I suggest that the authors revisit this phrase to highlight what it is they think their study adds to this picture.

We did not mean to imply that we were the first people to suggest this model. By changing "We conclude that ...." to "These observations support a model in which ...." and adding a reference to a publication with a similar view of replication in pombe, we hope that we have fixed this oversight.

“These observations support a model in which AT-content drives chromosomal MCM distribution, which in turn drives the replication initiation pattern in *S. pombe* [37].”

15. Page 9: “using a lentiviral vector, choosing an expression level that did not exceed

physiological levels” / Fig. S8:

It appears that the expression level of endogenous MCM2 is substantially elevated in the

transformed cell lines. The MNase-MCM2 expression is similarly elevated with respect to

wild-type in mouse cells. Please quantify the relevant bands shown in the gels and discuss the potential impact of a large excess of MCM2 in these cells. Please define what is meant by “physiological levels” in the text. Does the presence of endogenous MCM2 change how these experiments should be interpreted compared to the experiments in yeast?

A difference between the experimental design of both mouse and human ChEC compared to yeast ChEC is the presence of untagged Mcm2 in the former. When we carried out Mcm2-ChEC in diploid *S. cerevisiae* strain in which only one of the two copies of Mcm2 is tagged, we found a footprint distribution similar to that in haploid Mcm2-tagged strains, suggesting that it is not necessary for all the MCMs to contain a tagged component to determine MCM distribution.

Our primary interest in the Western blot in the Fig S8 was to quantify the relative abundance of tagged and untagged Mcm2; extracts from untagged cells were included in the Western blot as size controls without normalizing to total protein loaded.

The relative abundance of tagged vs untagged bands for human and mouse cells was 0.4- and 0.25- fold, respectively. Reduced abundance of tagged MCM2 vs untagged MCM2 reduces a potential for non-specific DNA cutting.

16. Page 9 / Figure 2 B,C:

The authors interpret the cerevisiae vs others differences as differences in MCM activity /binding, however their metrics are equally consistent with a less efficient experimental setup in organisms other than S. cerevisiae (the organism in which the assay was developed and refined). Indeed, for human in particular there appears to be little, if any enrichment. Good controls are essential here to explore the more technical aspects of whether the experiments worked in the non- cerevisiae organisms.

Overall, I am not convinced that the Mcm2 ChEC-Seq experiment worked in the human / mouse cells. Correlation with the pattern of replication timing could simply reflect the underlying biases in genome accessibility / patterns of euchromatin & heterochromatin. The authors should analyze these datasets in extensively more depth, examining the Mcm2 distribution around known origins of replication in both species and performing matched controls for these experiments.

The most in depth characterization replication initiation in *S. pombe* and human cells were carried out by polymerase usage sequencing (Pu-Seq) and Okazaki fragment sequencing, respectively. These are the exact data that we use to ask whether our MCM-ChEC seq results match the observed replication initiation pattern in two species. Pu-seq identifies replication initiation and termination sites as sites at which DNA polymerases diverge and converge, respectively. Our peaks of MCM-ChEC signal in pombe are strikingly congruent with the replication initiation sites identified by Pu-seq, whereas they do not coincide with the sites of termination (Fig 6B). We have quantified the difference between MCM abundance at replication initiation and termination sites, and we have now showed that this correlation does not exist in data using free MNase instead of MCM-ChEC (Fig S10). We feel that these observations provide compelling evidence that our MCM-ChEC worked in S. pombe.

Similar results in HeLa cells using the analysis of Okazaki Seq data sets, including free MNase controls were obtained in HeLa cells as discussed in point 18.

17. Page 7-10 & throughout paper:

The authors refer to Mcm2-7 footprints. In S. cerevisiae , their data broadly support this type of generalization, yet in S. pombe , Hela and Patski cells, they only have data for Mcm2. They should be more explicit in describing their data in these organisms and not generalize to all MCMs.

Mcm2-7 footprints refers to a footprint of a complex and not the footprint of individual comlex componets as discussed above in greater detail in our response to general comments.

18. Page 10 / Figure 5:

It is not clear what the baseline expectation is for these graphs, since many properties of the genome correlate with replication timing. Controls are absolutely required. How do these plots look if the authors instead used data from MNase-Seq experiments? Have the authors considered looking in high resolution at sites of replication origins? Are there “peaks” in the Mcm2 ChEC-Seq data, as in yeast?

As we discussed previously in point 16, the most in depth, high resolution characterization of replication initiation in human cells has been carried out by Petryk et al which employed Okazaki fragment sequencing for determining fork directionality. The fork directionality plots, such as those we show in Figure 7 and S14A-F, identified steep 50-150 kb initiation zones as sites where the fork directionality changes from blue to red, and less steep termination zones where the fork directionality changes in the opposite direction. We found that MCM2-ChEC enrichment spikes and MCM2-ChEC valleys coincide with these Okazaki-seq-defined initiation and termination zones, respectively. The reviewer is correct that many features of chromatin correlate with replication initiation, this has been shown in a study by Das et al. Accordingly, we observed marked differences in specific histone modifications at replication initiation and termination sites as shown in S11 Fig. Lastly, in S16 Fig, we show the analysis of free-MNase-seq, which show no enrichment at the initiation zones, as is evident from the fork directionality plots S16A Fig in and in global analysis of the abundance at initiation and termination zones in S16B Fig.

19. *“indicating that the basic architecture of the complex is conserved.The MCM signal*

*distribution along the chromosome in HeLa cells was more disperse than in budding yeast and similar to that in fission yeast”*

It is not clear what aspect of the “basic architecture” is conserved. In S. cerevisiae , there is a narrow peak derived from short (50-75bp) fragments, in S.pombe , there are broad MCM “zones” composed of 70-90 bp fragments, and in human, there appear to be sort-of clustered MCM2-ChEC reads with little evidence of any size preference. The pictures look very different to me. To that end, could the authors comment on why the human data look more binary than those for yeast and lack the read density gradient (Fig. 2A).

Finally, could the authors include mouse data here too?

We have now clarified that by “basic architecture” we refer to appearance of individual MCM footprints and the adjacent nucleosomes. We believe that the more binary appearance of human data compared to yeast data is a result of 1) sparse coverage of human genome compared to the coverage in yeast 2) wider distribution of MCM footprints the across the initiation zones in human cells compared to yeast. In budding yeast, the initiation and thus MCM footprints are restricted to often <100 bp corresponding to single MCM footprints, in S. pombe initiation zones are thought to be 500-1500 bp is size and correspond to regions of higher AT desnsity, where initiation zones is human cells are thought to be 100 kb in size.

We thought it would be redundant to show mouse data in Figure 2A because we show mouse chromosomal MCM distribution and replication in Figure 6 particularly given that Figure 2A is already sizable.

20. Page 9/10: Replication modelling

Could the disparities between the modelled and the experimental RT in repetitive regions result from difficulties in accurately mapping Mcm ChEC-Seq reads in repetitive regions?

All of our alignments were done using the -n 1 option in the bwa aligner; with this specification, bwa randomly assigns reads that map to more than one location to one of those locations. This gives us a "fair" representation of repeated sequences. Furthermore, if we eliminate all reads that map to more than one location, we still see the same disparities.

Does the use of ChEC-Seq estimates give better / worse / comparable models compared to other measures of origin firing (Mcm2 ChIP-Seq / ORC binding / open chromatin)?

We carried the suggested analysis and provide it in response to reviewer 3.

21. Figure 6:

It is very difficult to reconcile the written text with the data in this figure. Part of the reason this is challenging is the lack of specific quantities on the y-axes.

I do not see evidence that replication of the inactive X is markedly delayed (in 6A)? To me, it looks like there are large regional fluctuations on the inactive X that are not seen on the active X, but it is not clear what this means. The authors should assure that appropriate units are provided for all figures.

In S-seq experiments the replication across the chromosome is calculated as a change in relative abundance or reads along the chromosome in cells isolated in S-phase fraction relative to G1 fraction. A delayed replication of a chromosome, compared to other chromosome, as it happens on the inactive X is expected to reduce the fluctuations on that chromosome compared to autosomes. This is exactly what we see in both out S-seq data in 6A for the mouse and in similar analysis of repli-seq data for human cells in 6B.

The scale on the Y-axis in 9A is identical for chromosome 16 and chromosome X and shows relative distribution of read depths along two chromosomes separately by the parent of origin. Given that the scale is identical, one can directly compare relative fluctuations for each of the parents for both chromosomes. The magnitude of fluctuations for the spretus chromosome is similar for chromosome 16 and X, whereas the fluctuations for musculus chromosome is lower on the X compared to chromosome 16. When we calculated the ratio of fluctuations at each spretus to musculus chromosome for the 20 entire mouse chromosome pairs, we found that chromosome X was single outlier (S18 Fig) consistent with its delayed replication..

Another concern is that mapping issues may contribute to these unusual patterns. These may arise from read mapping biases from using the mm10 reference (B6) genome; Spretus-specific indels may also play a role. A quick examination of the spretus and musculus bedgraph files in the GEO submission (GSE160775_musculus_G1_bwa_110519_1.bedgraph, GSE160775_musculus_S_bwa_110519_1.bedgraph,

GSE160775_spretus_G1_bwa_110519_1.bedgraph)

shows unusual patterns of coverage; specifically, coverage varies something like 10-fold among chromosomes (within each file), but also, some chromosomes are heavily enriched in Spretus (chr4, chr11), while others are heavily enriched in Musculus (chr19). The authors should investigate and explain these unusual patterns and demonstrate expansively that mapping issues are not a concern.

The mouse Patski cells, similarly to other mouse immortalized cells, are aneuploid giving rise to enrichments of entire chromosomes the reviewer observed. We have addressed this issue by normalizing S-phase read depths to G1 read depths for S-seq replication analysis and by normalizing Mcm2-ChEC-seq dataset from cells have been treated with calcium to the read depths of sonication-fragmented DNA extracted from the same cell population that was not treated with calcium.

Discussion:

22. *Page 13:*

The authors conclude that a single MCM DH likely loads at most origins in every cell. This is OK, but I would be interested in knowing the authors’ opinion on potential biases in the MCM-ChEC-Seq method; for instance, the authors allude to an ambiguous PCR bias for shorter fragments on Page 6. Is bead-based size selection used for ChEC-Seq as per the original protocol? Is it possible that the method disproportionately excludes fragments derived from loci at which multiple, closely spaced MCM complexes are loaded? If two MCM DH complexes sit adjacent to each other, the C-termini MNase tags will potentially abut; can the MNase always cut in between? Perhaps these caveats could be discussed.

We know that MCM-ChEC does not preclude seeing multiple MCM signals, because we have no trouble seeing them in pombe and human.

23. Page 14: The 100 kb initiation zone

What is the “100 kb initiation zone in mammals”?

We mentioned these zones in the Results section:

“Previous studies have shown that at a finer scale, replication within a single early- or late-replicating zone is not uniform; for example, studies using Okazaki fragment sequencing and BrdU incorporation have shown that replication initiation within these zones is concentrated in approximately 100 kb sub- zones [35] that are typically located upstream of transcribed genes. “

To remind the reader of this, we have added the following in the discussion:

"For the same reason, we suggest that the increased MCM density along the 100 kb initiation zone in mammals reflect the contributions of footprints originating from different cells."

Methods:

*24. Methods:*

Generally, the methods could be substantially improved. Far more details are required to negate the need for the reader to consult numerous other papers.

25. Please provide version numbers for all bioinformatics tools used.

We have specified version numbers for all of the software packages that we used:

bedtools Version 2.29.1

BWA-0.7.17

samtools-1.10

picard 2.21.6

26. Methods: Tagging Mcm with MNase

“Finally, to restore the Mcm2 protein sequence to wild type, the Trp 185 residue was changed to Arg” ...what is this mutation? Please explain in more detail.

When we sequenced mouse MCM2 gene we obtained from, we noted a substitution Arg185Trp, a change

References

1. Zentner GE, Kasinathan S, Xin B, Rohs R, Henikoff S. ChEC-seq kinetics discriminates transcription factor binding sites by DNA sequence and shape in vivo. Nat Commun. 2015;6: 8733.

2. Foss EJ, Gatbonton-Schwager T, Thiesen AH, Taylor E, Soriano R, Lao U, et al. Sir2

suppresses transcription-mediated displacement of Mcm2-7 replicative helicases at the

ribosomal DNA repeats. PLOS Genetics. 2019. p. e1008138.

doi: 10.1371/journal.pgen.1008138

3. Das SP, Borrman T, Liu VWT, Yang SC-H, Bechhoefer J, Rhind N. Replication timing is regulated by the number of MCMs loaded at origins. Genome Res. 2015;25: 1886–1892.

**Reviewer #2:** Foss et al. present a new and powerful approach for mapping MCM complexes in G1 cells. Using CheC-seq, which tethers MNase to either side of the complex and releases MCM footprints upon activation, they map MCM at near nucleotide resolution in budding yeast, fission yeast, mouse and human. Based on similarities between MCM-loading patterns and replication timing profiles, and also based on their modeling, they conclude that stochastic activation of MCMs is sufficient to explain replication kinetics. This result is an important addition to a growing body of literature supporting stochastic models of replication timing. As such, it is a significant contribution to the field, and one that nicely spans yeast and vertebrates. Having said that, I am a bit disappointed in the paper, both in the way the data is analyzed and in the way the analyses are interpreted. My disappointment with the analyses is that they overly anecdotal—showing examples of data instead of doing rigorous genome-wide analyses. My disappointment with the interpretation is that the authors push two simplistic model even though there data suggests more complexed realities.

The first model is that stochastic firing of MCM explains "most differences in replication timing". Others have argued that MCM distribution is an important parameter, but it is clear other factors affect timing. The authors themselves admit as much, but in away that suggests MCM distribution is all that matters and that other factors should be discounted. However, their own best evidence for the point (**Figure S12, which should be moved to the primary figures) shows at best r = 0.72**, implying that on Chromosome IV, MCM distribution explains about half (0.72^2 = 0.52) of the variance in timing. What about the chromosomes they don't show? What is the correlation coefficient for the whole genome? Or perhaps the whole euchromatic genome? The analysis for the human genome is even more anecdotal, analyzing only 80 Mb.

We have tempered our claim that “most difference” in replication timing can be accounted for my MCM abundance. We have changed this claim in the title, abstract and elsewhere in the manuscript.

The second model is that there is only one MCM-DH loaded at any budding yeast origin. Again, the data shows that it is more complicated and the authors admit as much, but argue strongly that one is all that you need and that origins with multiple MCMs, if the actually exist, are not relevant. The motivation for their vehemence on this point is unclear, because their main point—that MCM distribution determines timing patterns—is unaffected by absolute MCM stoichiometry.

It is the authors prerogative to push their interpretations as hard as they like. However, I suspect they would convince more of their readers, and probably end up being closer to correct, if they would be a bit more nuanced in their arguments.

In any case, addressing the following points would also strengthen their case.

They use their modeling to conclude that, in budding yeast, MCM distribution explains replication timing. They should do the same thing with the actual MCM distribution. They should plot MCM signal versus replication timing at each origin.

We have included these analyses and the discussion in our revised manuscript as follows:

“In our work the relationship between MCM abundance and replication pattern in budding yeast is clearly seen in replication simulations, which assign the probability of replication initiation at specific chromosomal sites according to MCM distribution along the chromosome, capturing both the MCM abundance at individual origins and their distribution with relation to each other along the chromosome. While simulations recapitulate the replication program, if we ignore inter-origin distance and their distribution along the chromosome, and plot MCM abundance against the replication timing for each origin using the same dataset, we found no correlation between the two (S19A Fig). This is likely due to the fact that a magnitude of replication at an origin depends both on active replication at that origins and passive replication from the nearby origins which is captured in simulations but not when directly relating MCM abundance with replication timing across the origin. Another way to evaluate the relationship between MCM abundance and active replication at individual origins is to measure replication in HU, which slows down fork movement and thus measures active replication at an origin without a contribution of passive replication from the adjacent origins. Indeed, we found that replication initiation at ORI DB origins, excluding repetitive origins, as judged by accumulation of ssDNA in HU (reference), correlates with both MCM2-ChEC (R=0.65) and published MCM2-Chip datasets (R=0.57 and 0.56 [44, 45] for Das et al and Belsky et al, respectively) as shown in S19B Fig (see S2 Table for the dataset used for these calculations).”

We discuss possible reasons for somewhat higher correlation in ChEC vs MCM-ChIP datasets below in response to query 1 of reviewer 3.

They should show the distribution of single-MCM-peak versus multiple-MCM-peak origins, either as a histogram of fraction of MCM signal arising from a single DH or as a scatter plot of single-MCM% versus total MCM signal.

We thank the reviewer for this excellent suggestion. We show both the violin plots and the histograms for single vs multiple-MCM origins (Fig 3A and B). rDNA origins are excluded from the analyses as requested. As evident from these figures, single MCM-peaks have overall higher abundance, and have skewed distribution toward higher MCM abundance in distribution plots compared to multiple MCM peaks. These new figure simplify the presentation of the results allowing us to avoid the more convoluted arguments such as those in the original manuscript, to which the reviewer objected below.

Their estimation that origins with multiple MCM peaks comprise only 25% of the total MCM signal is skewed by inclusion of the two rDNA origins, which (as they are repeated about 100 times) comprise 35% of the total signal. Excluding them (or including them at normalized levels), the one MCM/multiple MCM ratio is about 60/40.

The reviewer is correct that the rDNA origins represent 35% of the signal, and therefore skew the ratio. We have therefore excluded rDNA from this analysis and obtained the ratio of 62/38, which is similar to what the reviewer calculated.

They make a strong case that less than 1000 MCMs (500 MCM-DHs) are loaded each G1 in budding yeast, yet numerous studies (e.g., [https://www.ncbi.nlm.nih.gov/pubmed/29361465](https://urldefense.proofpoint.com/v2/url?u=https-3A__www.ncbi.nlm.nih.gov_pubmed_29361465&d=DwQGaQ&c=eRAMFD45gAfqt84VtBcfhQ&r=v2OLxwF1QhJd633WJXHa80Y4xpeZHjONCdo5Te6LOtU&m=85-HvZnq_GbEAg0Kqx26y4Dey03kBqcPo36PKVGlbxE&s=ouDKjEYxkftodeYK3siJDv0i8eY4-EqG_gMwq8G-Kh8&e=) ) have estimated that there are 3000 to 5000 MCMs per cell, most of them loaded during G1. The authors need to address this discrepancy.

The abundance of MCM components has been addressed in multiple studies. These studies consistently show that Mcm2 appears to be the least abundant component, measured at 2700 copies per cell in the study mentioned by the reviewer, but was somewhat lower in the other studies (e.g. 1690 copies per cell in PMID: 14562106). These Mcm2 could assemble at best 850-1350 DH. Considering that not all MCMs are loaded, this estimate is not far off ~500 DH the reviewer mentions.

They say that they "used the same approach" for pombe as cerevisiae, but the method of synchronization, HU instead of mating pheromone, is substantially different, and should be noted and explained. The fact that the HU and G1 data sets look similar also needs explaining because, by their model, the efficient origins—such as ARS1103, which fires is >80% of HU-arrested cells—should be almost completely depleted of MCM-DHs is the HU data set.

The reason we included HU data for cereviseae is to determine whether the diffuse appearance of MCMs in pombe, which were arrested by HU, could be caused by movements of MCM with the replication fork when cells are not exclusively in G1. For this purpose, we added HU to a log phase cerevisiae cultures 60 minutes and carried out ChEC, mimicking the HU arrest in pombe which is also carried out from a log culture. Single MCM footprint-origins, which comprise a large fraction of cerevisiae origins, provide a sensitive assay to determining whether origin that fire in HU change the footprint appearance. Given that single-footprint origins remain sharp, we conclude that our assay does captures preferentially MCM DHs over MCM single hexamers that move together with the replication fork. Possible reasons for this include that MCM DHs, as compared to MCM single hexamers, are more likely to release a small fragment that can be amplified, and that the fragments released by each of the DH as they travel in the opposite direction are too large to be amplified. The preference for capturing DHs is such that even if only 20% remain unfired, they can still be easily detected by ChEC. Indeed, we have observed that the relative abundance of MCM of highly active “early origins” as compared to “late origins” is diminished in HU as expected.

They should site the literature that describes stochastic models similar to theirs and likewise concludes that stochastic activation of MCM complexes is sufficient to explain replication timing patterns, in particular d'Moura [https://pubmed.ncbi.nlm.nih.gov/20457753](https://urldefense.proofpoint.com/v2/url?u=https-3A__pubmed.ncbi.nlm.nih.gov_20457753&d=DwQGaQ&c=eRAMFD45gAfqt84VtBcfhQ&r=v2OLxwF1QhJd633WJXHa80Y4xpeZHjONCdo5Te6LOtU&m=85-HvZnq_GbEAg0Kqx26y4Dey03kBqcPo36PKVGlbxE&s=Xb00MNhnkTVJtIc5ZUK17Bq6foaCB6qj4HAN2OMf6Wc&e=), Yang [https://www.ncbi.nlm.nih.gov/pubmed/20739926](https://urldefense.proofpoint.com/v2/url?u=https-3A__www.ncbi.nlm.nih.gov_pubmed_20739926&d=DwQGaQ&c=eRAMFD45gAfqt84VtBcfhQ&r=v2OLxwF1QhJd633WJXHa80Y4xpeZHjONCdo5Te6LOtU&m=85-HvZnq_GbEAg0Kqx26y4Dey03kBqcPo36PKVGlbxE&s=KAX9nbEBIKNYTqnp1fzLMWmAtJQuXMjSARhmjDBwmHo&e=) and Gindin [https://www.ncbi.nlm.nih.gov/pubmed/24682507](https://urldefense.proofpoint.com/v2/url?u=https-3A__www.ncbi.nlm.nih.gov_pubmed_24682507&d=DwQGaQ&c=eRAMFD45gAfqt84VtBcfhQ&r=v2OLxwF1QhJd633WJXHa80Y4xpeZHjONCdo5Te6LOtU&m=85-HvZnq_GbEAg0Kqx26y4Dey03kBqcPo36PKVGlbxE&s=PKm87vC1XvvL3e8cZvtyLxVD8v_m_agx70wqmlkZGVY&e=) .

We have included these as well as several other studies that have proposed stochastic firing of origins in the introduction as follows:

“Models for replication timing that emphasize control at the level of helicase loading, with subsequent firing of origins being largely stochastic, have been proposed by several groups on the basis of single-molecule and genome-wide analyses [31-40]. Our observation that much of the replication timing program can be derived from the pattern of MCM loading prior to S phase lends powerful support to this pleasingly simple and tractable view of replication timing in eukaryotes.”

I would be very interested to know how efficient the ChEC cleavage is. If it is efficient, it would strengthen their claim that they are mapping every loaded MCM. However, if it is inefficient, it is possible that they are detecting a biased subset of MCMs. A simple genomic Southern blot before and after calcium treatment would suffice.

We than the reviewer for this excellent suggestion. Southern blot and q PRC analysis show that MCM-MNase digestion is both rapid and highly efficient. We present these new data as follows:

“To determine the speed and efficiency of Mcm2-MNase digestion and to estimate the fraction of origins at which MCM2-7 had been loaded, we analyzed a time course of MNase digestion at ARS1103 by Southern blot: DNA was extracted from permeabilized cells carrying Mcm2-MNase after calcium treatment , digested with Xmn1, which releases a 4.3 kb genomic fragment, and probed with a 261 base pair 3’ terminal probe extending up to 17 base pairs to the right of the ACS in ARS1103, as **depicted in Fig 3 A.** We observed a rapid reduction in the abundance of the intact 4.3 bp fragment (~40% reduction within 15 seconds) which proceeded to up to ~90% completion at 5 minutes and without major further reduction at 10 minutes in **calcium (Fig 3A and B).** Loss of abundance of 4.3 kb fragment was coupled with the appearance of an approximately 260 bp fragment corresponding to the fragment released by the MNase digestion between the footprints of MCM and right nucleosome. Similar dynamics of MNase digestion was obtained with tagged Mcm2, Mcm4 and Mcm6 using qPCR (Fig 3B) with primers that span the 194 bp region between the footprints of the left and the right nucleosomes as depicted in Fig 3A.. These results demonstrate that at ARS1103, Mmc2-7 has been loaded in at least 90% of the cells, and, furthermore, that these loaded origins contain a single MCM DH.

They should include in a supplemental table information about the size and quality of their sequence data sets.

We have added a table.

Minor Issues:

ACS is 17 bp, not 11 bp. Both cited papers show a 17 bp ASC.

We have changed the text accordingly.

On page 16, the phrase "no difference" is a bit strong; "no significant difference" would be more accurate, and significant should be defined.

We have changed the text accordingly.

The red arrow in Figure 1B is presumably the ACS. If so, it should be so labeled.

We have labeled the figure accordingly.

The genomic coordinates of the regions shown in Figure 2A should be provided.

We have provided genomic coordinates.

The yeast replication profile data in Figure 2A should be cited.ERIC WILL DO.

The y axes of the bottom rows of Figure 2A should be properly labeled. The y values of these graphs are almost certainly not simply read depth.

The reviewer is right, as not all the studies were carried out by deep sequencing. We have changed the labeling to “Relative replication”.

The library read-size distribution for all species should be plotted on the same graph for easier comparison.

The requested plot is presented in S12 Fig.

Reporting a p value of 5.3x10E-46, and the implication that there is a meaningful difference between 10E-46 and 10E-63, is silly. p < 10E-10 would suffice and model a more realistic approach to statistics.

We have changed the labeling accordingly.

The authors claim that it "is clear ... the number of early- and late-replicating regions on a single chromosome is similar". Absent quantitative, genome-wide analysis, it is not clear. Furthermore, it is immaterial to the paper and should be removed.

We have removed this section as suggested.

**Reviewer #3:** Review of Foss, Sripathy, et al. (Bedalov) “Chromosomal MCM2-7 distribution is the primary driver of the genome replication program in species from yeast to humans”

MCM complex binding to chromosomes has been assessed in multiple independent experiments over the past decades in budding yeast using genome-scale Chip-chip and MCM ChIPSeq approaches. In yeast, distinct MCM ChIP signals corresponding to known origins of replication can be easily detected and closely overlap ORC binding sites, consistent with the finding that in this organism origins are specified by discrete DNA regions of less than 200 bp. In contrast, in other organisms, particular metazoans including human cells, MCM complex binding sites have been much more difficult to map precisely. In Drosophila cells, the MacAlpine lab showed that the cyclin E/Cdk2 promotes multiple MCM complex loading events and that the loaded MCM complexes are then distributed over chromosomes at least in part by active transcription (Powell et al, EMBO J 2015). Thus every single cell in a clonal population potentially possesses a distinct distribution of MCM complexes, making discrete MCM complex sites virtually impossible to map. However, in the absence of E/Cdk2, 10-fold fewer MCM complexes are loaded and these are localized to known ORC sites, as occurs in yeast. Regardless, the relationship between MCM complex levels, distribution and replication time has been challenging to tease out in eukaryotes, including yeast. Moreover, in yeast there is strong published evidence that the localization of limiting S-phase factors (i.e. the ability of these S-phase factors to access licensed origins) plays a primary role in controlling origin activation time, and thus the replication timing ‘program’, which runs counter to the major conclusion that the authors of this study are making, at least based on the title and abstract sections of their paper (they do acknowledge limitations or exceptions to these conclusions in their Discussion).

We have moderated our claim that MCM loading is the primary driver of the replication program throughout the text as explained above and changed the title of the manuscript accordingly.

In this study, the authors adapt a relatively new genome-scale protein mapping procedure developed in by the Henikoff lab called ChEC (chromatin endogenous cleavage) to map MCM binding sites in budding yeast, fission yeast and mammalian cells. In these experiments individual Mcm subunits are fused to MNase; cells are isolated between MG1, the period where MCM complexes are loaded, and permeabilzed. The addition of CaCl2 activates the MNase which cleaves DNA where MCM complexes are bound. The released fragments sequenced and mapped to the genome. Fragments of ~ 60 bp, the approximate region protected by an MCM complex, are interpreted as loaded MCM complexes.

Using this method across the cells of different species, the authors make the general conclusion that, despite the differences in molecular determinants of origins and ORC binding mechanisms (e.g. a discrete conserved DNA sequence is used by budding yeast ORC, an AT-rich region and a species specific AT-hook on one Orc subunit is used by S.pombe ORC, no simple sequence rule is used by mammalian ORC, but it appears to often accumulate in “open chromatin” regions) the distribution of loaded MCM complexes determines/correlates with the replication timing pattern of the genome, with regions containing higher densities of MCM signals replicating earlier and those containing lower densities replicating later. Thus they conclude that the distribution of licensed origins is a primary driver of the genome replication program.

While there are a lot of interesting and useful new data in this study, no single line of inquiry was developed sufficiently enough to make a definitive new conclusion that advances or clarified the current mechanistic status of the field. In particular, in budding yeast and other model organisms, it is clear that the factors that activate MCM complexes, converting them to active bidirectional helicases and causing origin firing, are present in limiting amounts relative to loaded MCM complexes (Mantiero (Zegerman), EMBO J 2011; Tanaka (Araki) Current Biology, 2011). Over-expression of these factors causes late regions to replicate earlier and leads to faster cell cycles and viability (in yeast) or developmental defects in metazoans (e.g. Collart (Zegerman) Science 2013). In yeast, where precise S-phase origin activation times can be assigned to individual origins, origins that are normally activated late in S-phase are now activated earlier. Thus limiting concentrations of MCM complex activation factors relative to the numbers of loaded MCM complexes can, to a substantial extent, explain origin activation time and the replication timing “program” of yeast chromosomes. In further support of this model early firing of centromere proximal origins is achieved by the ability of a kintechore complex, Ctf19, to recruit Dbf4, the regulatory subunit of Cdc7, a kinase, and a one of the limiting S-phase origin activation factors (Natsume Mol Cell 2013). The MCM complex is a direct substrate of this kinase, this its recruitment causes an origin to fire. In the absence of this recruitment mechanism, replication of yeast centromeric regions is specifically delayed. Another group of early origins use the forkhead transcription factors to recruit Dbf4 and activate the MCM complex (Fang, (Pasero, Lou) Genes Dev 2017). Thus it is clear that limiting S-phase factors can explain the stochastic behavior of origins to a large extent with early origins acting early because they possess mechanisms that increase the probability that they can recruit limiting S-phase factors).

Obviously, because MCM complex is the substrate for limiting S-phase activation factors, loaded MCM complexes must be present. The authors are correct in the assertion that fewer studies have addressed the level of MCM complex loading and, in theory, later replicating regions could result from either 1, a much lower abundance of MCM complexes or a lack of them all together or 2, the presence of MCM complexes that are considerably less competitive for recruiting the limiting S-phase factors. The question is whether the distribution of MCM complexes versus the accessibility of these loaded complexes to S-phase activation factors is the major determinant of replication timing patterns. The extant data, particularly from yeast, is that distribution of S-phase factors are a primary determinant. Thus, based on the title and the abstract of the current study, the authors conclusion that MCM complex abundance is the primary determinant of the replication timing program runs counter to the current view. Perhaps their use of a novel method to map MCM complexes is revealing something that has been missed by MCM ChIPSeq approaches, the primary method used to date to map licensed origins in vivo. Here are three detailed concerns:

**1, In yeast, where precise efficiency values (Origin Efficiency Metric)** and precise origin replication times have been mapped by a multitude of approaches, there is compelling evidence that that altering the concentrations of Dbf4 through discrete protein-protein interaction mechanisms, can substantially alter the activation timing of individual origins and thus the replication timing “program”. In addition, few studies have generated c evidence for a strong correlation between the levels of MCM complex loading (measured by MCM ChIPSeq) and the time at which an origin is activated. In the one published study that reported a correlation between the level of MCM complex signals and timing, the correlation is actually quite weak and not predictive, clearly suggesting that other factors contribute to determining an origin’s replication time (Das (Rhind) Genome Res, 2015).

Given that the authors use a new approach to assess loaded MCM complexes, additional analyses of the S. cerevisiae data should be performed to produce a clear picture of how the data from the MCM CheC method used here compares to recently published MCM ChIPSeq data and lots of other strong published data about yeast origins. The field knows so much about yeast origins that it makes sense to challenge the MCM CheC method more deeply in this model. In particular, the published MCM ChIPSeq data sets do not show a strong, highly predictive correlation between origin activation time in S-phase and the level of MCM complex loading at an origin. And while a weak correlation is reported in one study, it also quite clear that some late origins have MCM ChIP Seq signals that are similar to those of early origins. Therefore, the authors should address:

How well do the MCM CheC signals measured at individual origins in this study correlate with measured origin activation times?

How well do the relative MCM CheC signals compare to the relative MCM ChIPSeq signals from published studies?

We compared the relative MCM ChEC-seq signals at 821 OriDb origins with MCM Chip-seq signals from Das et al 2015 and Belsky and al 2016 and found correlations of r=0.75 and 0.70, respectively. Not surprisingly, the correlation of MCM signals between two published MCM ChIP-seq studies was somewhat higher at 0.83.

In other words, is MCM CheC method allowing you to detect/assess/infer relative differences in MCM complex levels at yeast origins that are lost by MCM ChIPSeq data? And, if so, how do you know whether the lower signals at late origins in your MCM CheC experiments are the result of less MCM being loaded OR the result of reduced access of the underlying DNA to MNase cleavage?

As noted above in the response to reviewer 2, we found that replication initiation at ORI DB origins, excluding repetitive regions, as judged by ssDNA in HU strain correlates with both MCM2-ChEC (0.65) and published MCM2-Chip datasets (0.57 and 0.56 for Das et al and Belsky et al, respectively). Somewhat higher correlation with ChEC as compared to Chip could be due to, as the reviewer suggested, more accurate measurement of MCM abundance or capturing differences in chromatin accessibility by ChEC that are not reflected in ChIP. It is difficult to determine which of the two might be is responsible for higher correlations. To try to address this question, we calculated the fraction of reads that map to heterochromatic DNA regions (subtelomeric regions and rDNA) by two methods, reasoning that heterochromatic origins might be more resistant to ChEC and thus under-represented compared to ChIP.  However, we found that the heterochromatic origins do not differ significantly between ChEC-seq (43%) vs and ChIP-seq (41% and 38% for Das et al and Belsky et al, respectively), suggesting that heterochromatin is not a major impediment to ChEC. Similarly, we found that Mcm2-ChEC signal at inactive, heterochromatic X-chromosome is similar to the Mcm2-ChEC signal at the active X.

Given the strong conclusion the authors want to make using a new less scrutinized method that seems to be producing conclusions that run counter to published thinking in the field, such scrutiny is advisable. This information could then help readers better interpret the MCM CheC signals in eukaryotic cell types where MCM ChIPSeq has been relatively uninformative or confusing.

2, To a certain extent, it is not at all unexpected that at some level the abundance of licensed origins (loaded MCM complexes) should correlate with the replication timing program, for the simple reason that the MCM complex is the substrate for the S-phase factors and is essential for initiation. Thus, a chromosomal region lacking MCM complexes, regardless of its ability to recruit S-phase activation factors, will replicate later than neighboring regions with an abundance of loaded MCM complexes by the laws of physics. Indeed, a study from the Struhl lab (Miotto, PNAS, 2016) concludes that replication timing in human cells is determined largely by the density of ORC sites and the stochastic activation of origins. Given that ORC’s role is to load MCMs it is likely that ORC density must correlate with MCM complex density. Perhaps the study would be improved by being more explicit about whether the authors are thinking about the activation time of individual origins (loaded MCM complexes) or the relative replication time of a chromosomal region (and hence the abundance of detectable MCM complexes over a defined region of the genome). The distinct point nature of yeast origins makes it challenging to compare the bases for replication timing programs in yeast and other organisms--the current terminology/language is limiting/

We have inserted the folllowing two sentences, marked in bold, to incorporate this insightful suggestion:

To determine whether chromosomal MCM density in G1 along the chromosome is sufficient to drive replication timing pattern in budding yeast, we modeled Mcm2-7 binding and DNA replication *in silico*, assuming that in each cell DNA replication initiates at random from sites bound by Mcm2-7 in that cell. **We reasoned that a computer simulation of replication would be particularly helpful because, while we assume that replication timing patterns across large chromosomal regions reflect the integrated activation times of the individual origins in those regions, the time at which a particular origin is replicated does not necessarily reflect the time at which that origin fired. For example, an origin that is licensed in only a small fraction of cells may complete replication relatively early in S phase due to its passive replication by a nearby origin that is licensed in a large fraction of cells.** The stochastic origin firing model that we developed, which is similar to those used by others…

3, Finally in this study under consideration, the authors report clear exceptions to the major conclusion “rule” that they assert. For example, they conclude that the Xa and Xi chromosomes, though they replicate at different times during S-phase, show similar patterns and abundance of MCM CheC signals, and thus that MCM loading cannot explain their replication timing differences. This result is interesting, but it seems like a major “exception” given that heterochromatin is quite abundant in metazoans.

We acknowledged that heterochromatin might be a major exception to this rule.

In summary, the effort to apply a new approach to mapping MCM complexes in eukaryotic cells is admirable and the data are very likely revealing new facets about MCM complex behavior on chromosomes and its relationship to origin activity. However, the assay was used across multiple cell types without sufficiently rigorous challenging in yeast, the best developed model for origins. Thus neither the method or any single conceptual issue was sufficiently delved into to contribute a clear solid advance to the field. (Mcm subunits are not equivalent to transcription factors in dynamics and the assay was developed with those factors in mind) Thus, despite the title, the major new conclusions that will alter or challenge the current thinking in the field are unclear.
