# Supplementary material for: Chromosomal Mcm2-7 distribution and the genome replication program in species from yeast to humans
Source: PLoS Genet. 2021 Sep 2;17(9):e1009714. doi: 10.1371/journal.pgen.1009714 (PMC8443269; doi:10.1371/journal.pgen.1009714)
Supplement: Attachment — Submitted filename: ResponseToReviwers_Foss_June_2021.docx [file pgen.1009714.s038.docx]

Dear Dr Bedalov,

Thank you very much for submitting your revised Research Article entitled 'Chromosomal Mcm2-7 distribution and the genome replication program in species from yeast to humans' to PLOS Genetics.

The manuscript was fully evaluated at the editorial level and by independent peer reviewers. The reviewers appreciated the attention to an important problem. Two of the reviewers were satisfied with the revisions, but one reviewer raised some substantial concerns about the current manuscript, and I (ML) also have remaining substantial concerns. Based on the reviews, we will not be able to accept this version of the manuscript, but we would be willing to review a much-revised version. We cannot, of course, promise publication at that time.

The reviews are included below and/or attached; my comments are immediately below.

1.  Of great concern is the continued absence of methods information sufficient to evaluate the data, let alone repeat it. This ranges from the trivial (formula for pombe media, growth media used for mammalian cells) to the profound (complete absence of methods for MNase-alone controls). The standard for completeness of methods should be that sufficient information is included to allow a third party to accurately replicate the experiments performed. None of the current protocols are sufficiently described, and given that this is an online journal without word limits, there is no reason not to be complete. If anything, one should err on the side of loquaciousness. Please be aware that unless full methods are provided, it will not be possible to evaluate remaining scientific concerns (for example, about the validity of MNase-only controls).

We have now provided detailed experimental protocols regarding the media, exogenous MNA-seq experiments and have expanded the description of our ChEC-seq protocol. We have also provided a more detailed information about the computational methods we have used for generating MCM heat maps. (A detailed description with code showing generation of such a plot from fastq sequence files is provided in the source files for Figure 1.)

2. Of similar concern is the continued unavailability of underlying data. I could not find a **data availability statement**, the data underlying many of the graphs and figures are not provided, and it appears that data for the MNase controls is absent. Please refer to the PLOS data availability guidance ([journals.plos.org/plosgenetics/s/data-availability](http://journals.plos.org/plosgenetics/s/data-availability), see also below) for what is required. Briefly, numerical data underlyling every graph must be available. Many authors find it convenient to include this in a single Excel workbook with separate worksheets for each figure panel, but there are alternatives listed in the guidance. Again, if data are not available then it will not be possible to evaluate remaining scientific concerns.

The Data Availability statement is required as part of general information abut the manuscript during submission. We have stated that all our data are fully available without restriction and we have provided the GEO accession number.

We apologize for failing to include our free MNase dataset in the GEO database, which was an oversight that we have now corrected.

Lastly, we provide the source files for all the graphs for both figures and supplemental figures. These files have been uploaded as 12 clearly labeled ZIP-compressed directories containing .txt files in S1 Source Files.

3. Actual numerical values and units for graphs and figures. In my opinion, most of the graphs and heat maps in the current manuscript are no better than cartoons. Please include, in every graph, a Y axis with real numbers and units that are defined explicitly in either the methods (if a term such as "relative abundance" is used repeatedly) or in the figure legend. Please include in every heatmap a spectrum bar with real numbers and units. "Avoiding confusion" is not a valid reason to omit such essential information.

We offer our sincere apologies, both to the reviewers and to the editors, for not sufficiently complying with the requests for scales in the heat maps. Our intent was to simplify rather than to evade, but we realize that we erred in this regard, and that it is our obligation to provide the reader with all numbers that may be useful in evaluating our conclusions. We believe that we are now doing so, and we describe the issue we were grappling with and how we have addressed it below.

The heat maps serve as images of MCM helicase binding at origins that allow the reader to evaluate our interpretations of the data, such as our conclusion that replication origins in *S. cerevisiae* are typically licensed by loading of a single MCM helicase complex. Many of these images are only semi-quantitative, because, although the color scale is linear, it is often capped such that everything above a certain cutoff is assigned the lightest color. We do this not to hide anything, but rather to enhance the lower intensity signals. This enhancement, if anything, tends to hurt our argument that licensing occurs by loading of a single MCM complex, because lower intensity signals are selectively enhanced. Nonetheless, we do it because it adds to the qualitative features that can be discerned in these images. For example, the heat map immediately below, which is taken from Figure 1B, is capped, which allows one to see the nucleosomes (right nucleosome is indicated with white arrow), whereas the heat map below that it is not capped and is, as a consequence, less informative.

However, immediately adjacent to the heat maps in Figure 1B, and immediately below each of the 343 S. cerevisiae origins in S4-Figure, we provide read depths distribution graphs and their source files in the same ~3 kb genomic area around the origins. These data are never "capped", since they are not limited by the dynamic range available in a color-based scale. In providing read depth quantitation for Figures 1B and C, there are two potential sources of confusion that we had hoped to avoid by labeling our figure as we did: First, there is no reason for the total number of reads to be the same for the three data sets represented, and, as it happens, they are not: We have 15.5 million, 4.2 million, and 6.3 million reads for our MCM2-ChEC, MCM4-ChEC and MCM6-ChEC data sets, respectively. Second, the read depth numbers for each heat map will not match those in the read distribution to the right. For example, the "uncapped" heat map above maxes out at 3000, whereas the corresponding plot will max out at 20,000. This is because the *heat map quantifies read depths at specific sites for library fragments of one specific read length*, whereas *the read distribution plot on the right quantifies the sum of read depths over a range of library fragment lengths*, e.g. fragments between 50-100 bp and those between 150-200 bp as in Fig 1C. There is nothing wrong with this, but it is confusing, and the confusion stems from using absolute numbers for sequencing read depths when relative read depths are both clearer and more relevant. We have addressed these issues as follows: (1) We have clarified how the heat maps were generated in S1 Source File for Figure 1B. (2) In the heat maps in figures 1B, 2A, we have provided color keys on the heat maps themselves, and in cases where the highest value has been capped, we indicate that as the lower end (e.g. "> 400", as shown above). **These read depths refer to a specific base pair at a specific fragment size, since these are three-dimensional plots (x, y, and color intensity). In addition to the color key, we provide files with matrices of the read depths that were used to generate the pictures.** (3) In figures S4, and S8, which each contain from 12 to 343 heat maps, we have listed the ranges for each heat map in the figure legends. (4) In read distribution plots, as in Fig 1C and figures 4, S4 and S5, we have labeled the y axis with "relative read depth" and a scale that goes from 0 to 1, and we specify in the legends that these scales are linear, uncapped, and start with 0. Also, absolute read depth values for each plot are now provided in the figure legends. Lastly, the numerical source file for each figures are provided as supplemental data: S1 Source files.

For Figure 1A, we have listed the read depths in the figure legend.

4. Minor requests. a) In Figure 7 and the accompanying supplementary figure, it is very difficult to discern the transition from red to blue dots that are meant to mark initiation and termination zones. Please indicate the location of each of these--perhaps differently colored vertical arrows on the X axis?--using criteria that can be defined. It is not at all clear, for example, that Mcm peaks really correspond to measured initiation zones. b) please carefully proof references for proper species and gene name italicization, title capitalization, etc.

We have now marked the centers of initiation and termination zones with blue and orange tick marks on the X-axis in Figure 7, using the coordinates as calculated in a study by Petryk et al. [1] and provided to us by Olivier Hyrien. We have also remade the violin plot of MCM abundance in Figure 7B using these coordinates.

Should you decide to revise the manuscript for further consideration here, your revisions should address the specific points made by each reviewer. We will also require a detailed list of your responses to the review comments and a description of the changes you have made in the manuscript.

If you decide to revise the manuscript for further consideration at PLOS Genetics, please aim to resubmit within the next 60 days, unless it will take extra time to address the concerns of the reviewers, in which case we would appreciate an expected resubmission date by email to [plosgenetics@plos.org](mailto:plosgenetics@plos.org).

If present, accompanying reviewer attachments are included with this email; please notify the journal office if any appear to be missing. They will also be available for download from the link below. You can use this link to log into the system when you are ready to submit a revised version, having first consulted our [Submission Checklist](https://urldefense.proofpoint.com/v2/url?u=http-3A__journals.plos.org_plosgenetics_s_submit-2Dnow-23loc-2Dsubmission-2Dchecklist&d=DwMGaQ&c=eRAMFD45gAfqt84VtBcfhQ&r=v2OLxwF1QhJd633WJXHa80Y4xpeZHjONCdo5Te6LOtU&m=nUAY-SbFKnEb3rRVZcYJxGW6i0yc7hq6SPx-aVDGwbo&s=0HXhgBVZN3xg92JYDQelkWR-GRSEmYtDjlYV9uoQbR8&e=).

To enhance the reproducibility of your results, we recommend that you deposit your laboratory protocols in [protocols.io](http://protocols.io/), where a protocol can be assigned its own identifier (DOI) such that it can be cited independently in the future. Additionally, PLOS ONE offers an option to publish peer-reviewed clinical study protocols. Read more information on sharing protocols at [https://plos.org/protocols?utm_medium=editorial-email&utm_source=authorletters&utm_campaign=protocols](https://urldefense.proofpoint.com/v2/url?u=https-3A__plos.org_protocols-3Futm-5Fmedium-3Deditorial-2Demail-26utm-5Fsource-3Dauthorletters-26utm-5Fcampaign-3Dprotocols&d=DwMGaQ&c=eRAMFD45gAfqt84VtBcfhQ&r=v2OLxwF1QhJd633WJXHa80Y4xpeZHjONCdo5Te6LOtU&m=nUAY-SbFKnEb3rRVZcYJxGW6i0yc7hq6SPx-aVDGwbo&s=70wULkqvhH3fLMni9xrXMgDffJrJYEz-b-eZ8TjMYNw&e=)

Please be aware that our [data availability policy](https://urldefense.proofpoint.com/v2/url?u=http-3A__journals.plos.org_plosgenetics_s_data-2Davailability&d=DwMGaQ&c=eRAMFD45gAfqt84VtBcfhQ&r=v2OLxwF1QhJd633WJXHa80Y4xpeZHjONCdo5Te6LOtU&m=nUAY-SbFKnEb3rRVZcYJxGW6i0yc7hq6SPx-aVDGwbo&s=cRMqtqrpWC09Lf5LQUw06jkPkQykuY9fs1xXLaJjPMA&e=) requires that all numerical data underlying graphs or summary statistics are included with the submission, and you will need to provide this upon resubmission if not already present. In addition, we do not permit the inclusion of phrases such as "data not shown" or "unpublished results" in manuscripts. All points should be backed up by data provided with the submission.

While revising your submission, please upload your figure files to the [Preflight Analysis and Conversion Engine](https://urldefense.proofpoint.com/v2/url?u=http-3A__pace.apexcovantage.com_&d=DwMGaQ&c=eRAMFD45gAfqt84VtBcfhQ&r=v2OLxwF1QhJd633WJXHa80Y4xpeZHjONCdo5Te6LOtU&m=nUAY-SbFKnEb3rRVZcYJxGW6i0yc7hq6SPx-aVDGwbo&s=E1ff__xXYAIqzwnn1vnNx3hstEerMOPvYsXqZpqdPSw&e=) (PACE) digital diagnostic tool.  PACE helps ensure that figures meet PLOS requirements. To use PACE, you must first register as a user. Then, login and navigate to the UPLOAD tab, where you will find detailed instructions on how to use the tool. If you encounter any issues or have any questions when using PACE, please email us at [figures@plos.org](mailto:figures@plos.org).

PLOS has incorporated [Similarity Check](https://urldefense.proofpoint.com/v2/url?u=http-3A__www.crossref.org_crosscheck.html&d=DwMGaQ&c=eRAMFD45gAfqt84VtBcfhQ&r=v2OLxwF1QhJd633WJXHa80Y4xpeZHjONCdo5Te6LOtU&m=nUAY-SbFKnEb3rRVZcYJxGW6i0yc7hq6SPx-aVDGwbo&s=5dhSIDeGqcZnVTseAmap00nr7m9FxfFfen6bw24yTZM&e=), powered by iThenticate, into its journal-wide submission system in order to screen submitted content for originality before publication. Each PLOS journal undertakes screening on a proportion of submitted articles. You will be contacted if needed following the screening process.

To resubmit, use the link below and 'Revise Submission' in the 'Submissions Needing Revision' folder.


We are sorry that we cannot be more positive about your manuscript at this stage. Please do not hesitate to contact us if you have any concerns or questions.
Yours sincerely,

Michael Lichten, Ph.D.
Associate Editor

Gregory P. Copenhaver
Editor-in-Chief

Review of PGENETICS-D-20-01578_R1:

First, I would like to mirror the thoughts of the other reviewers to state that the experiments presented in this work certainly have the potential to offer new insights into MCM localization and activity. However, the authors continue to lean into conclusions not supported by the data, have not addressed many points raised by reviewers and many of the new analyses and datasets do not satisfactorily address the concerns raised during peer review. In many ways, the authors’ response has been quite frustrating.

Major critiques:

1. The inclusion of control and replicate data is inconsistent and opaque (see below).

We provide the data we used for making each figure in the supplement S1 Source Files, including both raw reads and normalized reads for MCM2-ChEC for the 343 budding yeast origins in S4Fig. Below we discuss each of these points in detail.

2. Threaded through the manuscript is the assertion that the multiplicity of MCM binding

can be inferred using ChEC-Seq. The authors highlight this as an advantage of ChEC-Seq over ChIP-Seq, and assert that it allows them to demonstrate that one MCM binds per origin in S. cerevisiae. Nonetheless, the authors agree in response #7 that this type of inference is not possible and I see no other evidence to back up this claim. They have ignored the suggestion to compare ChEC-Seq to origins previously found to bind multiple MCMs and continue to assert that their findings contradict this previous work.

We discuss the multiplicity of MCM binding in point 7 below and the ChEC analysis for ARS416, previously reported to bind multiple MCM in a plasmid-purification based assay, in point 11.

3. The authors have not responded to many of my suggestions (and those of the otherreviewers). In many cases, they do so without providing a rationale.

We have done our best to address all the suggestions, as elaborated on below.

4. The analyses of the data from pombe, mouse and human have only marginally improved

and remain unconvincing. Most frustrating is that while a key aspect of this method is the

ability to resolve beyond the limits of techniques such as ChIP-Seq, the authors have not

performed any high resolution analyses to demonstrate that the method worked in these

other organisms.

In both pombe and human, we compared our results with MCM2-ChEC to data sets whose quality in terms of the combination of resolution and sensitivity we believe to be as high or higher than that of any other data sets aimed at identifying the regions at which DNA replication initiates in these two organisms. These techniques were Pu-seq in pombe and Okazaki-seq in humans [1, 2]. The reviewer mentions three "high resolution" techniques that we neglected to use as standards against which to evaluate our results in HeLa cells: (1) Orc-ChIP [3]; (2) ini-seq [4]; and (3) SNS [5]. We have now compared our results with MCM2-ChEC in HeLa cells with the peaks of signals in these data sets, using coordinates for the peaks that were determined by the authors of the respective studies. Two sample t-tests, using "1 kb bins that overlap peaks" as one group and "1 kb bins immediately adjacent to bins that overlap peaks" as the second group yielded two-tailed p values <10e-20 in all three cases. We have added this information in the text and added tick marks showing locations of peaks in these 3 additional data sets in Figures 7A and S14A-F. It is worth noting that, although the three sets of "high resolution" peaks overlap in the multi-kb regions that they identify as replication initiation zones, the individual peaks identified by each technique are frequently non-overlapping.

5. The authors have not clarified what this study adds to our body of knowledge.

There is controversy in the replication field about the relative importance of licensing versus firing in determining replication timing during S phase. We have applied a novel technique in 4 organisms and concluded that our results emphasize the importance of licensing over firing. We state this conclusion in the abstract. Like every other study that has addressed this issue, our study will not put the issue to rest, but it is a substantial body of work that can help researchers in the field form their own conclusions.

There is also active controversy within the camp that favors licensing over firing about whether the efficiency of an origin depends more on (1) the number of MCM complexes that are loaded at that origin in individual cells, or (2) the fraction of the cells in the population that contain a single MCM complex at that origin. We made use of the unprecedented resolution of the ChEC technique to present a powerful argument for the latter paradigm in budding yeast, and we suggested that, for reasons of economy, we favor the notion that this model is likely to be true not just in budding yeast but also in fission yeast and mammalian cells. We believe these to be substantial contributions to our body of knowledge that will be of broad interest to readers of PLoS Genetics.

Detailed critiques:

Controls:

Although the authors have added MNase controls to some figures, several things remain

unclear:

1. Did the authors themselves perform the control experiments in conditions matched to those of the ChEC-Seq experiments? Several recent papers have debated the merits of ChEC-Seq [1–4], and it seems clear that the MNase controls should be closely matched to the experiment performed[5]. It is not clear from the details provided by the authors if that is the case here. For HeLa cells, the authors used published data. It seems unlikely that this is a sufficiently well matched control.

We apologize for failing to provide this important information. We performed the control exogenous MNase experiments for budding and fission yeast under conditions that match those that were used for ChEC experiments: namely alpha factor-arrested cells in budding yeast, and HU-arrested cells in S. pombe. Detailed protocols for the exogenous MNase experiment is now provided in the method section. The sequencing datasets have been deposited in GEO. For the published exogenous MNase datasets for HeLa, the exposure to MNase matches closely those in out yeast exogenous MNase experiments.

2. The authors claim that the pattern of cleavage using MCM-ChEC-Seq is notably different to the MNase controls, however in the single figure that shows the per-locus data side-by-side (in cerevisiae, Figure S5), the patterning is remarkably similar at some loci. Indeed, the biggest difference in some cases appears to be the unusually low background in the ChEC-Seq experiments. Since the authors have not complied with my request to provide numerical metrics of read density in the heatmaps (which I still disagree with) it is impossible to tell if the differences between the MNase coverage and ChEC-Seq are simply the result of coverage imbalances. I attempted to get this information from one of the supplementary tables, however the MNase experiments are

not listed in the table. Thus, I cannot conclude much from these data as presented.

Controls should be presented far more clearly and unambiguously.

The results we present are neither surprising nor inconsistent with our claims. Figure S5, whose 48 heat maps we have now supplemented with 48 traces quantifying read depths in those heat maps, shows fragments in the 50-100 base pair range, where footprints of transcription and replication factors appear. MCM-ChEC is expected to reveal the MCM complex and only the MCM complex, while free MNase controls should reveal both the MCM complex and any other DNA binding proteins that happen to be present. In cases where no other proteins are present, the free MNase control should look like the MCM-ChEC sample. An example of this, taken from Figure S5, is ARS432 (right column in figure below): In this case, the free MNase sample (bottom) looks like the three MCM-ChEC samples, ***because the MCM complex is the only factor bound***. The more non-MCM proteins that are present, and the higher the fraction of cells that contain such proteins, the less similar the MCM-ChEC samples and free MNase controls should look to each other. Examples of this, also taken from Figure S5, include (1) ARS452, which is bound by the MCM complex and one other factor, whose abundance is similar to that of MCM. Therefore, the MCM complex appears in all four samples, while the other factor appears exclusively in the free MNase. (2) ARS430, which is bound by MCM and two other factors, one more abundant than MCM and one less. And (3) ARS431, which is bound by MCM and one other factor, whose abundance is high enough, relative to that of MCM (which is low, as can be seen in the quantitation of the entire area in Figure 4B), as to overwhelm the signal from MCM. All of this is exactly what is expected from these techniques.

From S5 Fig.

The "unusually low background" is also the result that one expects when fusing a subunit of the MCM complex to MNase; indeed, to achieve such low background, with only the MCM complex revealed, is precisely the reason that we created the fusion protein rather than simply using exogenously added MNase.

In addition to being expected, our results cannot be "simply the result of coverage imbalances". Sequencing a larger or smaller number of reads cannot alter the ***percentage*** of those reads that arise from different sequences in the library. For each of the 12 origins shown in both Figure 4 and Figure S5, we quantified the percentage of signal that comes from the MCM complex, and we wrote those percentages on the quantitative traces under each heat map in Figure 4. It is not possible to radically change these percentages by altering sequencing coverage, whether by over- or under-sequencing, so the difference between the MCM-ChEC samples and the free MNase controls - which, again, is ***expected*** to fluctuate with variation in binding of non-MCM proteins - cannot be the result of "coverage imbalances" between the two samples.

3. The authors state that:

“Of particular note is our observation that they do not show the preferential association with replication initiation sites over replication termination sites that we see with Mcm-ChEC in S pombe (S10 Fig) and HeLa cells (S16 Fig).”

I would caution the authors that while replication initiates at relatively well defined loci, termination seems likely to be more stochastic, spread across a far wider region than initiation is in most cases. Thus, these side-by-side comparisons of initiation vs termination regions should be taken with this precaution in mind and are not suitable as the primary evidence that their experiments are working.

The context of our remark quoted by the reviewer is the following: We had been asked to compare the results we obtained with MCM2-ChEC in pombe and in humans with corresponding results obtained with free MNase rather than MCM2-ChEC. The purpose of this control was to confirm that MCM2-ChEC in these organisms truly revealed the locations of MCM binding rather than simply broad patterns of MNase-sensitivity. In other words, this control was aimed at eliminating the possibility that, even though our MCM2-ChEC signal was exactly where it was expected to be, based on results with Pu-seq (S. pombe) and Okazaki-seq (human), which was high in replication initiation zones and low in replication termination zones, this gratifying result was fortuitous. We provided these data and pointed out that, not only were the results with free MNase overall different from the results with MCM2-ChEC, but they were different where it was most critical, which was in replication initiation and termination zones. The reviewer is correct that replication termination zones are broader than replication initiation zones, but this does not obviate the importance of focusing on these regions in evaluating the results of the free MNase control that we were asked to supply.

4. The authors provide numerous scatterplots to compare ChEC-Seq to free MNase. These comparisons raise multiple questions. First, the authors should explicitly explain what exactly they are plotting. Is it all reads, or reads of a particular size?

We have clarified in the legends to figures S2, S13 and S17 that we are plotting all reads and not just reads of a particular size.

In cerevisiae (Fig. S2), the scatterplots are generated using 100 bp windows, which is reasonable, as it reflects the scale of the expected ChEC-Seq signal. However, in other organisms, the authors have performed these comparisons in 10Kb bins across the entire genome. This is the wrong scale for this comparison and much of the correlation (or lack thereof) is potentially driven by reads derived from the genomic background (S13, S17).

If our coverage in yeast and mammals were equal, it would be appropriate to bin the data equally. However, the fact that the human genome is about 300 times bigger than the yeast genome, while we have only about 5 times more reads for the human genome, makes binning the human at 10 kb while binning the yeast genome at 100 base pairs more appropriate. In other words, we are matching the samples for coverage per bin rather than for bin size.

Finally, the correlations for ChEC-Seq are all a little unusual because they appear to lack any stochastic genomic background. Is this the result of some data processing not described in the figure legend? **Such background appears to be absent in the heatmaps too but is** **not explained by the authors.** This point was raised, but not addressed in my initial review. Importantly, it is not clear how much the correlation with MNase-Seq would= improve if their analyses were restricted to just the places where MCM-ChEC-Seq shows a “peak” or signal of some sort. This should be explored in depth.

As we have discussed above, in the distribution plots such as that in Fig 1C, it is clear that almost all the signal emanates from with 80 bp from the signal center, flanked by green lines. The absence of signal in the region outside the green lines indicates the absence of Mcm2-MNase cutting in this region; indeed the lack of cutting is the explanation that the “**background appears to be absent in the heatmaps”**. We corroborated this absence of cutting independently both with a Southern blot, an experiment that was previously suggested by the reviewer 2, and by qPCR results in Figure 3. These results show that cutting occurs exclusively, or almost exclusively, in the immediate vicinity of the green lines. Furthermore we provide the files with raw read numbers for each bp as well as normalized reads in S1 Source Files.

5. The authors do not show any data for replicates at high resolution. This is essential to validate the consistency of their technique.

We have discussed the resolution for the replicas in mammalian cells under point number 4 above.

Figure S3:

The data for individual panels are not the same as the grouped panel. In particular, the MCM6 data are shifted.

The reviewer is correct that there are subtle shifts in the fragment size distributions between the MCM2-ChEC, MCM4-ChEC and MCM6-ChEC S. cerevisiae sequencing libraries. This is not surprising, since fusions between MNase and different subunits of the MCM helicase complex will not necessarily result in MNase contacting the DNA at precisely the same distance from the center of the complex. Furthermore, these differences do not affect our conclusion that the size distributions in the three libraries are similar enough to conclude that they are consistent with the tagging of three different subunits in the same complex.

Reviewer 1; point 6:

Figure S12B: The authors provide a new figure that helps to better infer the MCM2 ChEC-Seq fragment length in different species. This figure corroborates the issue I raised in my review but the differences between species are still not discussed by the authors.

There are subtle differences in cutting dynamics between the species.

Reviewer 1; point 7:

The authors agree that their method cannot distinguish between double MCM peaks and multiple binding sites for single MCMs. Nonetheless, they persist in stating that an advantage of ChEC-Seq is precisely that they can do this analysis, both in the legend of S6: “indicates that the two MCM DH footprints at this origin mostly reflect signals that emanate from two populations of cells, each with a single MCM DH” and in the introduction:

“Mcm ChIP, while adequate for determining the relative MCM abundance among the origins,lacks the necessary resolution to unambiguously resolve the exact number of loaded MCM complexes. The high resolution of the ChEC technique overcomes this barrier and thus enabled us to address the question directly”

Similar comments are also made in the discussion and should be rectified.

Two different results are getting mixed up here. The high resolution of the ChEC technique allows us to count the number of distinct sites at which MCM double hexamers are bound at individual origins. At approximately half of the origins in *S. cerevisiae*, there is just one such site. In those cases, we can unambiguously conclude that there is at most one complex loaded in any individual cell. This unambiguous conclusion, which applies to half of all genomic origins, was made possible by the high resolution of the ChEC technique. The rest of the origins in *S. cerevisiae* show multiple binding sites. In those cases, there is ambiguity about whether those multiple sites indicate that multiple complexes are present in single cells or instead that single complexes are bound at different sites in different cells. However, although there is ambiguity, we point out that if multiple complexes were bound in single cells, we would expect to see a 75 base pair diagnostic fragment in our sequencing libraries that we do not see. We acknowledge that this does not constitute unambiguous proof that multiple MCM complexes are *not* present in single cells, because "... we cannot exclude the possibility that the 75 bb fragment could have been degraded" (from legend to Figure S6).

Review point 11:

The authors have not performed this analysis and have ignored this suggestion. Instead, and despite their acknowledgement that ChEC-Seq cannot make inferences about binding multiplicity, the authors persist in claiming that:“Our results contradict the previous conclusions by Das et al., and we now note this in the

Discussion.”

Das et al. showed that multiple MCMs appear to load at yeast origins. At one origin they studied in detail (ARS1; aka ARS416), they observed that three MCM complexes load at this locus on each plasmid in their system. A quick glance at Figure S4 shows multiple (2-4) peaks at this locus using ChEC-Seq. Given these data together, I would suggest that in contrast to the assertions of the authors, the ChEC-Seq experiment may instead be showing exactly the expected pattern if multiple MCMs load at the same time.

From S4 Figure (1 out of 5), last row, first two columns.

We apologize for the error in ARS labeling in the Figure S4 (1 of 5), that contain ARS416, and for not adequately addressing the findings of Das et al [6]. The ARS labeling in the last two rows of Fig S4A and the first row of Fig S4B were inadvertently transposed. We have now corrected this error.

The results presented by Das et al. indicate that multiple MCMs bind to a plasmid containing a single ARS416 origin. Insufficient resolution of MCM ChIP was the exact reason for using a plasmid purification assay in that work, and not relying on genomic ChIP. However, our results using MCM2-ChEC from the Fig S4A clearly show that genomic ARS416 contain a single MCM DH.

Review point 12: (Fig 6)

The authors have inexplicably provided variance estimates for the figure as a separate table

instead of as error bars. I understand that the standard deviations are huge relative to the mean, but that is exactly why they should be shown on the graph! A boxplot would be a better solution and this should be remedied. Also, it is not clear why the inclusion of other chromosomes would make “computational analyses simpler and less error-prone”. Although this is commendably honest, the authors should explain what “errors” arise when using other chromosomes?

We have repeated the analysis with the entire genome and provided the results in Figure 6A as a box plot.

Finally, the numerical values on the y-axis still do not match the (revised) numbers in the figure legend.

We have provided the numerical values in the figure legend and made sure that these results match those in the figure.

Reviewer 1; point 15:

This point was modified in one place in the text but not others. It should be checked throughout.

We now mention the comparison of tagged and untagged MCM at three places:

Page 10:

To do so, we expressed human Mcm2 tagged with MNase in HeLa cells using a lentiviral vector, choosing an expression level that does not exceed endogenous Mcm2 levels (S11 Fig).

Page 14:

To compare MCM distribution and abundance between the two X chromosomes, we expressed mouse Mcm2 tagged with MNase at the C-terminus using a lentiviral vector at a level that does not exceed endogenous protein (S11 Fig.)

In Figure legend:

**S11 Fig** Immunoblot showing expression level of MCM-MNase fusion protein in HeLa and mouse hybrid *M.musculus/M.castaneus* Patski cells. The relative abundance of tagged vs untagged bands for human and mouse cells was 0.4- and 0.25- fold, respectively.

17. Page 7-10 & throughout paper:

The authors refer to Mcm2-7 footprints. In S. cerevisiae , their data broadly support this type of generalization, yet in S. pombe , Hela and Patski cells, they only have data for Mcm2. They should be more explicit in describing their data in these organisms and not generalize to all MCMs.

We have now clarified that the term “Mcm2-7” footprints refers to a footprint of the MCM complex and not the footprint of individual complex components.

From the abstract:

“To determine the degree to which distribution of pre-RC loading alone could account for chromosomal replication patterns, we mapped the binding sites of the Mcm2-7 helicase complex (MCM) in budding yeast, fission yeast, mouse and humans.”

In the text we have replaced Mcm2-7 with MCM.

Reviewer 1; point 18:

The authors have neither addressed my request to examine MCM2-ChEC Seq at high

resolution in human cells, nor explained why they have ignored this request. High resolution proxies for origins of replication in human include ORC2 ChIP-Seq [6], Ini-Seq [7] and SNS-Seq in HeLA cells [8](GSE134988). SNS-Seq data from mouse are also available (Cayrou et al., 2015) & (Almeida et al., 2018). These analyses are essential to understand if these experiments worked in human & mouse cells. As mentioned above, it is not clear if published MNase data are an apt control for ChEC-Seq.

We carried additional analyses in HeLa cells suggested by the reviewer. In Figure 7 we show the localization of centers of initiation and termination zones as well as Orc2 ChIP-seq [3], ini-seq peaks [4], and SNS [5]. As we pointed out earlier, the centers of initiation and termination zones generally coincide with MCM maxima, where as centers of termination zones coincide with local MCM minima. As previously reported, there is only a partial overlap between OK-seq, ini-seq and SNS-seq peaks. However, even when analyzed at a 1 kb scale we found significant co-localization of MCM abundance with Orc-ChIP, ini-sq and SNS peaks. Our analysis of the mouse data is narrowly focused on the replication of the inactive X-chromosome and we felt that adding similar analysis of the mouse data would be redundant and would distract from the main issue we were focus on in the mouse cell model.

From figure 7

Reviewer 1; point 19:

In human, although some initiation appears to occur in zones spanning tens of Kb, experiments such as those mentioned above also identify discrete sites that represent putative origins of replication of <1 Kb in width. These could be assessed.

In point 18, the reviewer mentions three high resolution studies aimed at identifying the sites of replication initiation in human cells. These studies used Orc-ChIP, ini-seq and SNS, and in each case the authors reported peaks of signal that were in the 500 base pair - 1 kb range. As mentioned above, we compared our MCM2-ChEC data to each of these data sets as follows:

1. bin the entire genome in 1 kb bins
2. extract two groups of 1 kb bins, those that overlap peaks, as defined by the authors, and those immediately flanking those peaks
3. do a 2-sample t-test to compare these two groups
4. the result, in all three cases, is a 2-tailed p value < 10e-20

Note that by using the bins immediately flanking the high resolution peaks as one of the two groups, we are asking whether, at a resolution of 1 kb, our MCM2-ChEC data are consistent with the results of these three studies. As mentioned above, we have included these results in the text and in figures 7A and S14A-F.

I also disagree that showing mouse data in Figure 2A is redundant. There is no figure that shows high-resolution mouse data, analogous to figure 2A (top). Given the high-resolution nature of the ChEC-Seq protocol, this is essential.

We have added a heat map for mouse in S16C Figure.

Reviewer 1; point 22:

The authors have not responded to my questions.

From Previous review.

((22). The authors conclude that a single MCM DH likely loads at most origins in every cell. This is OK, but I would be interested in knowing the authors’ opinion on potential biases in the MCM-ChEC-Seq method; for instance, the authors allude to an ambiguous PCR bias for shorter fragments on Page 6. Is bead-based size selection used for ChEC-Seq as per the original protocol? Is it possible that the method disproportionately excludes fragments derived from loci at which multiple, closely spaced MCM complexes are loaded? If two MCM DH complexes sit adjacent to each other, the C-termini MNase tags will potentially abut; can the MNase always cut in between? Perhaps these caveats could be discussed.)

Any DNA size-based purification is bound to introduce biases; we were particularly worried about its potential for loss of the small fragments. This is the main reason that we proceeded to ligating adapters without any purification. Preferential amplification of small fragments allowed us to focus our analysis on small DNA fragments, which is exactly where we expected MCM footprints to be.

Is it possible that densely packed MCM DH might obstruct the cutting next to its abutting neighbor? We do not think this is likely. First, unless there is an obstacle such as a nucleosome or some other DNA binding protein, MCM can easily slide off DNA. Thus, we would expect that sufficient space would be generated for the adjacent MCM to cut as soon as there is a cut of the MCM at the edge of the putative MCM array. Second, we see MCM2-MNase cutting very close to nucleosomes, even in cases where nucleosome free regions are small. Lastly, we generally observe that cutting is fast (most of the cutting at ARS1103 is completed within 15-30 seconds after MNase activation with calcium. Almost all the footprints that we see at 30 seconds are the same that we see after 5 minutes. If cutting is inhibited at these putative MCM arrays, we would expect that the number of footprints would increase with time, and we do not see that.

Reviewer 1; point 23: This point still requires clarification.

From previous review:

(Point 23. Page 14: The 100 kb initiation zone

What is the “100 kb initiation zone in mammals”?)

We have expanded the description of the initiation and termination zones as follows:

“Previous studies have shown that at a finer scale, replication within a single early- or late-replicating region is not uniform; for example, studies using Okazaki fragment sequencing (OK-seq) and BrdU incorporation have shown that replication initiation within these regions is concentrated in the broad zones encompassing from 10 kb and to up 150 kb, that are typically located upstream of transcribed genes, whereas termination occurs within even broader zones [49]. Using OK-seq in HeLa cells, Petryk and al. identified 12,000 initiation and 18,000 termination zones with median and mean sizes of 21 and 25 kb for initiation zones and 24 and 74 kb for termination zones [49]. In a fork directionality plot, such as that in Fig 7A, such replication initiation zones are centered at the (from left to right) blue-to-red transition sites, whereas the termination zones are centered at red-to-blue transition sites; such blue-to-red and red-to-blue transitions indicate sites of divergence and convergence, respectively, of leading- and lagging strand polymerases.”

1.	Petryk N, Kahli M, d'Aubenton-Carafa Y, Jaszczyszyn Y, Shen Y, Silvain M, et al. Replication landscape of the human genome. Nat Commun. 2016;7:10208. Epub 2016/01/12. doi: 10.1038/ncomms10208. PubMed PMID: 26751768; PubMed Central PMCID: PMCPMC4729899.

2.	Daigaku Y, Keszthelyi A, Muller CA, Miyabe I, Brooks T, Retkute R, et al. A global profile of replicative polymerase usage. Nat Struct Mol Biol. 2015;22(3):192-8. Epub 2015/02/11. doi: 10.1038/nsmb.2962. PubMed PMID: 25664722; PubMed Central PMCID: PMCPMC4789492.

3.	Miotto B, Ji Z, Struhl K. Selectivity of ORC binding sites and the relation to replication timing, fragile sites, and deletions in cancers. Proc Natl Acad Sci U S A. 2016;113(33):E4810-9. Epub 2016/07/21. doi: 10.1073/pnas.1609060113. PubMed PMID: 27436900; PubMed Central PMCID: PMCPMC4995967.

4.	Langley AR, Graf S, Smith JC, Krude T. Genome-wide identification and characterisation of human DNA replication origins by initiation site sequencing (ini-seq). Nucleic Acids Res. 2016;44(21):10230-47. Epub 2016/09/03. doi: 10.1093/nar/gkw760. PubMed PMID: 27587586; PubMed Central PMCID: PMCPMC5137433.

5.	Long H, Zhang L, Lv M, Wen Z, Zhang W, Chen X, et al. H2A.Z facilitates licensing and activation of early replication origins. Nature. 2020;577(7791):576-81. Epub 2019/12/27. doi: 10.1038/s41586-019-1877-9. PubMed PMID: 31875854.

6.	Das SP, Borrman T, Liu VW, Yang SC, Bechhoefer J, Rhind N. Replication timing is regulated by the number of MCMs loaded at origins. Genome Res. 2015;25(12):1886-92. Epub 2015/09/12. doi: 10.1101/gr.195305.115. PubMed PMID: 26359232; PubMed Central PMCID: PMCPMC4665009.
