# Supplementary material for: Chromosomal Mcm2-7 distribution and the genome replication program in species from yeast to humans
Source: PLoS Genet. 2021 Sep 2;17(9):e1009714. doi: 10.1371/journal.pgen.1009714 (PMC8443269; doi:10.1371/journal.pgen.1009714)
Supplement: Attachment — Submitted filename: Response_revision_July_2021.docx [file pgen.1009714.s039.docx]

Dear Dr Bedalov,

Thank you very much for submitting your Research Article entitled 'Chromosomal Mcm2-7 distribution and the genome replication program in species from yeast to humans' to PLOS Genetics.

The manuscript was fully evaluated at the editorial level, and I believe that you have done an excellent job of addressing the previous concerns, so I see no point in sending the manuscript out for review again. However, we identified some minor concerns that we ask you address in a revised manuscript. This should be relatively easy to do.

First, thank you for meeting requests for full methods details and for supplying underlying data. There are just a few changes that need to be made:

1. Figure 3B, underlying data. I could not find the numerical data underlying this graph in source_files_for_Figure1_to_Figure7A.zip. Please include it.

We have included Figure 3B data in the S1 Source Files.

2. Source files for figures. Thank you for including these, which I know must have taken a lot of work. These files should be renamed something like "S1_source_files_for_Figure1_to_Figure7A.zip" (to pick an example), "S2_source_files_for_Figure7B_part1_of_2.zip" etc. etc. (see [journals.plos.org/plosgenetics/s/supporting-information](http://journals.plos.org/plosgenetics/s/supporting-information) for further information). Then, each Sx source file should be listed in the manuscript along with the other supplementary files (i.e. on page 49 and following of the current manuscript. For example:

Supplemental information:

S1_source_files_for_Figure1_to_Figure7A.zip  Zip file containing the following: data underlying Figure 1A, B, C, and code example; data underlying Figure 2A, B, C;....etc

We have renamed the Source files as S1 to S12 and listed their content in the manuscript as requested.

3. In the data availability statement, please add that numerical data and code underlying all figures can be found in supporting information files S1-S12.

The following statement is included:

Numerical data for generating figures and the code are available in the Supporting Source Files S1 to S12.

In addition we ask that you:
1) Provide a detailed list of your responses to the review comments and a description of the changes you have made in the manuscript. 
2) Upload a Striking Image with a corresponding caption to accompany your manuscript if one is available (either a new image or an existing one from within your manuscript). If this image is judged to be suitable, it may be featured on our website. Images should ideally be high resolution, eye-catching, single panel square images. For examples, please browse our [archive](https://urldefense.proofpoint.com/v2/url?u=https-3A__journals.plos.org_plosgenetics_volume&d=DwMGaQ&c=eRAMFD45gAfqt84VtBcfhQ&r=v2OLxwF1QhJd633WJXHa80Y4xpeZHjONCdo5Te6LOtU&m=yHVYyKxtE2kYd19FGoCRWATT-lLFDZX_8lY_NTkwNLE&s=5cYggXhTHkkzilPT3eA4c7esAtTqHp_BiXIzge6Btvw&e=). If your image is from someone other than yourself, please ensure that the artist has read and agreed to the terms and conditions of the Creative Commons Attribution License. Note: we cannot publish copyrighted images.

We hope to receive your revised manuscript within the next 30 days. If you anticipate any delay in its return, we would ask you to let us know the expected resubmission date by email to [plosgenetics@plos.org](mailto:plosgenetics@plos.org).

If present, accompanying reviewer attachments should be included with this email; please notify the journal office if any appear to be missing. They will also be available for download from the link below. You can use this link to log into the system when you are ready to submit a revised version, having first consulted our [Submission Checklist](https://urldefense.proofpoint.com/v2/url?u=http-3A__journals.plos.org_plosgenetics_s_submit-2Dnow-23loc-2Dsubmission-2Dchecklist&d=DwMGaQ&c=eRAMFD45gAfqt84VtBcfhQ&r=v2OLxwF1QhJd633WJXHa80Y4xpeZHjONCdo5Te6LOtU&m=yHVYyKxtE2kYd19FGoCRWATT-lLFDZX_8lY_NTkwNLE&s=0niyJpb7qaFT4U5ZpRkZX78GopntUhXVRxbuUILhxU0&e=).

While revising your submission, please upload your figure files to the [Preflight Analysis and Conversion Engine](https://urldefense.proofpoint.com/v2/url?u=http-3A__pace.apexcovantage.com_&d=DwMGaQ&c=eRAMFD45gAfqt84VtBcfhQ&r=v2OLxwF1QhJd633WJXHa80Y4xpeZHjONCdo5Te6LOtU&m=yHVYyKxtE2kYd19FGoCRWATT-lLFDZX_8lY_NTkwNLE&s=wIIAuzZVxMtpS5sAhLC0ouMe2ZgVIgxkMEaU1RFsYKk&e=) (PACE) digital diagnostic tool. PACE helps ensure that figures meet PLOS requirements. To use PACE, you must first register as a user. Then, login and navigate to the UPLOAD tab, where you will find detailed instructions on how to use the tool. If you encounter any issues or have any questions when using PACE, please email us at [figures@plos.org](mailto:figures@plos.org).

Please be aware that our [data availability policy](https://urldefense.proofpoint.com/v2/url?u=http-3A__journals.plos.org_plosgenetics_s_data-2Davailability&d=DwMGaQ&c=eRAMFD45gAfqt84VtBcfhQ&r=v2OLxwF1QhJd633WJXHa80Y4xpeZHjONCdo5Te6LOtU&m=yHVYyKxtE2kYd19FGoCRWATT-lLFDZX_8lY_NTkwNLE&s=fjCKw9Pi415CEZJdMnalUSWH0GhQFYKZXcEZ5qa-2BU&e=) requires that all numerical data underlying graphs or summary statistics are included with the submission, and you will need to provide this upon resubmission if not already present. In addition, we do not permit the inclusion of phrases such as "data not shown" or "unpublished results" in manuscripts. All points should be backed up by data provided with the submission.

To enhance the reproducibility of your results, we recommend that you deposit your laboratory protocols in [protocols.io](http://protocols.io/), where a protocol can be assigned its own identifier (DOI) such that it can be cited independently in the future. Additionally, PLOS ONE offers an option to publish peer-reviewed clinical study protocols. Read more information on sharing protocols at [https://plos.org/protocols?utm_medium=editorial-email&utm_source=authorletters&utm_campaign=protocols](https://urldefense.proofpoint.com/v2/url?u=https-3A__plos.org_protocols-3Futm-5Fmedium-3Deditorial-2Demail-26utm-5Fsource-3Dauthorletters-26utm-5Fcampaign-3Dprotocols&d=DwMGaQ&c=eRAMFD45gAfqt84VtBcfhQ&r=v2OLxwF1QhJd633WJXHa80Y4xpeZHjONCdo5Te6LOtU&m=yHVYyKxtE2kYd19FGoCRWATT-lLFDZX_8lY_NTkwNLE&s=HNYfqlzD_OPUYti_Xi4x_rH2Q3UiTyoIstb2HwpR5uQ&e=)

Please review your reference list to ensure that it is complete and correct. If you have cited papers that have been retracted, please include the rationale for doing so in the manuscript text, or remove these references and replace them with relevant current references. Any changes to the reference list should be mentioned in the rebuttal letter that accompanies your revised manuscript. If you need to cite a retracted article, indicate the article’s retracted status in the References list and also include a citation and full reference for the retraction notice.

PLOS has incorporated [Similarity Check](https://urldefense.proofpoint.com/v2/url?u=http-3A__www.crossref.org_crosscheck.html&d=DwMGaQ&c=eRAMFD45gAfqt84VtBcfhQ&r=v2OLxwF1QhJd633WJXHa80Y4xpeZHjONCdo5Te6LOtU&m=yHVYyKxtE2kYd19FGoCRWATT-lLFDZX_8lY_NTkwNLE&s=D77Zy-wrxYITlKK3_0gfdc6E2fjYM1NAJX2JTr6VdtI&e=), powered by iThenticate, into its journal-wide submission system in order to screen submitted content for originality before publication. Each PLOS journal undertakes screening on a proportion of submitted articles. You will be contacted if needed following the screening process.

To resubmit, you will need to go to the link below and 'Revise Submission' in the 'Submissions Needing Revision' folder.


Please let us know if you have any questions while making these revisions.

Yours sincerely,

Michael Lichten, Ph.D.
Associate Editor
PLOS Genetics

Gregory P. Copenhaver
Editor-in-Chief
PLOS Genetics
